# Indium‐Doping Advances High‐Performance Flexible Ag_2_Se Thin Films

**DOI:** 10.1002/advs.202500364

**Published:** 2025-03-17

**Authors:** Tianyi Cao, Xiao‐Lei Shi, Boxuan Hu, Siqi Liu, Wanyu Lyu, Meng Li, Sen Wang, Wenyi Chen, Wei‐Di Liu, Raza Moshwan, Tuquabo Tesfamichael, Jennifer MacLeod, Zhi‐Gang Chen

**Affiliations:** ^1^ School of Chemistry and Physics ARC Research Hub in Zero‐emission Power Generation for Carbon Neutrality, and Centre for Materials Science Queensland University of Technology Brisbane Queensland 4000 Australia; ^2^ School of Mechanical, Medical and Process Engineering Queensland University of Technology Brisbane Queensland 4001 Australia

**Keywords:** Ag_2_Se, device, film, flexible, thermoelectric

## Abstract

Enhancing the thermoelectric performance of Ag_2_Se thin films via physical vapor deposition remains challenging. In this study, a precursor doping strategy is introduced to fabricate In‐doped Ag_2_Se thin films. In substitutional doping at the Ag cation sites increases the charge density distribution of Ag_2_Se, improving electrical conductivity, while maintaining a high Seebeck coefficient and relatively low thermal conductivity. This approach yields a competitive room‐temperature power factor of ≈26.3 µW cm^−1^ K^−2^ and a *ZT* value approaching 1. The films, supported by a polyimide substrate and optimized for thickness, exhibit uniform composition and excellent flexibility, retaining over 90% of their initial electrical conductivity after 500 bending cycles with a 5 mm bending radius. Additionally, a five‐leg flexible thermoelectric device constructed from these films achieves a power density of up to 630.6 µW cm^−2^ under a temperature difference of 18 K, corresponding to a normalized power density of nearly 2 µW cm^−2^ K^−2^, highlighting its potential for practical applications.

## Graphical abstract

A “precursor doping strategy” is employed to realize In‐doping at Ag sites in Ag_2_Se thin films, boosting electrical conductivity while maintaining a high Seebeck coefficient, therefore achieving a high‐power factor of ≈26.3 µW cm^−1^ K^−2^ and ZT of ≈1. The flexible device based on these films delivers a power density of 630.6 µW cm^−2^ under ΔT = 18 K.

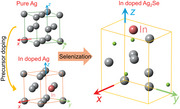

## 1 Introduction

Flexible thermoelectric materials and devices, which utilize the Seebeck effect to convert the temperature difference between the human body and the environment into electrical energy, offer a promising solution for lightweight and stable power sources in portable electronics.^[^ ^1^, ^2^ ^]^ Optimizing thermoelectric performance of thermoelectric materials is crucial for increasing conversion efficiency (*η*) and improving device practicality. The overall performance of thermoelectric materials is evaluated by the figure of merit, *ZT*, defined as:^[^ ^3^ ^]^

(1) $$\;ZT=\frac{S^{2}\sigma }{\kappa }T=\frac{S^{2}\sigma }{\kappa_{e}+\kappa_{l}}T$$

where *S* is the Seebeck coefficient, *σ* is the electrical conductivity, *S* ^2^ *σ* is the power factor, *κ* is the thermal conductivity, *κ*_e_ is the electronic thermal conductivity, *κ*_l_ is the lattice thermal conductivity, and *T* is the absolute temperature.^[^ ^4^, ^5^, ^6^ ^]^ According to this formula, *ZT* is determined by *S*, *σ*, and *κ*_e_, which are highly coupled to the carrier concentration (*n*). Tuning *n* is an effective approach to optimizing the overall *S* ^2^ *σ* and *ZT*. To achieve this, the introduction of appropriate dopants can be used to modulate the band structure,^[^ ^7^ ^]^ effectively tuning the *n*. Additionally, incorporating a suitable secondary phase can induce an energy filtering effect at the phase boundaries,^[^ ^8^ ^]^ decoupling *S* and *σ*.^[^ ^9^ ^]^ The lattice thermal conductivity, *κ*_l_, is primarily governed by phonon transport. The introduction of secondary phases and dopants can disrupt the lattice structure by introducing various dimensional lattice defects to effectively scatter phonons with different wavelengths, ultimately reducing *κ*_l_.^[^ ^10^, ^11^, ^12^ ^]^ Despite decades of research and advancements in optimizing thermoelectric parameters, with reported *η* values exceeding 15%,^[^ ^13^ ^]^ a considerable gap remains before widespread practical applications will be achieved.

Wearable flexible thermoelectric devices should operate efficiently under near‐room‐temperature conditions and with relatively low temperature difference (Δ*T*). Consequently, the materials used in these devices require excellent thermoelectric performance and mechanical flexibility within this temperature range.^[^ ^3^ ^]^ Currently, various candidates for near‐room‐temperature applications include Bi_2_Te_3_‐based materials,^[^ ^14^ ^]^ Mg_3_Bi_2_‐based materials,^[^ ^15^ ^]^ and n‐type Ag_2_X^[^ ^16^ ^]^ and p‐type AgCuX‐based materials,^[^ ^17^ ^]^ where X represents S, Se, or Te. Bi_2_Te_3_ remains the best‐performing thermoelectric material in this range and is the only one widely commercialized.^[^ ^18^, ^19^ ^]^ It has been reported that Bi_2_Te_3_‐based single‐crystal materials can effectively disperse stress by introducing a large number of antisite defects, enabling a transition from brittleness to flexibility while maintaining thermoelectric performance comparable to that of polycrystalline brittle Bi_2_Te_3_‐based materials.^[^ ^20^ ^]^ However, single‐crystal Bi_2_Te_3_ is limited by its high cost and complex fabrication process, which significantly constrain its practical applications where flexibility is required. Mg_3_Bi_2_‐based materials have shown rapid development and strong potential for room‐temperature applications,^[^ ^11^ ^]^ but their synthesis poses significant challenges. For example, magnesium's reactivity with glass necessitates arc‐welded tantalum tubes during melt synthesis,^[^ ^21^ ^]^ and specialized ball mills are required to prevent magnesium powder ignition during mechanical alloying.^[^ ^22^ ^]^ Additionally, rendering Mg_3_Bi_2_‐based materials flexible remains difficult.^[^ ^11^ ^]^ Given these challenges, Ag_2_X materials, particularly Ag_2_Se, offer a promising alternative due to their relatively lower cost and high thermoelectric performance.^[^ ^16^ ^]^ Among Ag_2_X compounds, Ag_2_Se exhibits the highest thermoelectric performance. In addition, bulk Ag_2_Se has also shown great practical potential in recent years.^[^ ^23^, ^24^, ^25^, ^26^, ^27^, ^28^, ^29^ ^]^ However, intrinsic Ag_2_Se achieves its high *S* ^2^ *σ* primarily through elevated *σ* rather than *S*, limiting its utilization of Δ*T* compared to other materials.^[^ ^30^ ^]^ Isoelectronic element doping can address this by balancing ductility and thermoelectric performance in Ag_2_Se.^[^ ^16^ ^]^ Bulk Ag_2_Se materials have demonstrated exceptional performance, with room‐temperature power factors exceeding 32 µW cm^−1^ K^−2^ and *ZT* values surpassing 1.1.^[^ ^26^ ^]^ Similarly, Ag_2_Se thin films exhibit room‐temperature power factors exceeding 25 µW cm^−1^ K^−2^ with reports indicating *ZT* values approaching or even exceeding 1.^[^ ^30^, ^31^, ^32^ ^]^ These properties highlight the potential of Ag_2_Se for wearable thermoelectric applications.

The application of Ag_2_Se in near‐room‐temperature flexible thermoelectric devices faces significant challenges.^[^ ^33^ ^]^ First, optimization methods for Ag_2_Se are limited. For bulk Ag_2_Se, optimization is relatively straightforward and typically involves doping at chalcogenide anion sites (self‐doping)^[^ ^31^ ^]^ or Ag cation sites.^[^ ^34^ ^]^ However, most elements lack a +1 valence state, and introducing excess electrons often increases the *n*, thereby reducing the *S*.^[^ ^26^, ^35^ ^]^ Ag_2_Se's intrinsic electron‐rich nature further limits the effectiveness and variety of doping strategies. In some cases, doping can alter the primary phase (e.g., in anion‐site solid solutions), sacrificing room‐temperature performance for overall thermoelectric efficiency.^[^ ^35^ ^]^ Second, the use of bulk Ag_2_Se in flexible thermoelectric devices is constrained compared to thin‐film‐based materials, which offer better adaptability to flexible applications. However, thin‐film optimization is even more restricted. Practical doping approaches are scarce due to differences in preparation methods between bulk and thin film Ag_2_Se materials. While reports indicate that achieving (013) orientation in thin films can improve carrier mobility (*μ*) and *σ*,^[^ ^32^, ^36^ ^]^ the limited tunability of *S* often leads to excessive *κ*_e_, ultimately lowering *ZT*. Achieving freely adjustable orientations remains a technical challenge. Furthermore, current studies on doping Ag_2_Se thin films are limited and primarily focused on anion‐site solid solution doping (S, Se, and Te).^[^ ^31^ ^]^ For instance, Se self‐doping can slightly enhance *S* while maintaining *σ*, whereas S doping reduces both *S* and *σ* but improves flexibility.^[^ ^37^ ^]^ Overall, research on doping elements in Ag_2_Se thin films is still in its infancy, with most efforts centered on isoelectronic element doping at anion sites. This highlights the significant untapped potential for further investigation.

In this study, we combined an In‐doped Ag‐precursor strategy with a solution‐based thermal selenization method (details in Supporting Information, Chemical Reaction Principles section). Using the thickness monitor in an electron beam (E‐beam) evaporation system, the evaporation amount of In was controlled to obtain Ag_1.98_In_0.02_Se thin films. These films exhibited a high *μ* of 1580 cm^2^ V^−1^ s^−1^ and high *σ* of 1462 S cm^−1^, resulting in a competitive room‐temperature *S* ^2^ *σ* of 26.3 µW cm^−1^ K^−2^. In‐doping introduced additional charge carriers into the Ag_2_Se system without significantly increasing *κ*. Structural characterization revealed long crystal slip regions and an amorphous Ag_2_Se phase, inducing extensive phonon scattering maintaining a relatively stable *κ* despite enhanced *σ*. Additionally, the polyimide (PI) substrate provided excellent flexibility. To demonstrate its practical potential, a flexible thermoelectric device was fabricated using the In‐doped Ag_2_Se thin films. Under a Δ*T* of 18 K, the device achieved an output power (*P*) of 0.21 µW and a high‐power density (*ω*) of 630.6 µW cm^−2^, corresponding to a normalized power density (*ω*_n_) of 1.95 µW cm^−2^ K^−2^, exhibiting considerable application potential.

## 2 Results and Discussion

**Figure 1:**
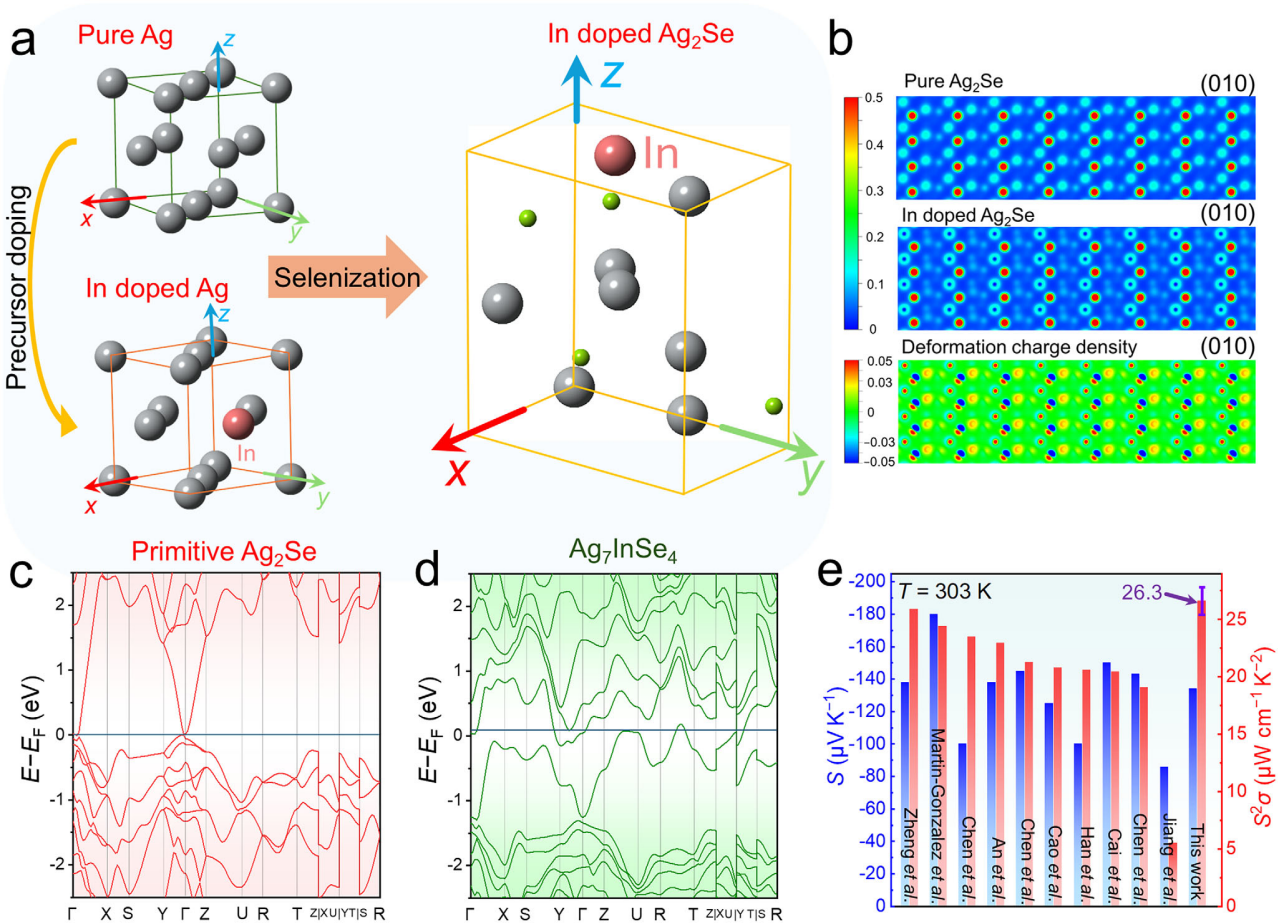
Introduction of In‐doped Ag_2_Se thin film. a) Schematic diagram of the unit‐cell structure of the In‐doping in Ag precursor and the selenization process to form the of In‐doped Ag_2_Se. b) Charge distribution density of (010) planes between Ag_2_Se and In‐doped Ag_2_Se, as well as the differential charge illustration. Calculated band structure of c) primitive Ag_2_Se and d) Ag_7_InSe_4_. e) Comparison of room‐temperature power factor (*S* ^2^ *σ*) and Seebeck coefficient (*S*) between this work and reported Ag_2_Se thin films.^[^ ^30^, ^31^, ^32^, ^34^, ^41^, ^42^, ^43^, ^44^, ^45^, ^46^ ^]^

Although elemental doping can be relatively easily achieved through methods such as melting,^[^ ^38^ ^]^ hydrothermal synthesis,^[^ ^39^ ^]^ or ball milling,^[^ ^40^ ^]^ achieving uniform cation‐site doping in Ag_2_Se thin films prepared via a combination of physical vapor deposition (PVD) and solution‐based methods remains challenging and has not yet been reported. To address this challenge and bridge the gap, we proposed an innovative precursor doping strategy, where In was successfully doped into a pure Ag precursor. The doped precursor then underwent a selenization reaction with a selenium‐based precursor to yield Ag_2_Se thin films doped with trace amounts of In. The mechanism is illustrated in **Figure** **1**a, and the process is detailed in Figure S1 (Supporting Information). Given that the deposited In layer is only a few nanometers thick, its deposition amount must be determined using the quartz crystal microbalance integrated into the e‐beam equipment. As shown in Figure 1b, the charge distribution on the (010) plane of In‐doped Ag_2_Se differs significantly from that of undoped Ag_2_Se. This plane is aligned with the direction of thermoelectric performance testing in the thin film. A comparison of their deformation charge densities reveals that In‐doped Ag_2_Se exhibits higher charge density, indicating that In‐doping is likely to enhance the *n* and *σ* of the thin film along its in‐plane direction. Figures S2–S5 (Supporting Information) show the band structures of pure Ag, pure In, and Ag_0.95_In_0.05_, alongside a comparison between pure Ag and Ag_0.95_In_0.05_. These results reveal a significant difference between the band structures of pure Ag and pure In, while the changes in Ag after 5% In‐doping are minimal. This limited change is consistent with the low doping concentration. Figure 1c,d compares the band structures of Ag_2_Se and Ag_7_InSe_4_, highlighting the distinct differences. For Ag_2_Se, the band structure is dominated by contributions from the conduction band, which slightly crosses the Fermi level. This characteristic grants Ag_2_Se its semi‐metallic nature with high *σ*. However, it is important to note that these calculations, performed at 0 K, may not fully represent the behavior of Ag_2_Se at finite temperatures, where it could exhibit a narrow bandgap. Conversely, the band structure of Ag_7_InSe_4_ includes contributions from both the valence band and the conduction band. The involvement of the valence band results in partial carrier annihilation, which limits the increase in *n* despite an enhancement in *σ*, thereby preventing rapid degradation of the *S*. In Figure 1e, the *S* and *S* ^2^ *σ* of the In‐doped Ag_2_Se thin films are compared with recently reported values for Ag_2_Se thin films at room temperature.^[^ ^30^, ^31^, ^32^, ^34^, ^41^, ^42^, ^43^, ^44^, ^45^, ^46^ ^]^ The results demonstrate that the degradation of *S* after In‐doping is minimal compared to other studies. Furthermore, the room‐temperature *S* ^2^ *σ* of the prepared thin films remains highly competitive. The optimal combination of *S* ^2^ *σ* and *S* enables the fabricated devices to achieve higher output voltage (*V*) and lower internal resistance (*R*_in_).

**Figure 2:**
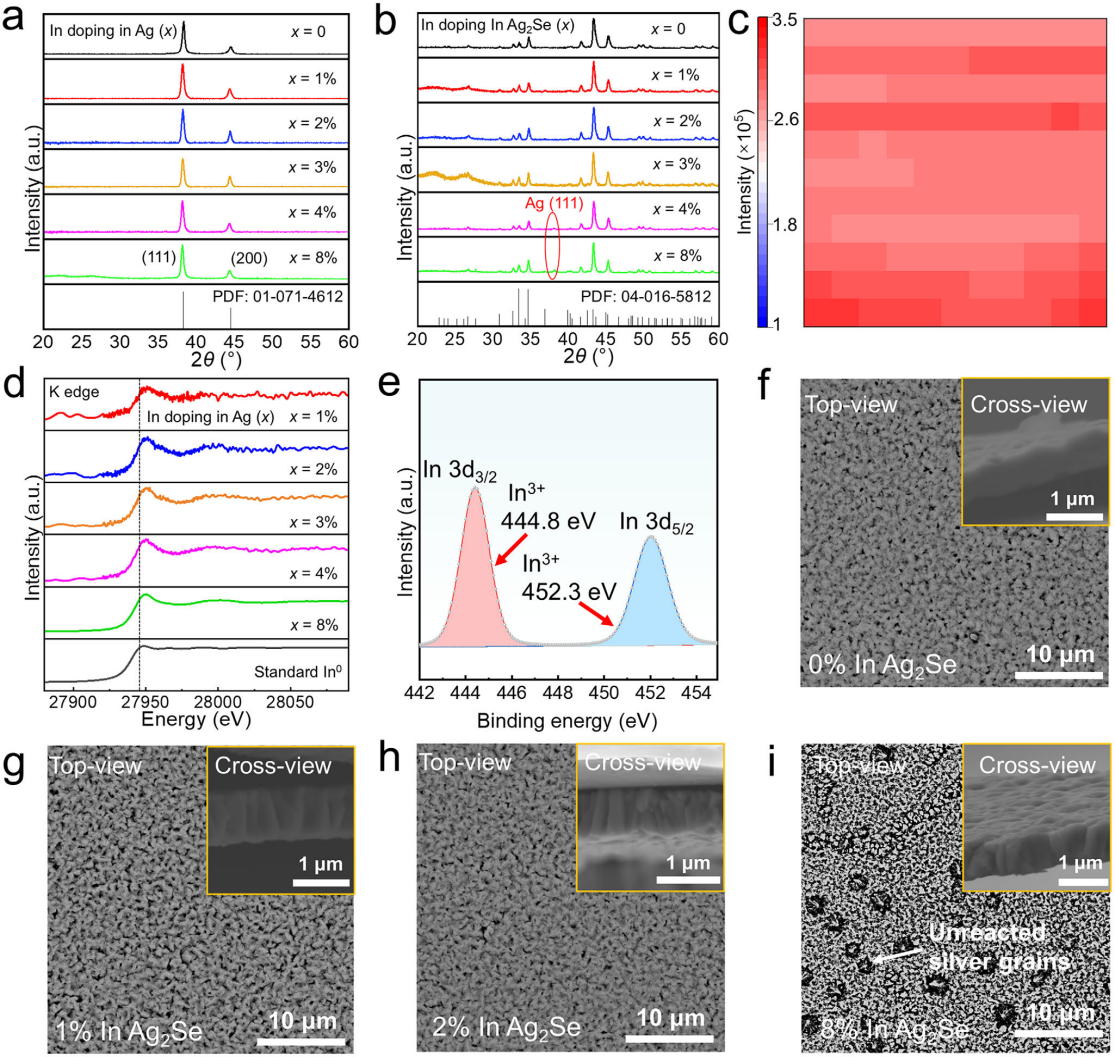
Phase, valence state, and morphology information of Ag_2_Se thin films. a) Grazing incident X‐ray diffraction (GIXRD) patterns of Ag precursors with In‐doping concentrations *x* = 0, 1, 2, 3, 4 and 8%. The 2*θ* ranges from 20° to 60°. b) GIXRD patterns of Ag_2_Se thin films with In‐doping concentrations *x* = 0, 1, 2, 3, 4 and 8%. The 2*θ* ranges from 20° to 60°. c) In‐distribution mapping based on X‐ray absorption spectroscopy (XAS) result for the *x* = 2% sample. d) XAS spectrum for the K‐edge of Ag_2_Se thin films with In‐doping concentrations *x* = 0, 1, 2, 3, 4, and 8%. e) X‐ray photoelectron spectroscopy (XPS) spectra of In 3d_3/2_ and In 3d_5/2_ for the Ag_2_Se thin film with *x* = 2%. Scanning electron microscopy (SEM) backscattering electrons (BSE) images of thin films with *x* = f) 0, g) 1%, h) 2%, and i) 8% from the top views. The insets are the corresponding section views.

To determine the optimal In‐doping concentration for the best thermoelectric performance, doping levels (*x*) of 0%, 1%, 2%, 3%, 4%, and 8% were studied. **Figure** **2**a presents the grazing incidence X‐ray diffraction (GIXRD) patterns of Ag precursors with varying *x*. All diffraction peaks between 20° and 60° match well with the powder diffraction file (PDF) of Ag (#010714612) with no detectable impurity peaks. A gradual shift of diffraction peaks to lower angles with increasing In content (Figure S6, Supporting Information) indicates lattice expansion due to partial substitution of Ag (atomic radius 144 pm) by In (155 pm), confirming effective doping. Post‐ selenization, the GIXRD patterns of Ag_2_Se films (Figure 2b) shows align with the Ag_2_Se PDF, showing anisotropy. For *x* ≥ 2%, an Ag (111) peak appears, likely due to incomplete reaction between highly doped Ag and the Se precursor, as increased In content raises the activation energy for Ag_2_Se formation. This peak becomes prominent at *x* = 8% (Figure S7, Supporting Information). A leftward shift of patterns for *x* ≥ 2% indicates excess In hinders full reaction of Ag atoms with Se, while a rightward shift (Figure S8, Supporting Information) results from Se vacancies causing lattice contraction and incorporation of smaller In^3+^ ions (76 pm) in place of Ag^+^ (126 pm). Refined GIXRD results (**Tables** **1** and **2**) confirm consistent trends in doping effects for both precursors and Ag_2_Se films. Incomplete incorporation of In during low‐temperature pre‐treatment aligns with observed discrepancies in atomic ratios. However, post‐selenization and thermal treatment fully integrate In into Ag_2_Se, primarily substituting the Ag_1_ sites while the Ag_2_ sites remain unoccupied, as illustrated in Figure 1a, in which shows the Ag_1_ and In atomic positions

**Table 1:** The refinement results of the grazing incidence X‐ray diffraction (GIXRD) for In‐doped Ag precursor.

Sample	Grain size [nm]	Micro‐strain	Lattice parameter [Å]	Atomic occupation [Ag]	Atomic occupation [In]
*x* = 0%	37.67	0.073%	4.0838	100%	0%
*x* = 1%	40.70	0.045%	4.0846	99.0%	1.0%
*x* = 2%	39.80	0.011%	4.0845	98.8%	1.2%
*x* = 3%	53.72	0.010%	4.0853	98.5%	1.5%
*x* = 4%	58.60	0.020%	4.0862	98.0%	2.0%
*x* = 8%	51.00	0.012%	4.0835	96.0%	4.0%

**Table 2:** The refinement results of GIXRD for In‐doped Ag_2_Se.

Sample	Grain size [nm]	Micro‐strain	Lattice parameter *a* [Å]	Lattice parameter *b* [Å]	Lattice parameter *c* [Å]	Atomic occupation [Ag_1_]	Atomic occupation [Ag_2_]	Atomic occupation [Se]	Atomic occupation [In_1_]	Atomic occupation [In_2_]
*x* = 0%	127.324	0.038%	4.3355	7.0671	7.779	100%	100%	100%	0%	0%
*x* = 1%	127.33	0.014%	4.3354	7.0691	7.778	99%	100%	100%	1%	0%
*x* = 2%	127.27	0.038%	4.3339	7.0663	7.774	98%	100%	100%	2%	0%
*x* = 3%	135.44	0.030%	4.3340	7.0669	7.779	97%	100%	100%	3%	0%
*x* = 4%	159.16	0.060%	4.3281	7.0659	7.783	96%	100%	100%	4%	0%
*x* = 8%	187.90	0.042%	4.3339	7.0695	7.7768	92%	100%	100%	8%	0%

Figure 2c shows the X‐ray absorption spectroscopy (XAS) map for Ag_2_Se samples at an In‐doping concentration of *x* = 2%, confirming the uniform distribution of In across the entire sample (1.5 cm × 1.5 cm). This result demonstrates the effectiveness of the precursor doping method. Figure 2d presents the K‐edge XAS spectra of Ag_2_Se samples with varying In‐doping concentrations (*x* = 0, 1, 2, 3, 4, and 8%). As the *x* increases, the spectra exhibit improved signal‐to‐noise ratios and clarity, indicating the controllability of In deposition content. The characteristic peak positions of standard In^0^ are distinct from those of oxidized In, further verifying the integration of In into the Ag_2_Se matrix. Typically, an increase in the oxidation state of an absorbing site corresponds to a rise in the absorption edge energy. However, at higher doping levels (*x* = 4% and 8%), the peak shift diminishes compared to lower doping levels. This behavior is attributed to the formation of an In‐Ag alloy, which inhibits some Ag atoms from reacting with the Se precursor, leaving trace amounts of Ag^0^ and In^0^ in the sample. Figure 2e presents the X‐ray photoelectron spectroscopy (XPS) spectra of In‐doped Ag_2_Se with *x* = 2%, revealing that In predominantly exists in the +3 oxidation state, consistent with previous studies.^[^ ^47^ ^]^ The combined results from XRD, XAS, and XPS confirm the successful doping of In into the Ag_2_Se matrix, introducing additional charge carriers to the system. Furthermore, the oxidation state of In^3+^ induces localized lattice contraction or distortion in Ag_2_Se, consistent with the rightward shift observed in the XRD diffraction patterns.

The morphology of the as‐fabricated thin films was analyzed using scanning electron microscopy (SEM) backscattered electron (BSE) imaging, as shown in Figure 2f–i and Figures S9 and S10 (Supporting Information) for In‐doped Ag_2_Se thin films with *x* = 0–8%. For *x* < 4%, the BSE images show uniform distributions with no observed precipitates. However, at *x* ≥ 4%, unreacted Ag particles emerge as precipitates, as observed in Figure 2i and Figure S10 (Supporting Information). These precipitates likely result from excessive In substitution in the Ag lattice, which raises the activation energy and prevents complete reaction with the Se precursor. Additional SEM‐BSE images and corresponding energy‐dispersive X‐ray spectroscopy (EDS) results of Ag_2_Se thin films with *x* = 0–8% are presented in Figures S11–S16 (Supporting Information) further supporting these observations. Moreover, the SEM‐BSE images of Ag precursors with In concentrations ranging from *x* = 0–8% (Figure S17, Supporting Information) reveal relatively uniform morphologies for x = 0%, 1%, 2%, and 3%. In contrast, noticeable morphological changes occur for precursors with x > 4%, likely influenced by the increased In content.

**Figure 3:**
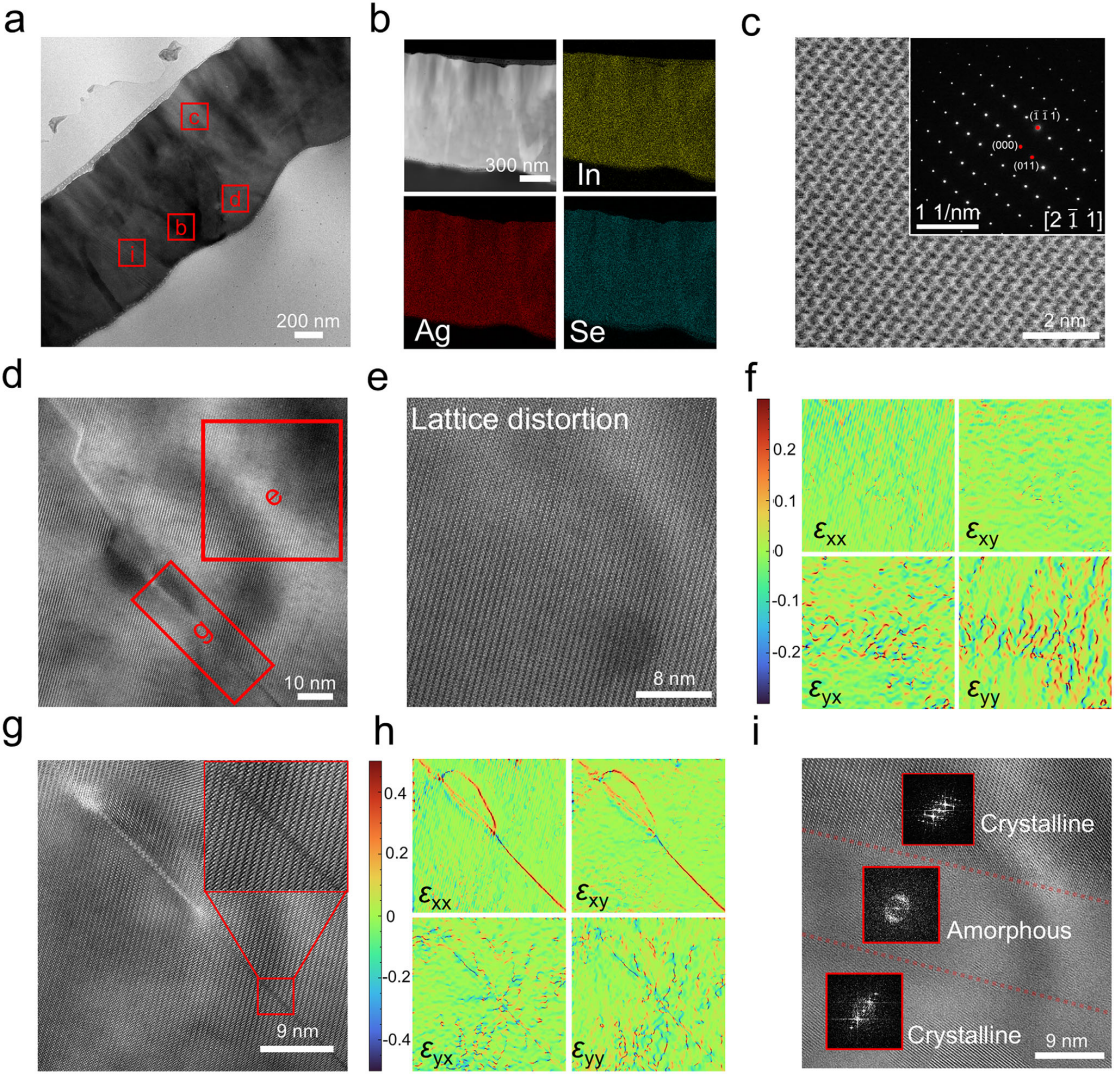
Micro/nanostructural characterizations for In‐doped Ag_2_Se thin film (*x* = 2%). a) Transmission electron microscopy (TEM) image of the specimen fabricated by focused ion beam (FIB) technique. b) Low‐magnification high‐angle annular dark‐field (HAADF) image and its corresponding dispersive X‐ray spectroscopy (EDS) maps from the selected area of (a). c) High‐resolution TEM (HRTEM) image with invisible defect area derived from (a). The inset is the selected area (electron) diffraction (SAED) image with the view zone axis of [2$\bar{1}1$]. d) HRTEM image of the potential defect‐rich area originated from the selected region of (a). e) HRTEM image of potential lattice defect area derived from the selected region of (d) and f) corresponding strain maps. g) HRTEM image of potential continuous slip defects area originated from the selected region of (d) and h) corresponding strain maps. i) HRTEM image showing the alternating crystalline‐amorphous‐crystalline structure.

To investigate the impact of In‐doping on the micro/nanostructure of Ag_2_Se thin films, a sliced sample of the In‐doped Ag_2_Se thin film (*x* = 2%) was prepared using focused ion beam (FIB) technology. High‐resolution transmission electron microscopy (HRTEM) and aberration‐corrected scanning transmission electron microscopy (Cs‐STEM) were employed for characterization. **Figure** **3**a presents a low‐magnification TEM image of the sample, revealing a dense microstructure at the micrometer scale with no visible pores. Contrast variations in the image suggest the presence of defect structures within the thin film. Figure 3b shows a low‐magnification high‐angle annular dark field (HAADF) image of a selected area in Figure 3a, along with corresponding EDS elemental mapping. The uniform distribution of In, Ag, and Se across the selected area confirms the homogeneity of doping. To validate the composition of the sample, selected composition calibration points are shown in Figure S18 (Supporting Information), with the corresponding ratios provided in Tables S1 and S2 (Supporting Information). Figure 3c displays an HRTEM image of a selected region in Figure 3a, showing no obvious defects or lattice mismatches at the nanoscale. Additionally, the strain map in Figure S19 (Supporting Information) indicates minimal strain within the crystal lattice, confirming an ordered lattice arrangement in the analyzed region. Additionally, the inset of Figure 3c displays the selected area electron diffraction (SAED) pattern along the [2$\bar{1}1$] zone axis. Although defects are minimal at small scales, Figure 3d shows a high‐intensity defect region observed in the HRTEM image. A magnified view of a selected area from Figure 3d is presented in Figure 3e where significant contrast variations suggest the accumulation of lattice distortions. The corresponding strain map in Figure 3f (related to Figure 3e) reveals strain concentration along the *y*‐axis. These distortions are likely induced by the inherent Se vacancies (V_Se_) or the substitution of In^3+^ ions within in Ag_2_Se lattice, consistent with the rightward shift observed in the XRD pattern. Figure 3g depicts another selected area from Figure 3d, showing potential continuous defects caused by lattice slip. In crystalline materials, such extended and continuous defects are effective in scattering medium‐ and short‐wavelength phonons, thereby modulating the *κ*_l_. To confirm that these slip defects are not grain boundaries between two Ag_2_Se crystals, Figure S20 (Supporting Information) provides a detailed view of a selected region from Figure 3g, and its fast Fourier transform (FFT) patterns at the slip defect. The presence of a single lattice pattern confirms that the defect resides within the crystal structure. The strain map corresponding to Figure 3g, shown in Figure 3h, indicates that the strain along the slip defect is primarily associated with *x*‐direction stress, while the surrounding strain is aligned with *y*‐direction stress. In addition to lattice distortions and slip defects, larger‐scale defects were identified, specifically alternating crystal‐amorphous‐crystal structures, as illustrated in Figure 3i. HRTEM and the corresponding FFT images reveal a clear amorphous region sandwiched between two crystalline regions with different zone axes. The amorphous region effectively scatters long‐wavelength phonons, while the phase interfaces play a positive role in reducing *κ*. A magnified view of Figure 3i is provided in Figure S21 (Supporting Information).

**Figure 4:**
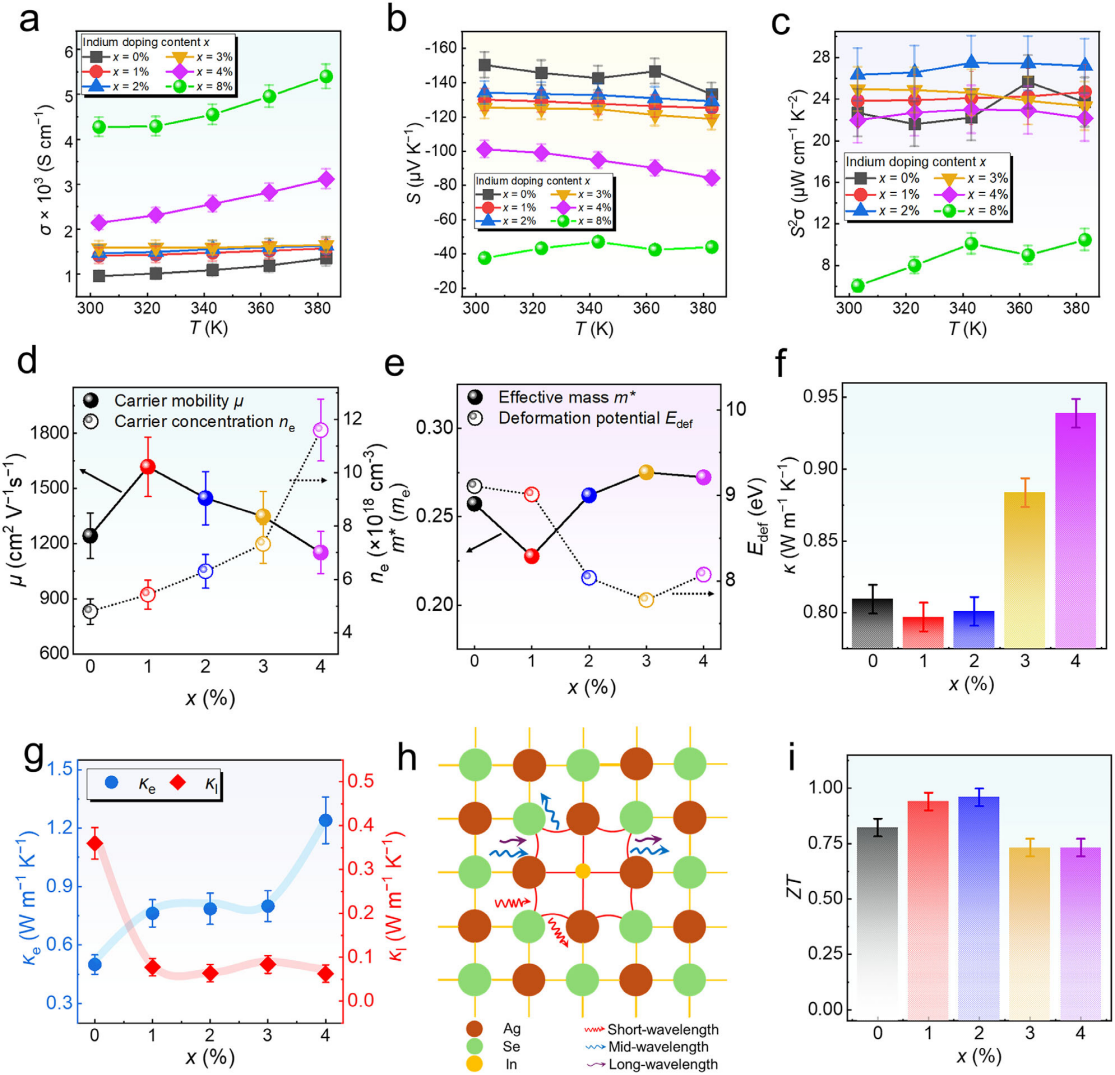
Thermoelectric performance of Ag_2_Se thin films at different In‐doped concentrations (*x* = 0%, 1%, 2%, 3%, 4% 8%). Temperature‐dependent a) electric conductivity (*σ*), b) *S*, and c) *S* ^2^ *σ*. d) Room‐temperature carrier concentration (*n*) and carrier mobility (*μ*). e) Room‐temperature deformation potential (*E*_def_) and effective mass (m*). f) *x‐*dependent room‐temperature thermal conductivity (*κ*). g) *x*‐dependent room‐temperature lattice thermal conductivity (*κ*_l_) and electron thermal conductivity (*κ*_e_). h) Schematic of phonon scattering induced by lattice distortion due to In‐doping. i) Determined room‐temperature *ZT*.

To clarify the effect of In‐doping concentration on the overall thermoelectric performance of Ag_2_Se thin films, we measured the physical properties of films with *x* ranging from 0% to 8%. **Figure** **4**a–c presents the temperature‐dependent results of *σ*, *S*, and *S* ^2^ *σ*. Overall, the *σ* increases with increasing *x*. Meanwhile, *S* decreases with increasing *x*, and the rate of decrease becomes more pronounced as In‐content *x* reaches ≥ 4%. As a result, for *S* ^2^ *σ*, a competitive room‐temperature value of 26.3 µW cm^−1^ K^−2^ is observed at *x* = 2%. The reproducibility of the sample performance is shown in Figure S22 (Supporting Information), indicating strong thermal stability and data consistency for our samples. Additionally, durability and repeating cycling test results can be seen in Figure S23 (Supporting Information).

To gain deeper insight into the variations in *σ* and *S*, we examined the relationship between *μ* and *n* in Ag_2_Se thin films as a function of *x* at room temperature, as shown in Figure 4d. The samples with *x* = 1% and 2% exhibit high *μ* exceeding 1500 cm^2^ V^−1^ s^−1^, indicating that In‐doping enhances *μ* by introducing additional charge carriers compared to the undoped sample (*x* = 0). For the sample with *x* = 4%, SEM images reveal that starting from *x* ≥ 4%, unreacted Ag grains appear due to the excessive Ag precursor failing to react with the Se precursor. These In‐doped Ag clusters exist within the matrix, leading to a subsequent increase in *μ*. In terms of *n*, it shows a positive correlation with increasing *x*, attributed to the additional electrons provided by In‐doping. The results for the *x* = 8% sample can be seen in Figure S24 (Supporting Information). Figure 4e presents the effective mass (*m* ^*^), calculated using the single parabolic band (SPB) model, and the deformation potential (*E*_def_) for samples with different In‐doping levels to further understand the reasons for changes in *n* and *μ*. It is observed that as In‐doping levels increase from *x* = 0 to *x* = 4%, the change in *m* ^*^ is insignificant, likely because the relative change in *n* remains small. However, despite the minimal variation in *m* ^*^, the degradation of *S* caused by In‐doping may arise from impurity scattering, dislocations, and other defects introduced by doping. These defects cause carrier scattering with varying intensities at different energy levels, reducing the average mean free path of carriers and thus altering *μ*. This result aligns with Figure 4d, showing that even with a relatively constant *m* ^*^, *S* can still change. Additionally, as previously mentioned, when In is introduced into the Ag_2_Se matrix, the valence band can exceed the Fermi level, indicating that bipolar effects should also be considered. Although the change in *m* ^*^ is minimal, the hole contribution induced by doping may further degrade *S*. Moreover, the sample with *x* = 2% exhibits a relatively low *E*_def_, suggesting a higher lattice deformation capacity and better ductility, which is beneficial for achieving high flexibility in the thin‐film form. However, it is noteworthy that the differences in *E*_def_ across all samples remain within 20%, indicating that other conditions or measurement errors introduced during actual testing might need to be considered when discussing specific ductility. The result of *E*_def_ and *m* ^*^ for the sample with *x* = 8% can be seen in Figure S25 (Supporting Information). Figure 4f,g shows the thermal diffusivity (*D*) data obtained using the photothermal intensity technique (PIT) with an alternating current (AC) method, which was used to determine the *κ*. The testing principle is illustrated in Figure S26 (Supporting Information), and the raw results are provided in Figures S27–S32 (Supporting Information). Porosity calibration is shown in Figure S33 (Supporting Information), while the Lorenz parameter (*L*) calibration is presented in Figure S34 (Supporting Information). Overall, the thin films exhibit relatively low *κ*, particularly for samples with In‐doping levels *x* < 3%. This can be attributed to the contribution of low film density (*ρ*). Additionally, the *κ*_l_ benefits from the introduction of amorphous structures, lattice distortions, and continuous 2D defects caused by In‐doping, as observed in the TEM analysis (Figure 3h). The reduction in *κ*_l_ helps balance the increased *κ*_e_ resulted from high *σ*, thereby optimizing the overall thermal performance of the films. The thermal performance of the *x* = 8% film is shown in Figure S35 (Supporting Information). As illustrated in Figure 4i, the optimal room‐temperature *ZT* value reaches 0.95 (±0.1), which is highly competitive compared to report values.^[^ ^30^, ^31^, ^45^, ^48^, ^49^, ^50^, ^51^, ^52^, ^53^, ^54^, ^55^, ^56^, ^57^, ^58^ ^]^

**Figure 5:**
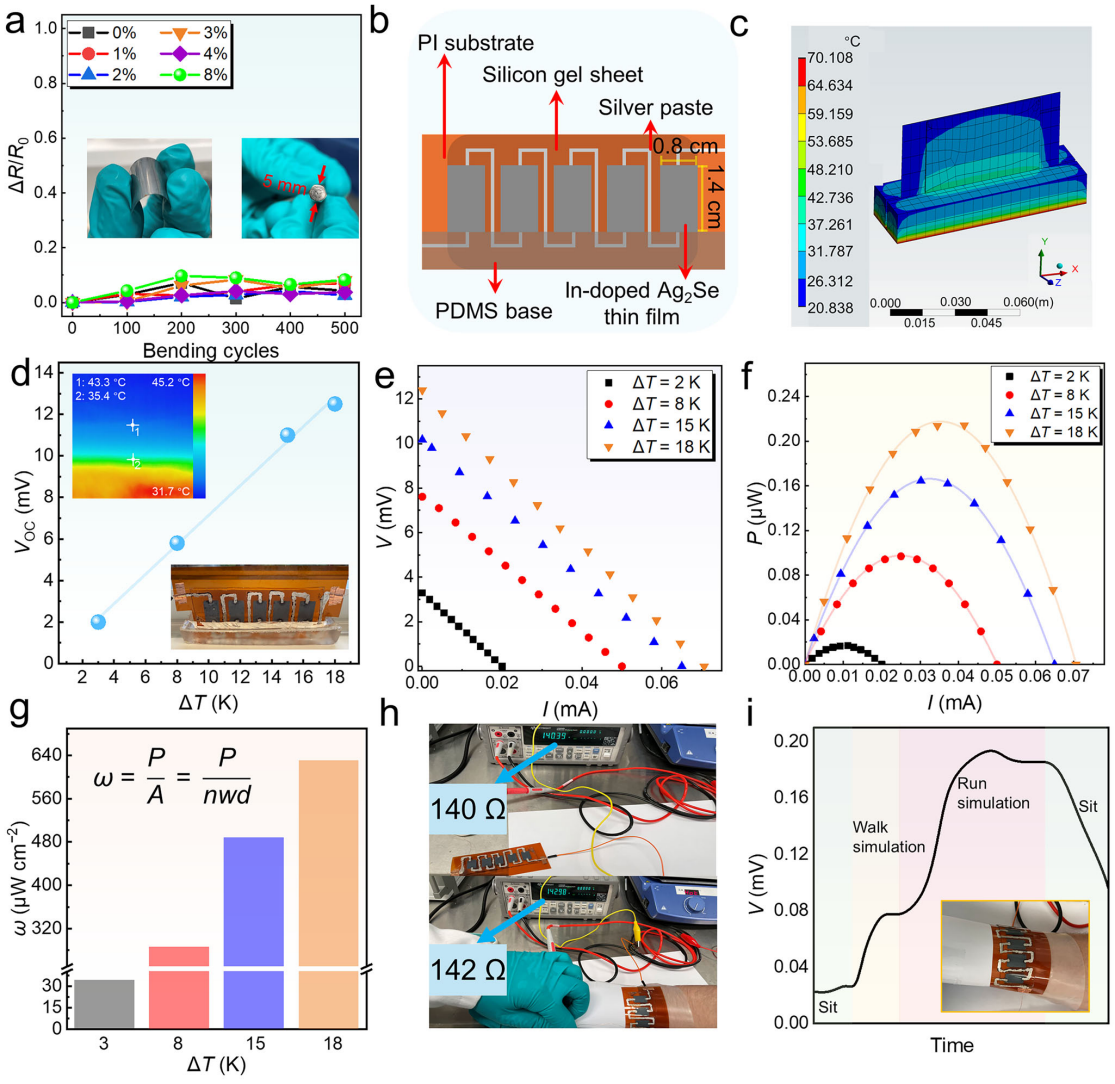
Flexibility of In‐doped Ag_2_Se thin films and the as‐fabricated device. a) Normalized resistance change (Δ*R*/*R*_0_) of the thin film with different In‐doping concentrations as a function of the bending cycles under bending radius of 5 mm. The insets are photos of the tested films and the tools used. b) Schematic for the structure and dimensions of the slotted thermoelectric device fabricated from In‐doped Ag_2_Se thin films (*x* = 2%). Here PDMS is the abbreviation of polydimethylsiloxane. c) Heat conduction simulation of the fabricated device on the heat source, modeled using ANSYS software. d) Δ*T*‐dependent open‐circuit voltage (*V*_oc_) of the as‐fabricated slotted device. e) Output voltage (*V*) and f) output power (*P*) as a function of current (*I*) as well as g) corresponding power density (*ω*) as the function of Δ*T*. h) Photos of the internal resistance *R*_in_ of the as‐fabricated device before and after wearing. i) Time‐dependent *V* of the as‐fabricated device under different motion states.

To evaluate the practical potential of the thin films, we conducted flexibility tests. **Figure** **5**a shows the normalized resistance change (Δ*R*/*R*_0_) of Ag_2_Se thin films with different In‐doping levels after 500 bending cycles at a bending radius of 5 mm. It can be observed that the Δ*R*/*R*_0_ changes by <10% for films with different *x* values, reflecting good flexibility of the thin films. This good flexibility is attributed, on the one hand, to precise thickness control during the preparation process (thinner films exhibit better flexibility), and on the other hand, to the high flexibility of the polyimide (PI) substrate. The inset of Figure 5a shows photographs of the thin film during testing and the corresponding tools. The good flexibility can be further attributed to the good contact between the film and the substrate, as well as the uniform film thickness.

To explore the potential practical applications of these thin films, we fabricated a thermoelectric device with a slotted structure (Figure 5b), where the dimensions and components are detailed. Figure 5c presents the thermal conduction simulation image based on ANSYS modeling, which demonstrates that the slotted structure enables efficient heat transfer from the substrate to the thin film, generating an effective Δ*T*. Figure 5d shows the open‐circuit voltage (*V*_oc_) of the device as a function of Δ*T*, where *V*_oc_ reaches 12.5 µV at Δ*T* = 18 K. The inset displays the infrared image of the thin film surface during testing, illustrating the Δ*T*, as well as a photograph of the actual device. Figure 5e,f presents the output voltage (*V*) versus current (*I*) and the *P* versus I, respectively. Both relationships exhibit strong consistency under different Δ*T* values, with the maximum *P* = 0.21 µW at Δ*T* = 18 K. Figure 5g shows the *ω* as a function of the test temperature. It can be observed that at Δ*T* = 18 K, the *ω* reaches ≈630 µW cm^−2^, corresponding to a *ω*_n_ = 1.95 µW cm^−2^ K^−2^. Figure 5h demonstrates the device stability during wear, where the *R*_in_ remains nearly unchanged before and after wearing, highlighting the potential for practical applications. Figure 5i displays the *V* of the thin film during different motion states when worn. Noticeable variations in *V* are observed. The inset of Figure 5i shows a photograph of the wearable device during the testing process. These results confirm that our as‐designed thin films and devices are valuable for practical applications.

## 3 Conclusion

In this work, we fabricated In‐doped Ag_2_Se thin films by doping In into the Ag precursor, followed by preheating treatment and solution‐based selenization. The substitution of In at cation sites effectively introduced additional electron carriers into the system, significantly enhancing the overall *σ* of the thin films. Furthermore, In doping generated additional micro/nanoscale defects that effectively scattered phonons, reducing the *κ*_l_. At an optimal doping concentration of 2%, the thin films achieved a competitive room‐temperature *S* ^2^ *σ* of 26.3 µW cm^−1^ K^−2^ and a *ZT* value of ≈1. The films also exhibited excellent flexibility and mechanical stability, with the change in Δ*R*/*R*_0_ remaining within 10% after multiple bending cycles. To demonstrate the practical potential of thin films, we fabricated a slotted thermoelectric device. Under a Δ*T* of 18 K, the device exhibited a *V*_oc_ of 12.5 mV, a *P* of 0.21 µW, a *ω* of 630 µW cm^−2^, and a *ω*_n_ of 1.95 µW cm^−2^ K^−2^. This study is the first to demonstrate the feasibility of cation‐site doping in Ag_2_Se, resulting in materials with competitive thermoelectric performance and excellent flexibility. These findings highlight the promising potential of In‐doped Ag_2_Se thin films for practical applications in flexible thermoelectric devices.

## 4 Experimental Section

Experimental Details are provided in the Supporting Information.

## Conflict of Interest

The authors declare no conflict of interest.

## Supporting information



Supporting Information

## Data Availability

The data that support the findings of this study are available from the corresponding author upon reasonable request.

## References

[1] W. Chen , X.‐L. Shi , M. Li , T. Liu , Y. Mao , Q. Liu , M. Dargusch , J. Zou , G. Q. M. Lu , Z.‐G. Chen , Science 2024, 386, 1265. 39666792 10.1126/science.ads5868

[2] P. Zhao , W. Xue , Y. Zhang , S. Zhi , X. Ma , J. Qiu , T. Zhang , S. Ye , H. Mu , J. Cheng , X. Wang , S. Hou , L. Zhao , G. Xie , F. Cao , X. Liu , J. Mao , Y. Fu , Y. Wang , Q. Zhang , Nature 2024, 631, 777. 38987600 10.1038/s41586-024-07621-8

[3] X.‐L. Shi , L. Wang , W. Lyu , T. Cao , W. Chen , B. Hu , Z.‐G. Chen , Chem. Soc. Rev. 2024, 53, 9254. 39143899 10.1039/d4cs00361f

[4] Q. Yang , S. Yang , P. Qiu , L. Peng , T.‐R. Wei , Z. Zhang , X. Shi , L. Chen , Science 2022, 377, 854. 35981042 10.1126/science.abq0682

[5] B. Qin , M. G. Kanatzidis , L.‐D. Zhao , Science 2024, 386, eadp2444. 39418358 10.1126/science.adp2444

[6] B. Jiang , Y. Yu , J. Cui , X. Liu , L. Xie , J. Liao , Q. Zhang , Y. Huang , S. Ning , B. Jia , B. Zhu , S. Bai , L. Chen , J. P Stephen , J. He , Science 2021, 371, 830. 33602853 10.1126/science.abe1292

[7] Y. Pei , X. Shi , A. LaLonde , H. Wang , L. Chen , G. J. Snyder , Nature 2011, 473, 66. 21544143 10.1038/nature09996

[8] S. Chen , Z. Ren , Mater. Today 2013, 16, 387.

[9] Y. Pei , H. Wang , G. J. Snyder , Adv. Mater. 2012, 24, 6125. 23074043 10.1002/adma.201202919

[10] L. Wang , W. Zhang , S. Y. Back , N. Kawamoto , D. H. Nguyen , T. Mori , Nat. Commun. 2024, 15, 6800. 39122724 10.1038/s41467-024-51120-3 PMC11316108

[11] B. Hu , X.‐L. Shi , T. Cao , S. Liu , M. Zhang , W. Lyu , L. Yin , T. Tesfamichael , Q. Liu , Z.‐G. Chen , Adv. Sci. 2024, 11, 2409788. 10.1002/advs.202409788 PMC11600257 39352315

[12] X.‐L. Shi , J. Zou , Z.‐G. Chen , Chem. Rev. 2020, 120, 7399. 32614171 10.1021/acs.chemrev.0c00026

[13] Q.‐Y. Liu , X.‐L. Shi , T.‐Y. Cao , W.‐Y. Chen , L. Li , Z.‐G. Chen , Prog. Mater. Sci. 2024, 150, 101420.

[14] T. Cao , X.‐L. Shi , M. Li , B. Hu , W. Chen , W.‐D. Liu , W. Lyu , J. MacLeod , Z.‐G. Chen , eScience 2023, 3, 100122.

[15] Z. Zhou , G. Han , X. Lu , G. Wang , X. Zhou , J. Magnes. Alloy. 2022, 10, 1719.

[16] T.‐R. Wei , P. Qiu , K. Zhao , X. Shi , L. Chen , Adv. Mater. 2023, 35, 2110236. 10.1002/adma.202110236 36036433

[17] N.‐H. Li , Q. Zhang , X.‐L. Shi , J. Jiang , Z.‐G. Chen , Adv. Mater. 2024, 36, 2313146.

[18] Z.‐H. Zheng , X.‐L. Shi , D.‐W. Ao , W.‐D. Liu , M. Li , L.‐Z. Kou , Y.‐X. Chen , F. Li , M. Wei , G.‐X. Liang , P. Fan , G. Q. Lu , Z.‐G. Chen , Nat. Sustain. 2023, 6, 180.

[19] Y. Lu , Y. Zhou , W. Wang , M. Hu , X. Huang , D. Mao , S. Huang , L. Xie , P. Lin , B. Jiang , B. Zhu , J. Feng , J. Shi , Q. Lou , Y. Huang , J. Yang , J. Li , G. Li , J. He , Nat. Nanotechnol. 2023, 18, 1281. 37500776 10.1038/s41565-023-01457-5

[20] T. Deng , Z. Gao , Z. Li , P. Qiu , Z. Li , X. Yuan , C. Ming , T.‐R. Wei , L. Chen , X. Shi , Science 2024, 386, 1112. 39636976 10.1126/science.adr8450

[21] N. Chen , H. Zhu , G. Li , Z. Fan , X. Zhang , J. Yang , T. Lu , Q. Liu , X. Wu , Y. Yao , Y. Shi , H. Zhao , Nat. Commun. 2023, 14, 4932. 37582957 10.1038/s41467-023-40648-5 PMC10427716

[22] Z. Liu , N. Sato , W. Gao , K. Yubuta , N. Kawamoto , M. Mitome , K. Kurashima , Y. Owada , K. Nagase , C.‐H. Lee , J. Yi , K. Tsuchiya , T. Mori , Joule 2021, 5, 1196.

[23] F. Jiang , C. Lin , J. Cheng , H. Yu , Y. Zhou , X. Ma , L. Wu , S. Ye , J. Chen , S. Zhi , Y. Xu , P. Zhao , X. Wang , F. Cao , Q. Zhang , J. Mao , Adv. Funct. Mater. 2024, 2415000.

[24] A. K. R. Ang , I. Yamazaki , K. Hirata , S. Singh , M. Matsunami , T. Takeuchi , ACS Appl. Mater. Interfaces 2023, 15, 46962. 37768216 10.1021/acsami.3c09823

[25] S. Lin , L. Guo , X. Wang , Y. Liu , Y. Wu , R. Li , H. Shao , M. Jin , J. Materiomics 2023, 9, 754.

[26] T. Kleinhanns , F. Milillo , M. Calcabrini , C. Fiedler , S. Horta , D. Balazs , M. J. Strumolo , R. Hasler , J. Llorca , M. Tkadletz , R. L. Brutchey , M. Ibáñez , Adv. Energy Mater. 2024, 14, 2400408.

[27] D. Zhang , L. Li , X. Zhang , L. Guan , S. Jin , J. Jia , J. Wen , W. Zeng , Adv. Funct. Mater. 2024, 10.1002/adfm.202419392.

[28] H. Chen , C. Shao , S. Huang , Z. Gao , H. Huang , Z. Pan , K. Zhao , P. Qiu , T.‐R. Wei , X. Shi , Adv. Energy Mater. 2024, 14, 2303473.

[29] Q. Liang , D. Yang , F. Xia , H. Bai , H. Peng , R. Yu , Y. Yan , D. He , S. Cao , G. Van Tendeloo , G. Li , Q. Zhang , X. Tang , J. Wu , Adv. Funct. Mater. 2021, 31, 2106938.

[30] Y. Lei , R. Qi , M. Chen , H. Chen , C. Xing , F. Sui , L. Gu , W. He , Y. Zhang , T. Baba , T. Baba , H. Lin , T. Mori , K. Koumoto , Y. Lin , Z. Zheng , Adv. Mater. 2021, 34, 2104786. 10.1002/adma.202104786 34837249

[31] D. Yang , X.‐L. Shi , M. Li , M. Nisar , A. Mansoor , S. Chen , Y. Chen , F. Li , H. Ma , G. X. Liang , X. Zhang , W. Liu , P. Fan , Z. Zheng , Z.‐G. Chen , Nat. Commun. 2024, 15, 923. 38296942 10.1038/s41467-024-45092-7 PMC10830499

[32] Y.‐X. Chen , X.‐L. Shi , J.‐Z. Zhang , M. Nisar , Z.‐Z. Zha , Z.‐N. Zhong , F. Li , G.‐X. Liang , J.‐T. Luo , M. Li , T. Cao , W.‐D. Liu , D.‐Y. Xu , Z.‐H. Zheng , Z.‐G. Chen , Nat. Commun. 2024, 15, 8356. 39333137 10.1038/s41467-024-52680-0 PMC11436659

[33] H. Wu , X.‐l. Shi , J. Duan , Q. Liu , Z.‐G. Chen , Energy Environ. Sci. 2023, 16, 1870.

[34] S. Y. Tee , X. Y. Tan , X. Wang , C. J. J. Lee , K. Y. Win , X. P. Ni , S. L. Teo , D. H. L. Seng , Y. Tanaka , M.‐Y. Han , Inorg. Chem. 2022, 61, 6451. 35438965 10.1021/acs.inorgchem.2c00060

[35] S. Y. Tee , D. Ponsford , C. L. Lay , X. Wang , X. Wang , D. C. J. Neo , T. Wu , W. Thitsartarn , J. C. C. Yeo , G. Guan , T.‐C. Lee , M.‐Y. Han , Adv. Sci. 2022, 9, 2204624. 10.1002/advs.202204624 PMC9799025 36285805

[36] S. Jindal , S. Singh , G. S. S. Saini , S. K. Tripathi , Mater. Res. Bull. 2022, 145, 111525.

[37] M. Wu , K. Cai , X. Li , Y. Li , Y. Liu , Y. Lu , Z. Wang , W. Zhao , P. Wei , ACS Appl. Mater. Interfaces 2022, 14, 4307. 35005880 10.1021/acsami.1c21701

[38] W.‐Y. Lyu , W.‐D. Liu , M. Li , X.‐L. Shi , M. Hong , T. Cao , K. Guo , J. Luo , J. Zou , Z.‐G. Chen , J. Mater. Sci. Technol. 2023, 151, 227.

[39] W.‐Y. Chen , X.‐L. Shi , Q. Yang , M. Li , W. Lyu , T. Liu , T. Cao , B. Hu , W. Liu , S. Sun , Y. Mao , M. Dargusch , J. Zou , Z.‐G. Chen , Chem. Eng. J. 2023, 475, 146428.

[40] Z. Liu , W. Gao , H. Oshima , K. Nagase , C.‐H. Lee , T. Mori , Nat. Commun. 2022, 13, 1120. 35236865 10.1038/s41467-022-28798-4 PMC8891317

[41] J. A. Perez‐Taborda , O. Caballero‐Calero , L. Vera‐Londono , F. Briones , M. Martin‐Gonzalez , Adv. Energy Mater. 2018, 8, 1702024.

[42] Y. Han , X. Li , Y. Jin , X. Wang , X. Sun , C. J. An , Small 2024, 20, 2309863. 10.1002/smll.202309863 38368256

[43] S. Hou , Y. Liu , Y. Luo , X. Wang , L. Yin , X. Sun , Z. Wu , J. Wang , M. Li , Z. Chen , Y. Wang , J. Sui , J. Mao , X. Liu , Q. Zhang , F. Cao , Cell Rep. Phys. Sci. 2022, 3, 101146.

[44] Y. Liu , Y. Lu , Z. Wang , J. Li , P. Wei , W. Zhao , L. Chen , K. Cai , J. Mater. Chem. A 2022, 10, 25644.

[45] C. Jiang , P. Wei , Y. Ding , K. Cai , L. Tong , Q. Gao , Y. Lu , W. Zhao , S. Chen , Nano Energy 2021, 80, 105488.

[46] Y. Liu , Q. Zhang , A. Huang , K. Zhang , S. Wan , H. Chen , Y. Fu , W. Zuo , Y. Wang , X. Cao , L. Wang , U. Lemmer , W. Jiang , Nat. Commun. 2024, 15, 2141. 38459024 10.1038/s41467-024-46183-1 PMC10923913

[47] P. K. Biswas , A. De , L. K. Dua , L. Chkoda , B. Mater. Sci. 2006, 29, 323.

[48] M. Zhang , Y. Liu , J. Li , C. Wu , Y. Liu , P. Wei , W. Zhao , K. Cai , Carbon 2024, 229, 119480.

[49] Y. Lu , X. Han , P. Wei , Y. Liu , Z. Wang , X. Zuo , W. Zhao , K. Cai , Chem. Eng. J. 2024, 485, 149793.

[50] Y. Li , Q. Lou , J. Yang , K. Cai , Y. Liu , Y. Lu , Y. Qiu , Y. Lu , Z. Wang , M. Wu , J. He , S. Shen , Adv. Funct. Mater. 2022, 32, 2106902.

[51] M. Saeidi‐Javash , K. Wang , M. Zeng , T. Luo , A. W. Dowling , Y. Zhang , Energy Environ. Sci. 2022, 15, 5093.

[52] Z.‐H. Zheng , Y.‐L. Li , J.‐Y. Niu , M. Wei , D.‐L. Zhang , Y.‐m. Zhong , M. Nisar , A. Abbas , S. Chen , F. Li , G.‐X. Liang , P. Fan , Y.‐X. Chen , J. Mater. Chem. A 2022, 10, 21603.

[53] Z.‐Y. Yang , X.‐Z. Jin , W.‐Y. Wang , C.‐H. Huang , Y.‐Z. Lei , Y. Wang , J. Mater. Chem. A 2022, 10, 21080.

[54] M. M. Mallick , A. G. Rösch , L. Franke , A. Gall , S. Ahmad , H. Geßwein , A. Mazilkin , C. Kübel , U. Lemmer , J. Mater. Chem. A 2020, 8, 16366.

[55] M. M. Mallick , L. Franke , A. G. Rösch , U. Lemmer , ACS Energy Lett. 2021, 6, 85.

[56] D. Lee , W. Park , Y. A. Kang , H. T. Lim , S. Park , Y. Mun , J. Kim , K.‐S. Jang , ACS Appl. Mater. Interfaces 2023, 15, 3047. 36599123 10.1021/acsami.2c20115

[57] Y. Ding , Y. Qiu , K. Cai , Q. Yao , S. Chen , L. Chen , J. He , Nat. Commun. 2019, 10, 841. 30783113 10.1038/s41467-019-08835-5 PMC6381183

[58] D. Palaporn , W. Mongkolthanaruk , K. Faungnawakij , K. Kurosaki , S. Pinitsoontorn , ACS Appl. Energy Mater. 2022, 5, 3489.

