# Supplementary material for: Indium‐Doping Advances High‐Performance Flexible Ag2Se Thin Films
Source: Adv Sci (Weinh). 2025 Mar 17;12(18):2500364. doi: 10.1002/advs.202500364 (PMC12079533; doi:10.1002/advs.202500364)
Supplement: Supplementary file 1 — Supporting Information [file ADVS-12-2500364-s001.docx]

Supporting Information

**Indium-Doping Advances High-Performance Flexible Ag_2_Se Thin Films**

Tianyi Cao, Xiao-Lei Shi,^*^ Boxuan Hu, Siqi Liu, Wanyu Lyu, Meng Li, Sen Wang, Wenyi Chen, Wei-Di Liu, Raza Moshwan, Tuquabo Tesfamichael, Jennifer MacLeod, and Zhi-Gang Chen^*^

T. Cao, Dr. X.-L. Shi, B. Hu, S. Liu, W. Lyu, Dr. M, Li, Dr. S. Wang, W. Chen, Dr. W.-D. Liu, Dr. R. Moshwan, Prof. J. MacLeod, Prof. Z.-G. Chen

School of Chemistry and Physics, ARC Research Hub in Zero-emission Power Generation for Carbon Neutrality, and Centre for Materials Science, Queensland University of Technology, Brisbane, Queensland 4000, Australia

E-mail: xiaolei.shi@qut.edu.au (X.-L. Shi); zhigang.chen@qut.edu.au (Z.-G. Chen)

Dr. T. Tesfamichael

School of Mechanical, Medical and Process Engineering, Queensland University of Technology, Brisbane, Queensland, 4001 Australia

**Corresponding Author**

* Xiao-Lei Shi: xiaolei.shi@qut.edu.au

* Zhi-Gang Chen: zhigang.chen@qut.edu.au

1. **Chemical reaction principles**

According to the report,^[1]^ Se can dissolve in Na_2_S·9H_2_O and follows the reaction below:

2Se+S^2−^ ↔ (Se_2_S)^2−^ (S1)

where Se combines with S to form (Se_2_S)^2−^ or (Se*_x_*S)^2−^, which is a soluble selenium sulfide. The chain structure of (Se*_x_*S)^2−^ can be described as:

(Se)*_x_*-Se-S^2−^-Se-(Se)*_x_* (S2)

where the negative valence is still provided by S^2−^. Se atoms are combined with S atoms in a polysulfide chain-like structure and dissolve in water. Moreover, Na_2_S·9H_2_O can react with H_2_O under room-temperature conditions to form HS^−^. However, Se removes sulfur from HS^−^, which suppresses the escape of S^−^ in gaseous form from the system, thereby increasing the solubility of Se. According to another report,^[2]^ the reaction of selenization for Ag may be described as:

(Se*_x_*S)^2−^ + 2Ag → Ag_2_Se + (Se*_x_*_−1_S) (S3)

We believe that the reaction can proceed until (Se*_x_*S)^2−^ (where *x* = 2), indicating that Se content of the (Se*_x_*S)^2−^ directly influences the reaction process. Therefore, we set the concentration of Se in the solution as the main variable in this research.

1. **Materials synthesis**

Pure Ag thin films were prepared on polyimide (PI) substrates using electron beam evaporation (PVD 75 E-beam, Kurt J. Lesker). A carbon crucible size (2.96 cm top × 1.43 cm height × 0.24 cm wall thickness, 15-degree wall angle) was used to hold Ag pellets (99.99% purity, Kurt J. Lesker) and In particles (99.99% purity, Kurt J. Lesker) inside the deposition chamber. The PI film (30 mm × 30 mm × 0.12 mm, CS Hyde Company) was ultrasonically cleaned in ethanol for 15 minutes before being fixed onto the deposition plate. The chamber background pressure was maintained below 5 × 10^−6^ Torr. The deposition source switching is managed through the programmed settings of the e-beam equipment. The Ag deposition parameters were set to a current (*I*) of 31 mA and a voltage (*V*) of 10 kV, with the thickness monitored and controlled to approximately 500 nm using a quartz crystal microbalance. For In deposition, the electron beam parameters were adjusted to *I* = 4~5.5 mA (corresponding to doping concentrations ranging from 1% to 8%) and *V* = 10 kV. Following electron beam deposition, the In-doped Ag thin films underwent preheating in an oven before selenization using a solution-based method. The Se precursor solution was prepared by dissolving 0.1 g of Se powder (100 mesh, 99.99%, Sigma-Aldrich) and 0.6 g of Na_2_S·9H_2_O (98%, Sigma-Aldrich) in 40 ml of deionized water. The films were immersed in the reaction solution for 2 minutes, after which they were removed, and surface residues were thoroughly cleaned with isopropanol and a small amount of deionized water. The cleaned samples were subsequently annealed in an oven (ACROSS International) at 100 °C for 10 hours to produce the final Ag_2_Se thin films for characterization and testing.

1. **Estimation of In-doping concentrations.**

The In-doping concentration can be calculated as follows, using 1% In as an example: 500 nm of Ag was deposited *via* PVD. The substrate area is set to 2 cm^2^ , and the amount of Ag corresponds to 9.72 × 10^−6^ mol. For 1% In-doping, the corresponding moles of In are 9.72 × 10^−8^ mol. Recalculating this to thickness gives 7.637 nm. Here, the bulk densities of both Ag and In are utilized. Therefore, the In-doping levels of 1%, 2%, 3%, 4%, and 8% correspond to thicknesses of approximately 8 nm, 16 nm, 24 nm, 32 nm, and 64 nm, respectively. The thickness was measured using the quartz crystal microbalance in the E-beam deposition system.

1. **Characterizations**

Grazing incidence X-ray diffraction (GIXRD) analysis was performed using a Rigaku Smart Lab instrument with CuKα radiation across an angular range of 20° to 60° in 0.02° increments to determine the phase composition of the Ag_2_Se thin-film samples. X-ray Absorption Spectroscopy (XAS) was performed at the Standard Experimental Station for Efficient XAS using a synchrotron source. X-ray Photoelectron Spectroscopy (XPS) was conducted using a Kratos Axis Supra photoelectron spectrometer. Morphological analysis, elemental mapping, and composition assessment were conducted using a JEOL 7001F scanning electron microscopy (SEM) and a HITACHI SU 7000 SEM. For energy dispersive X-ray spectroscopy (EDS) analysis, a Bruker EDS QUANTAX detector was employed. To prepare lamella samples of Ag_2_Se thin films, FEI Scios FIB focused ion beam technology was utilized. Microstructural analysis was conducted using spherical aberration-corrected scanning transmission electron microscopy (Cs-STEM) with JEOL NEOARM, performing high-angle annular dark-field (HAADF) imaging in STEM mode. High-resolution TEM imaging was performed using a JEOL 2100 transmission electron microscope.

1. **Thermoelectric performance evaluation**

The electrical conductivity (*σ*) and Seebeck coefficient (*S*) were measured using a Seebeck coefficient and electrical conductivity measurement system (ZEM-3). Thermal conductivity (*κ*) was determined *via* the alternating current method for thermal diffusivity (*D*) using the RIKO Laser-PIT system. Carrier concentration (*n*) and mobility (*μ*) were measured using a Van der Pauw Hall measurement instrument (CH-70, CH-Magnetoelectricity Technology Co., Ltd., China) under a magnetic field of up to 500 mT. The *n* and *μ* were calculated as *n* = 1/*eR* and *μ* = *σR*, respectively.

1. **Flexibility performance evaluation**

The flexibility test of the device involved using a 5 mm hexagon socket screwdriver as the bending fulcrum to conduct 500 bending cycles with a bending radius of 5 mm. Subsequently, a multimeter/DC power supply device (KEYSIGHT U3606B) was used to confirm the resistance at the same location after every 100 bending cycles. The resistance change rate was then calculated using Δ*R*/*R*_0_, where *R*_0_ represented the resistance of the test location before bending, and Δ*R* was the difference between the resistance *R* after bending and *R*_0_.

1. **Device assembling and performance evaluation**

The assembly of the device is performed in three stages. The In-doped Ag thin film is first deposited onto the polyimide (PI) substrate using electron beam (E-beam) evaporation combined with masking. This is followed by pre-heat treatment and selenization. All sections of the In-doped Ag thin film are then selenized using a solution-based method to convert them into In-doped Ag_2_Se thin films (detailed steps are provided in the material synthesis section). Silver paste is applied to assemble the conductive wires, which are then embedded into a custom-made polydimethylsiloxane (PDMS) gel base. A thermal gradient simulation is performed using a hotplate stirrer (IKA C-MAG HS7) to evaluate the thermoelectric performance. Under load application, the output performance is measured using a multimeter/DC power supply device (KEYSIGHT U3606B) alongside a standard multimeter. The standard multimeter is used to measure the load voltage, while the multimeter/DC power supply device is employed to measure the test current. Thermal flow simulation of the device was performed using ANSYS software.

1. **First-principles calculations**

Density-functional theory (DFT) calculations were performed using the all electron projected augmented wave (PAW) method, as implemented in the Vienna Ab initio Simulation Package (VASP).^[3-8]^ The generalized gradient approximation (GGA) with the fully relativistic Perdew-Burke-Ernzerhof (PBE) functional was employed to treat the exchange correlations.^[9]^ The valence wave functions were expanded in a plan-wave basis with a cut-off energy of 500 eV. All atoms were allowed to relax in their geometric optimizations until the Hellmann–Feynman force is less than 1×10^–2^ eV·Å^–1^. The convergence criterion and the Monkhorst-Pack **k**-mesh adopted for ionic relaxation are 1×10^–7^ eV per electron and 0.06π per Å, respectively. A denser 0.02π Å^–1^ Monkhorst-Pack **k**-mesh was adopted for calculating density-of-state (DOS), and a line-mode **k**-path based on Brillouin path features indicated by the AFLOW framework was adopted for calculating band structures.^[10, 11]^ To precisely predict bandgap, the Hubbard U model was considered, with the on-site coulombic (U) and the exchange (J) terms combined in a single effective U parameter of 5.8 eV for Ag_4d orbitals and 1.9 eV for In_4d orbitals, determined using the linear corresponding method.

*Modelling*. For calculation details of the single parabolic band (SPB) modeling, there are:^[11-15]^

$S\left( \eta\right)=\frac{k_{B}}{e}\cdot\left[ \frac{\left( g+\frac{5}{2} \right)\cdot F_{g+\frac{3}{2}}\left( \eta\right)}{\left( g+\frac{3}{2} \right)\cdot F_{g+\frac{1}{2}}\left( \eta\right)}-\eta\right]$ (S4)

$n=\frac{1}{e\cdot R_{H}}=\frac{{(2m^{*}\cdot k_{B}T)}^{\frac{3}{2}}}{3\pi^{2}\hbar^{3}}\cdot\frac{\left( g+\frac{3}{2} \right)^{2}\cdot F_{g+\frac{1}{2}}^{2}(\eta)}{(2g+\frac{3}{2})\cdot F_{2g+\frac{1}{2}}(\eta)}$ (S5)

$\mu=\left[ \frac{e\pi\hbar^{4}}{\sqrt{2}{(k_{B}T)}^{\frac{3}{2}}}\frac{C_{l}}{{E_{\mathrm{def}}}^{2}{(m^{*})}^{\frac{5}{2}}} \right]\frac{(2g+\frac{3}{2})\cdot F_{2g+\frac{1}{2}}(\eta)}{\left( g+\frac{3}{2} \right)^{2}\cdot F_{g+\frac{1}{2}}(\eta)}$ (S6)

$L={(\frac{k_{B}}{e})}^{2}\cdot\left\{ \frac{\left( g+\frac{7}{2} \right)\cdot F_{g+\frac{5}{2}}\left( \eta\right)}{\left( g+\frac{3}{2} \right)\cdot F_{g+\frac{1}{2}}\left( \eta\right)}-\left[ \frac{\left( g+\frac{5}{2} \right)\cdot F_{g+\frac{3}{2}}\left( \eta\right)}{\left( g+\frac{3}{2} \right)\cdot F_{g+\frac{1}{2}}\left( \eta\right)} \right]^{2} \right\}$ (S7)

where *η* is the reduced Fermi level, *k*_B_ is the Boltzmann constant, *g* is the carrier scattering factor (*g* = −1/2 for acoustic phonon scattering), *ħ* is the reduced plank constant, *C*_l_ is the elastic constant for longitudinal vibrations, *E*_def_ is the deformation potential coefficient, *m** is the effective mass, and *L* is the Lorenz number. For *C*_l_, there is:^[12-15]^

*C*_l_ = *v*_l_^2^⋅*ρ* (S8)

where *v*_l_ is the longitudinal sound velocity. *F_i_*(*η*) is the Fermi integral expressed as:^[12-15]^

$F_{i}\left( \eta\right)=\int_{0}^{\infty} \frac{x^{i}}{1+e^{(x-\eta)}}dx$ (S9)

1. **Supplementary figures**


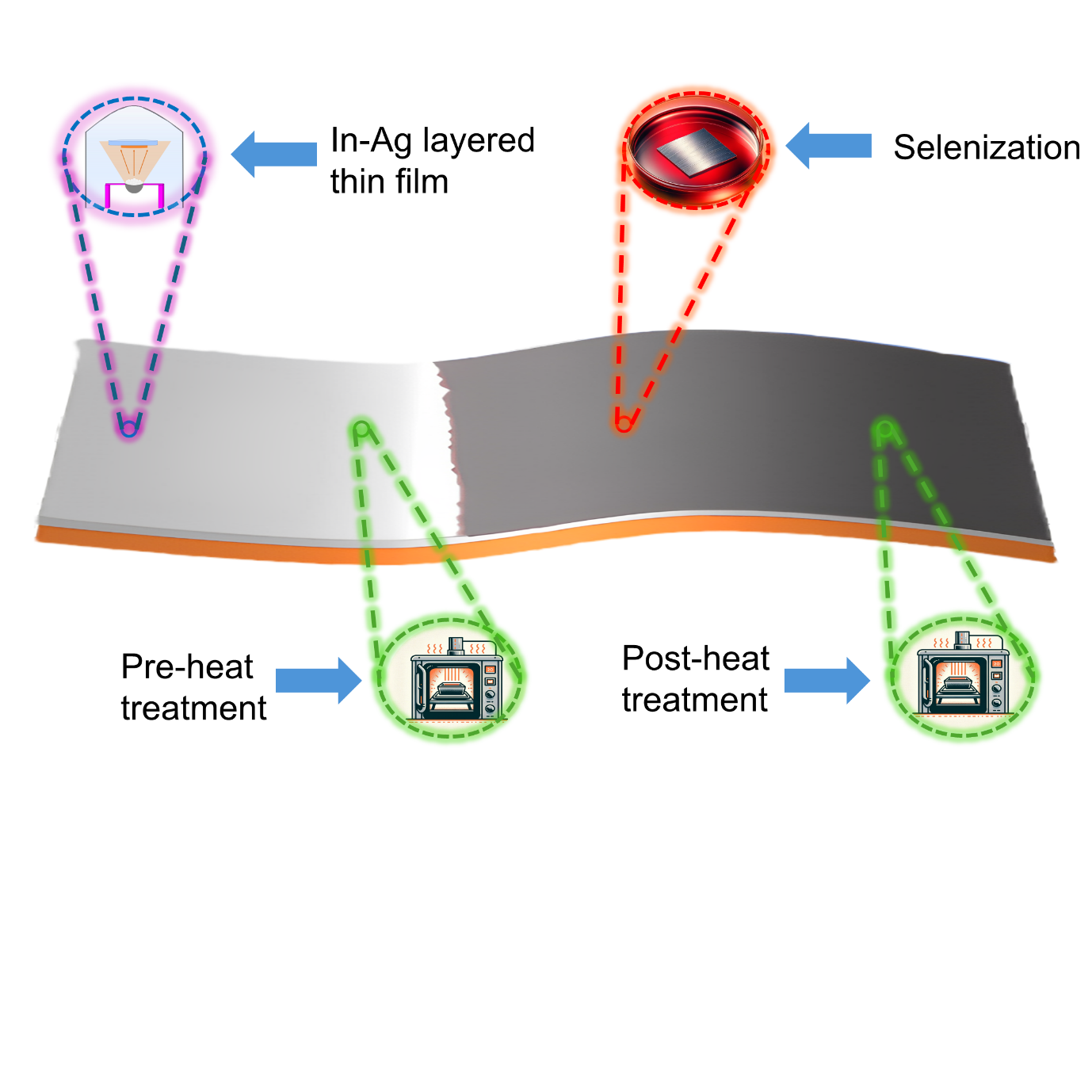


**Figure S1**. Schematic diagram of the preparation of In-doped Ag_2_Se thin films.


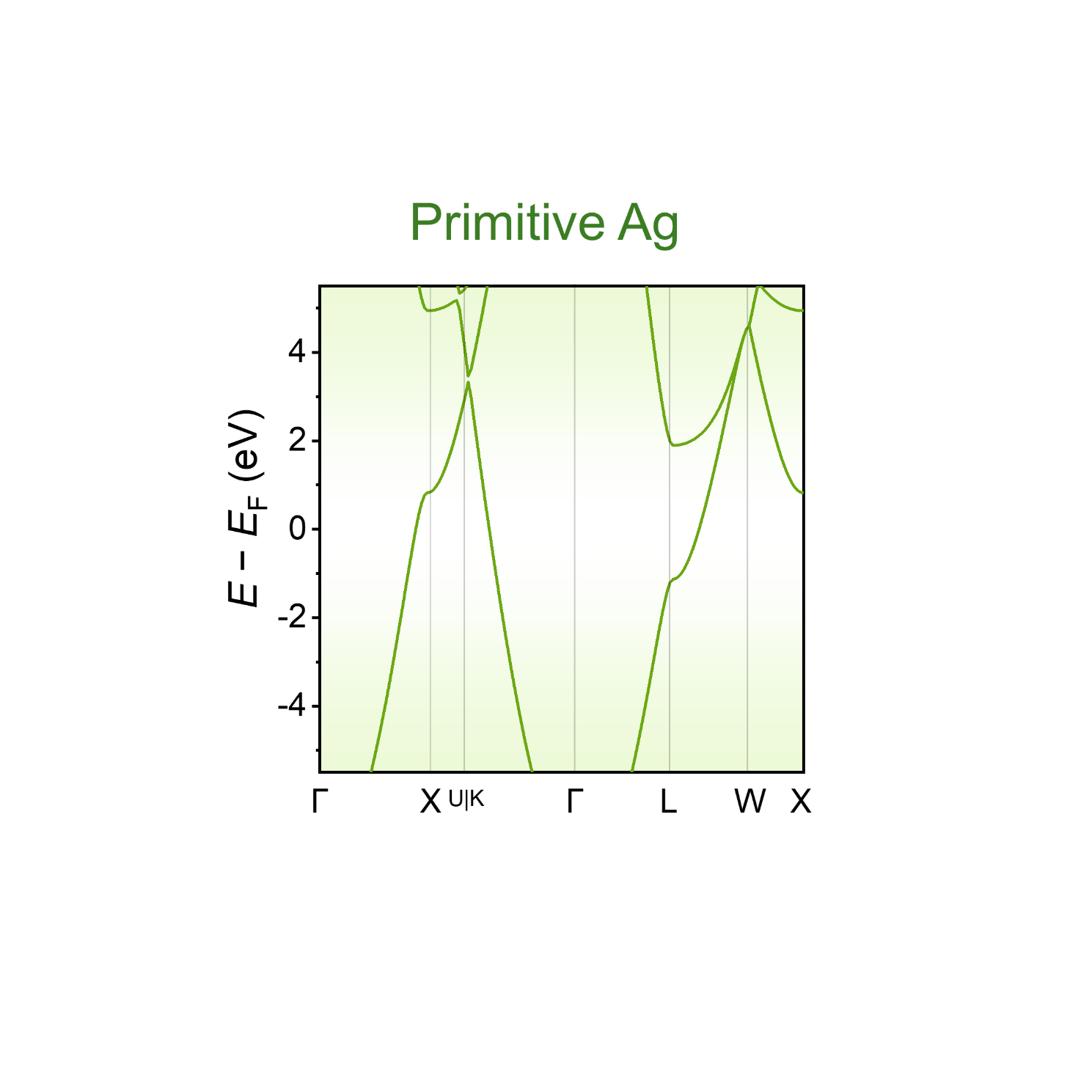


**Figure S2**. Calculated energy band structure for primitive Ag.


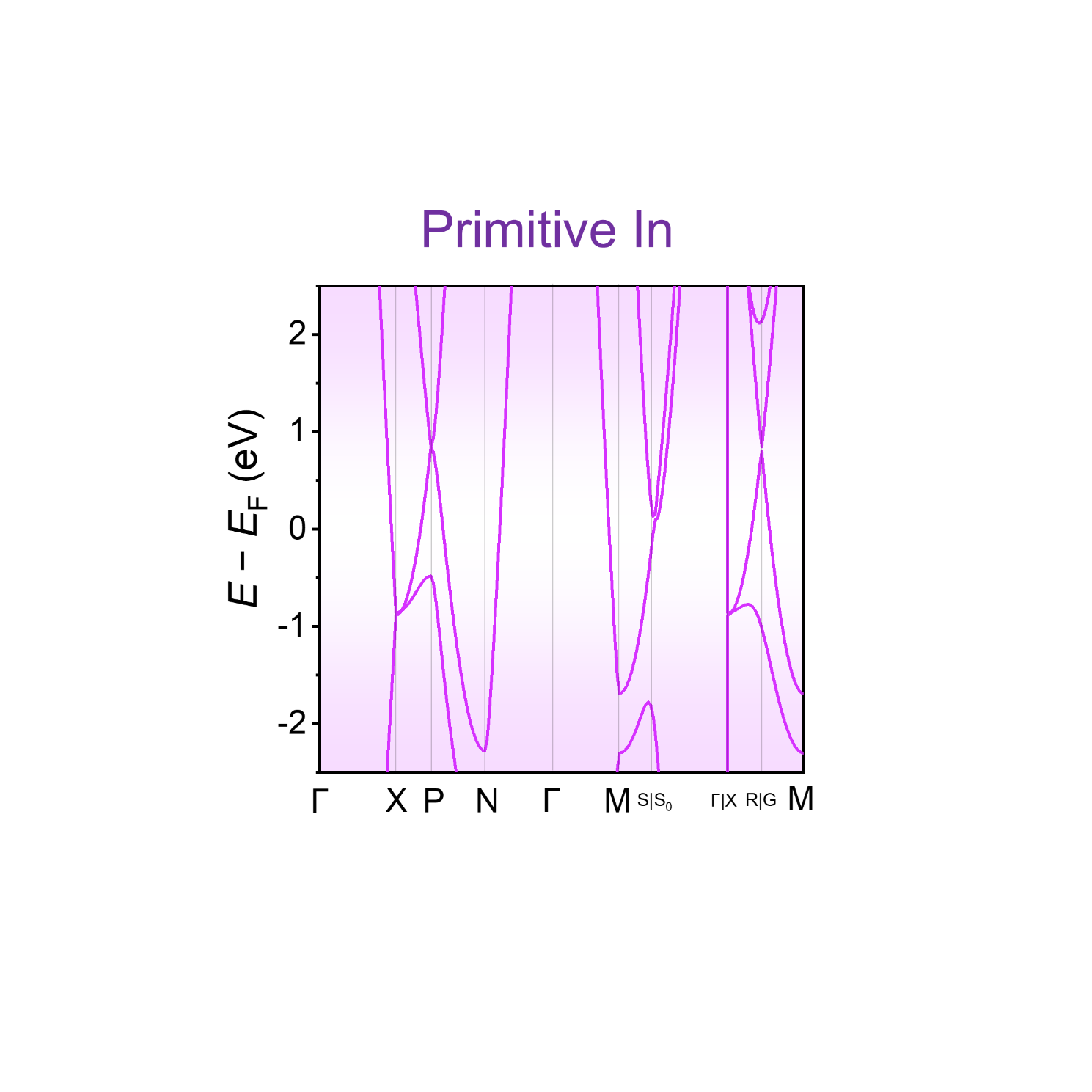


**Figure S3**. Calculated energy band structure for primitive In.


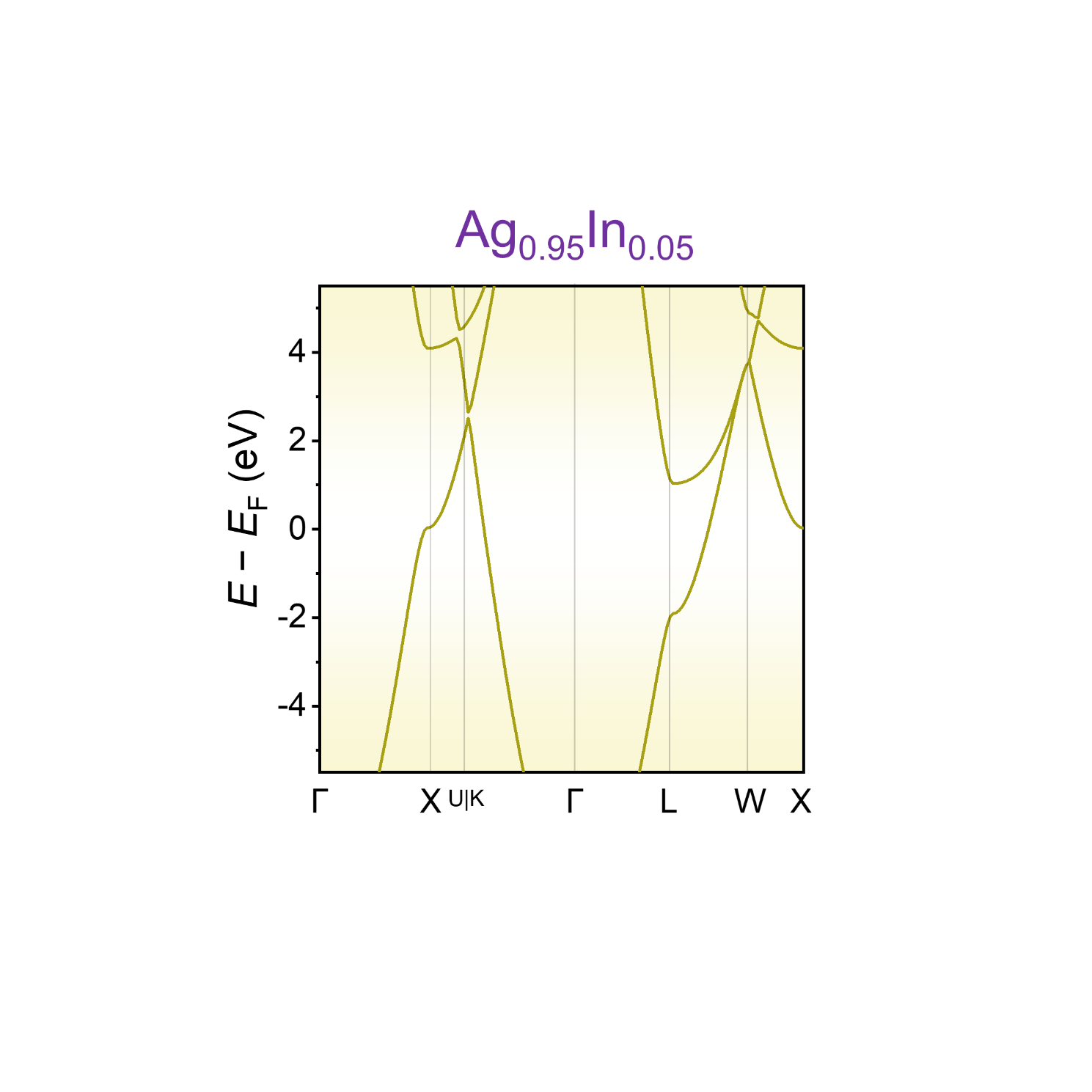


**Figure S4**. Calculated energy band structure for Ag_0.95_In_0.05_.


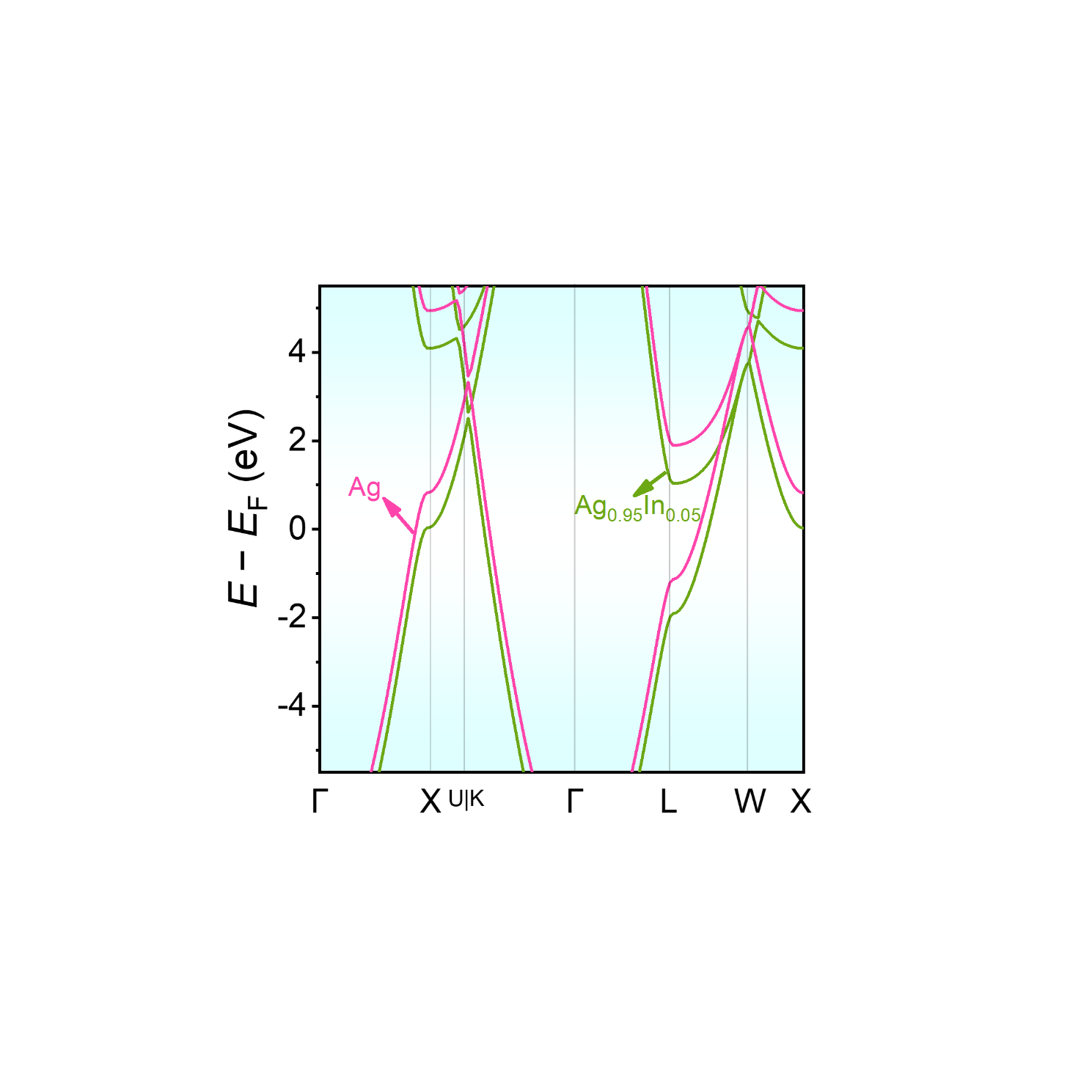


**Figure S5**. Calculated energy band structures for Ag_0.95_In_0.05_ and Ag.


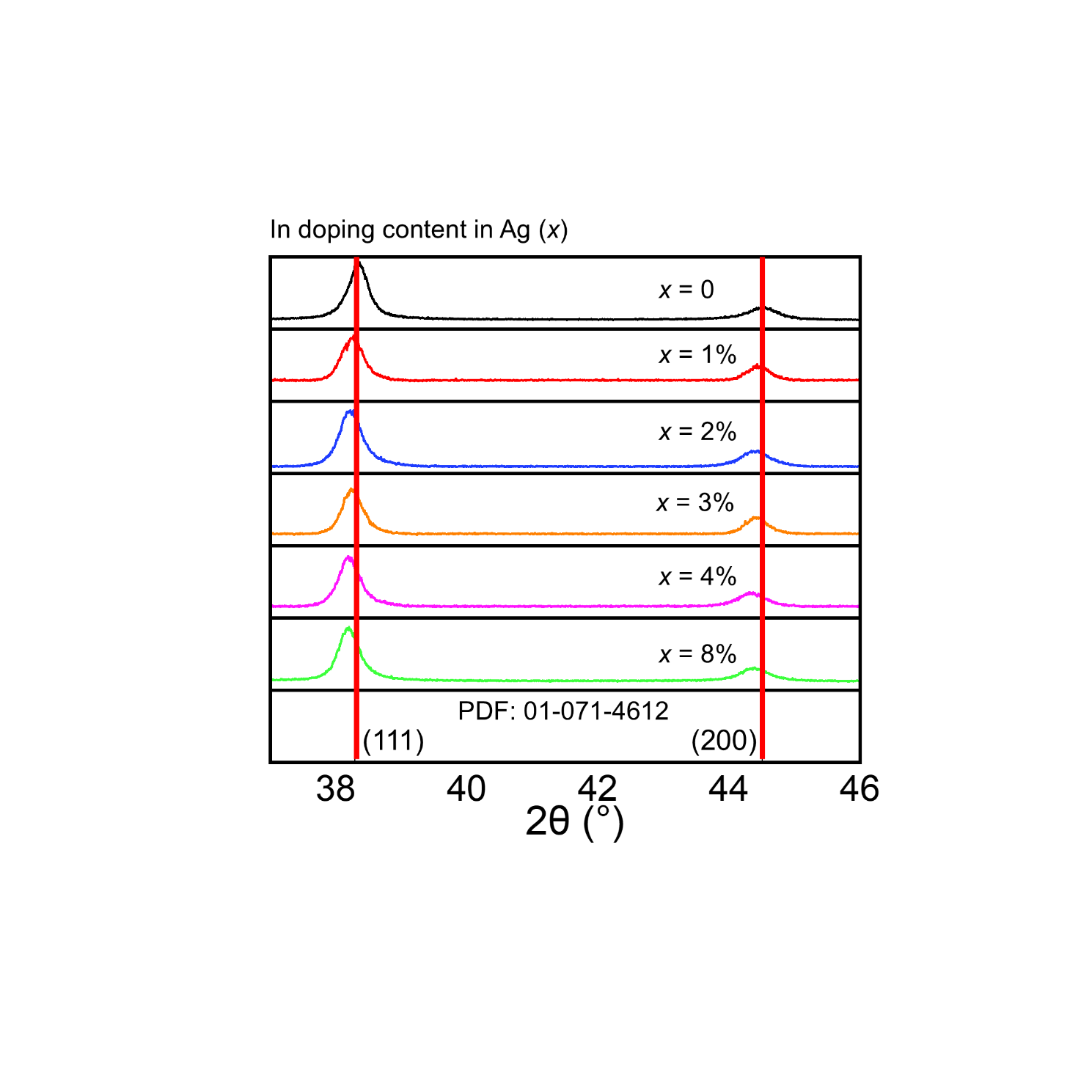


**Figure S6**. Magnified grazing incident X-ray diffraction (GIXRD) patterns of In-doped Ag with different In-doping concentrations *x* (*x* = 0, 1%, 2%, 3%, 4%, 8%).


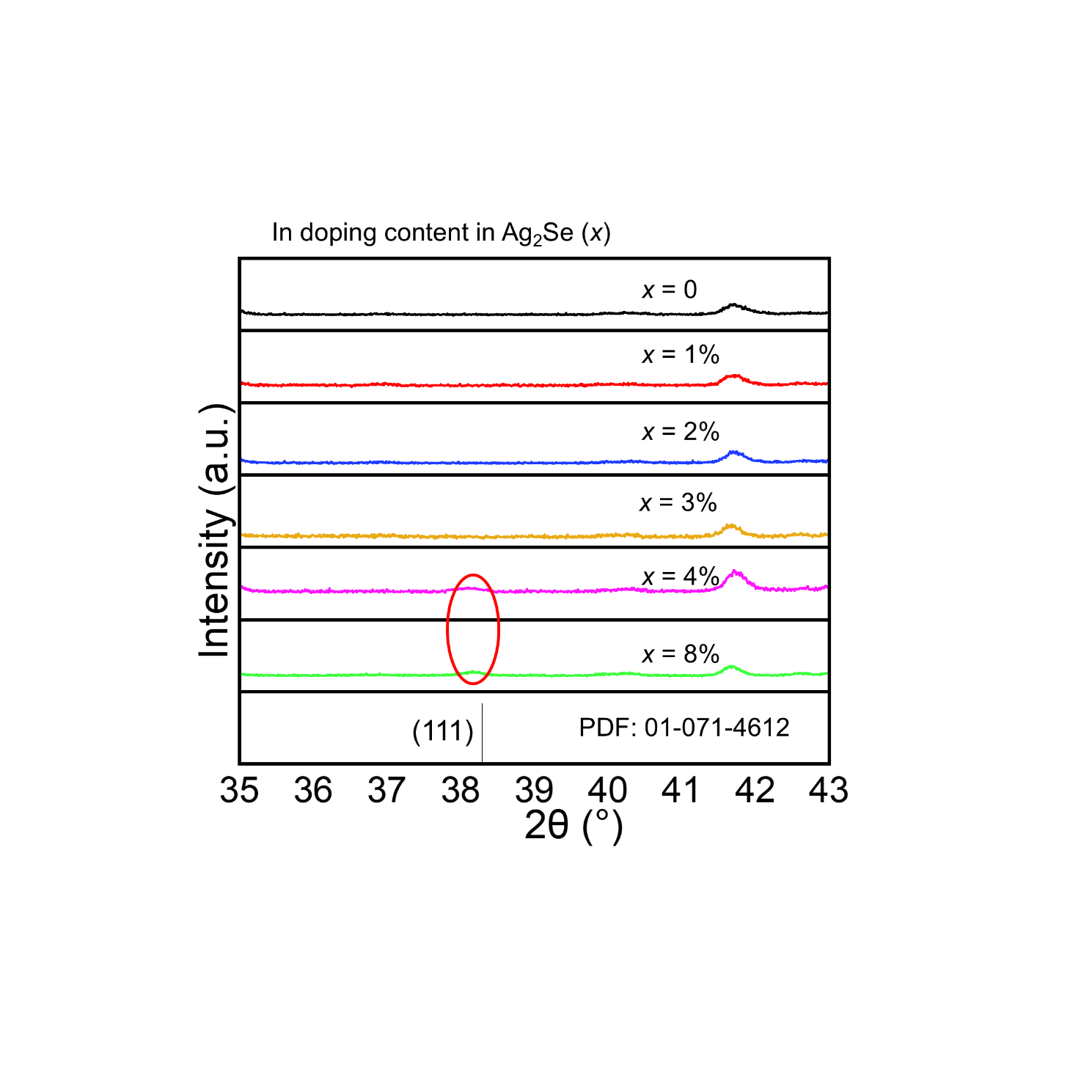


**Figure S7**. Magnified GIXRD pattern of In-doped Ag_2_Se with different In-doping concentrations (*x* = 0, 1%, 2%, 3%, 4%, 8%). The red circle is the (111) Ag peak. The powder diffraction file is Ag (#010714612).


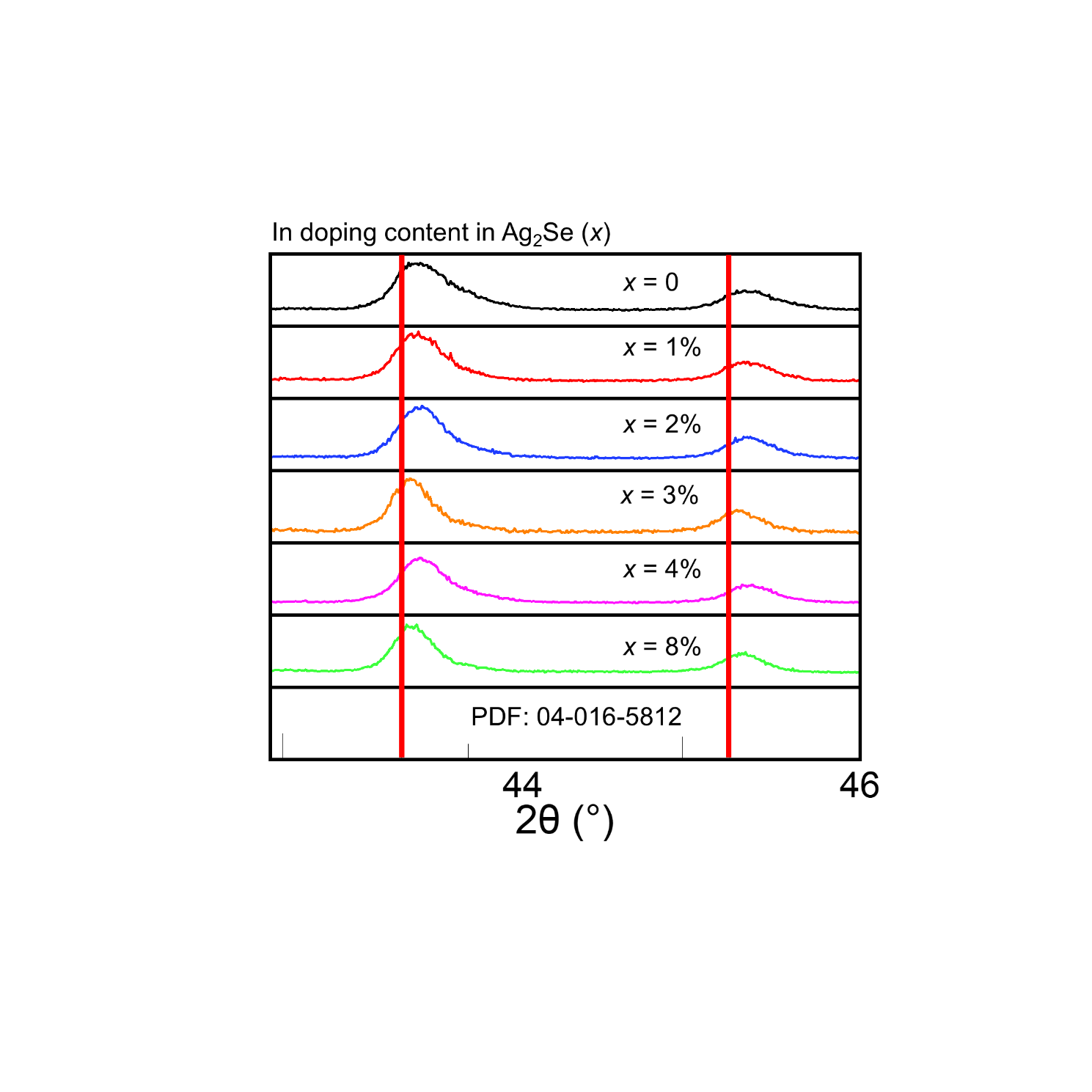


**Figure S8**. Magnified GIXRD pattern of In-doped Ag_2_Se with different In-doping concentrations (*x* = 0, 1%, 2%, 3%, 4%, 8%). The powder diffraction file is Ag_2_Se (#040165812).


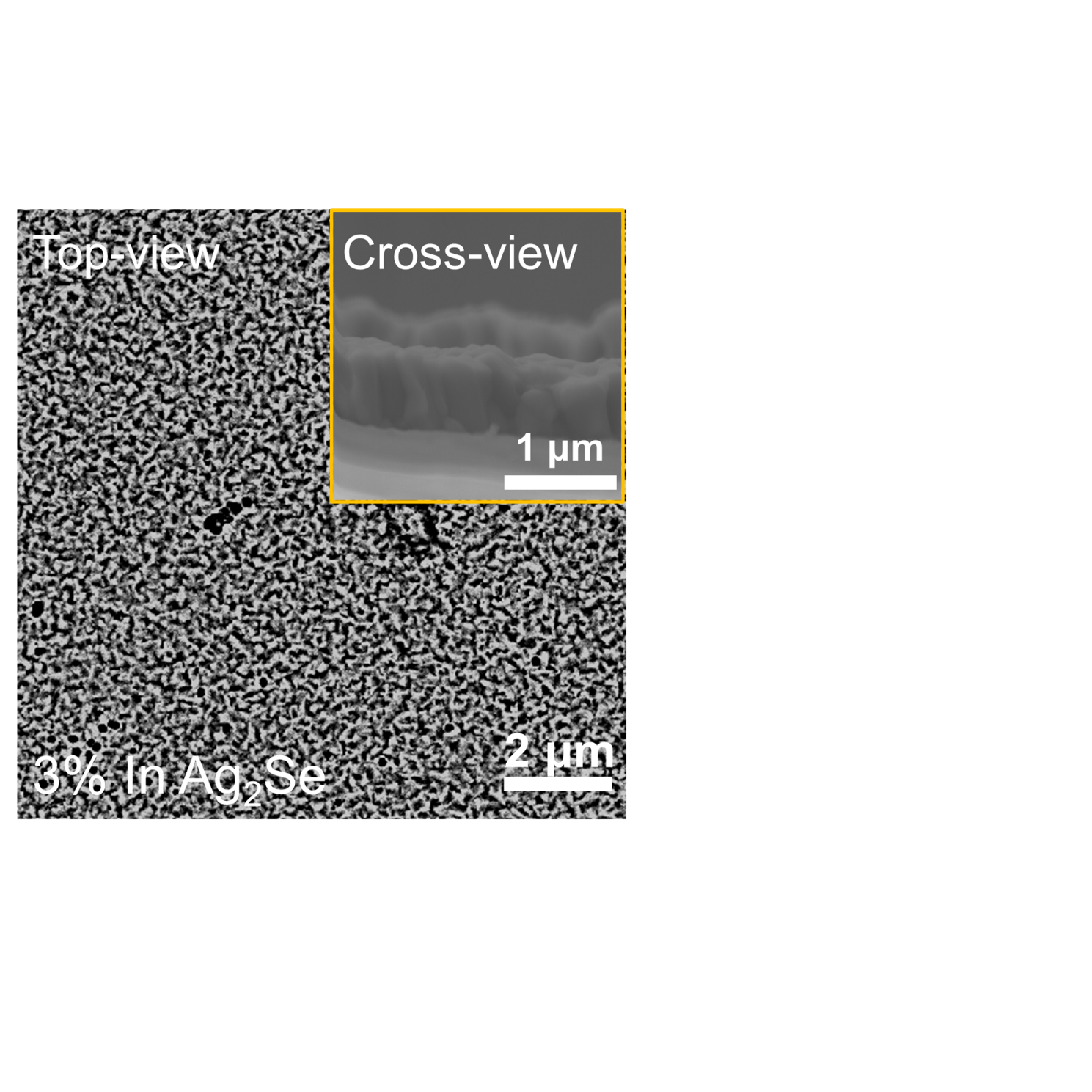


**Figure S9**. Scanning electron microscopy (SEM) back-scattering electrons (BSE) image for In-doped Ag_2_Se thin-film sample (*x* = 3%). The inset is the cross-sectional view.


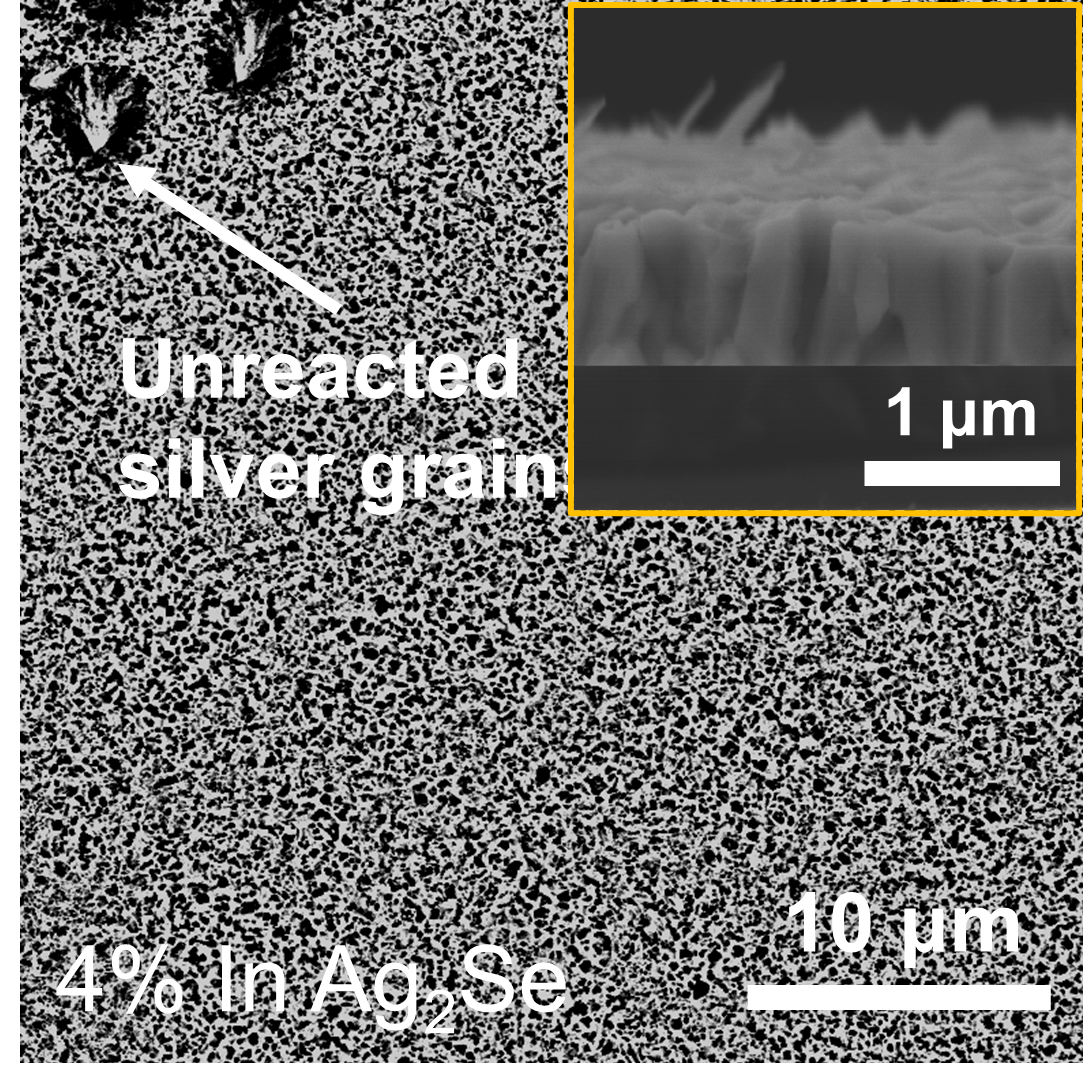


**Figure S10**. SEM-BSE image for In-doped Ag_2_Se thin-film sample (*x* = 4%). The inset is cross-sectional view.


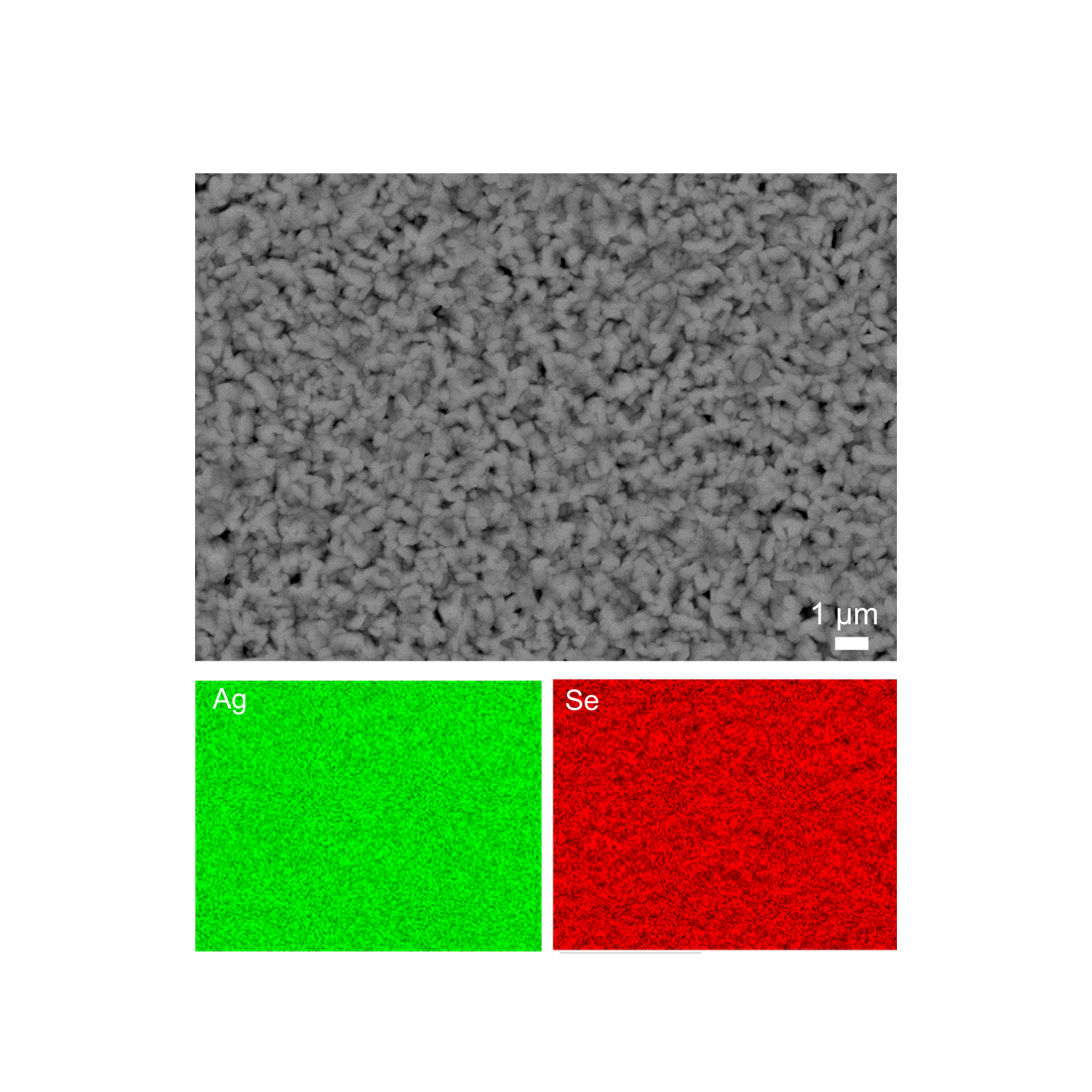


**Figure S11**. SEM-BSE image for Ag_2_Se thin-film sample without In-doping.


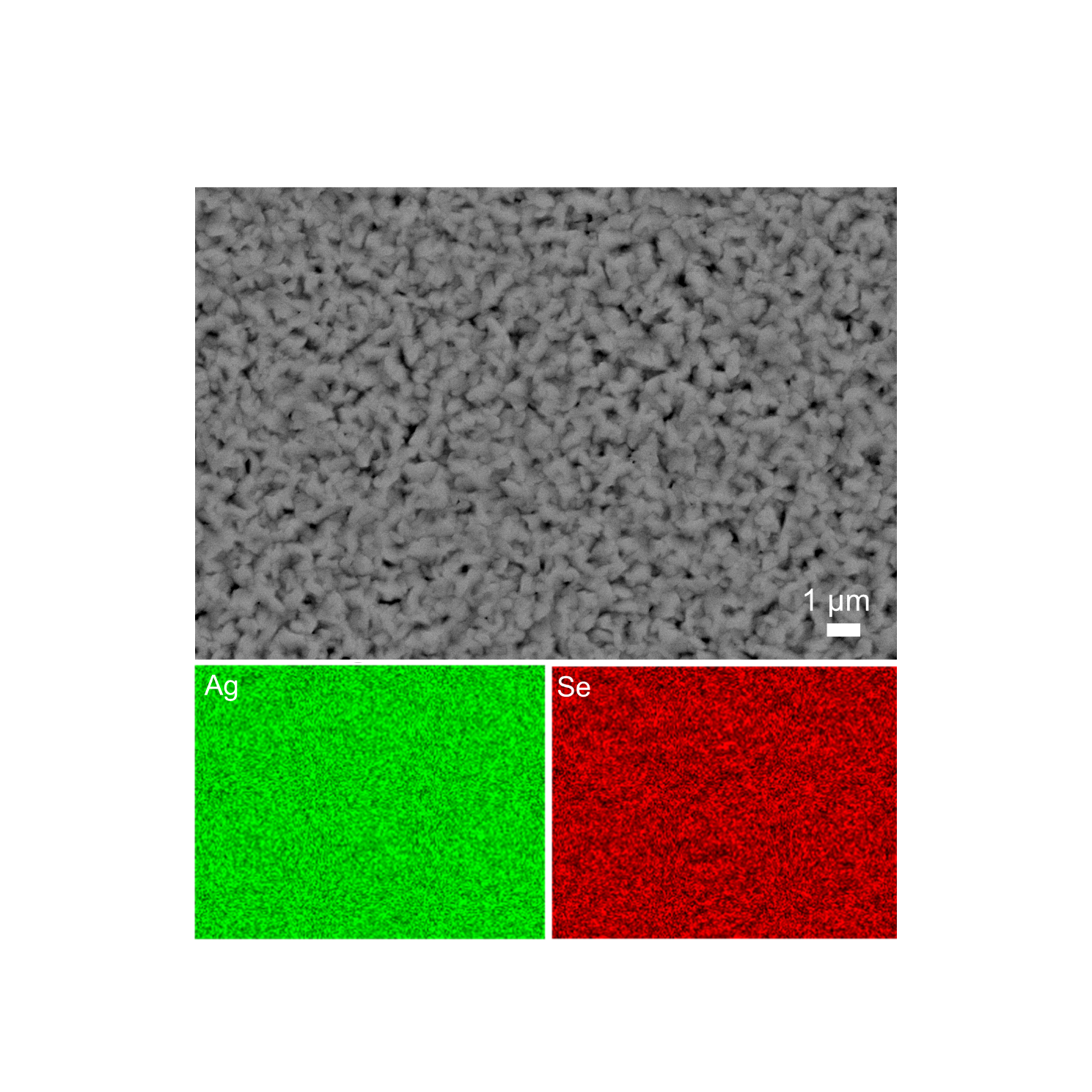


**Figure S12**. SEM-BSE image for In-doped Ag_2_Se thin-film sample (*x* = 1%).


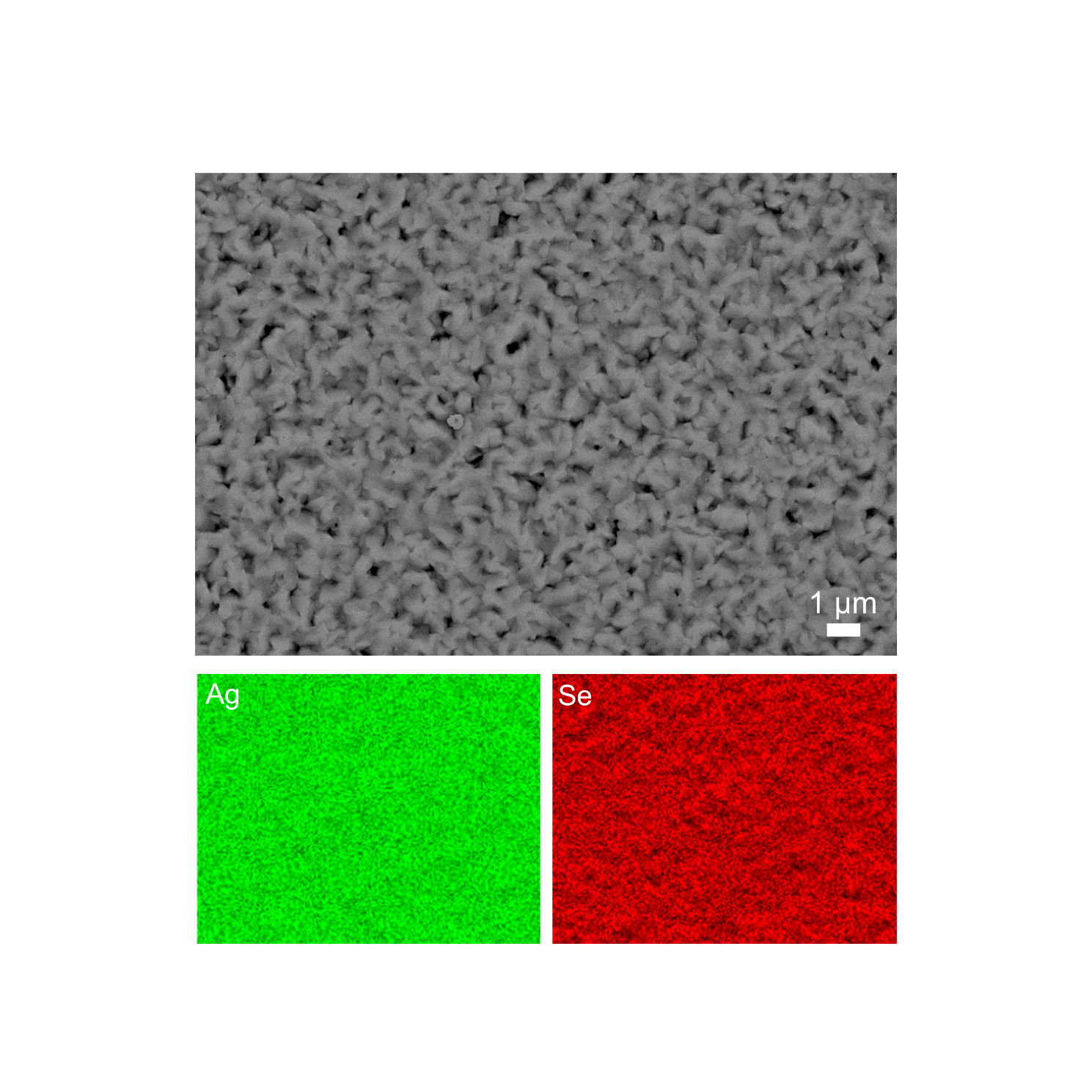


**Figure S13**. SEM-BSE image for In-doped Ag_2_Se thin-film sample (*x* = 2%).


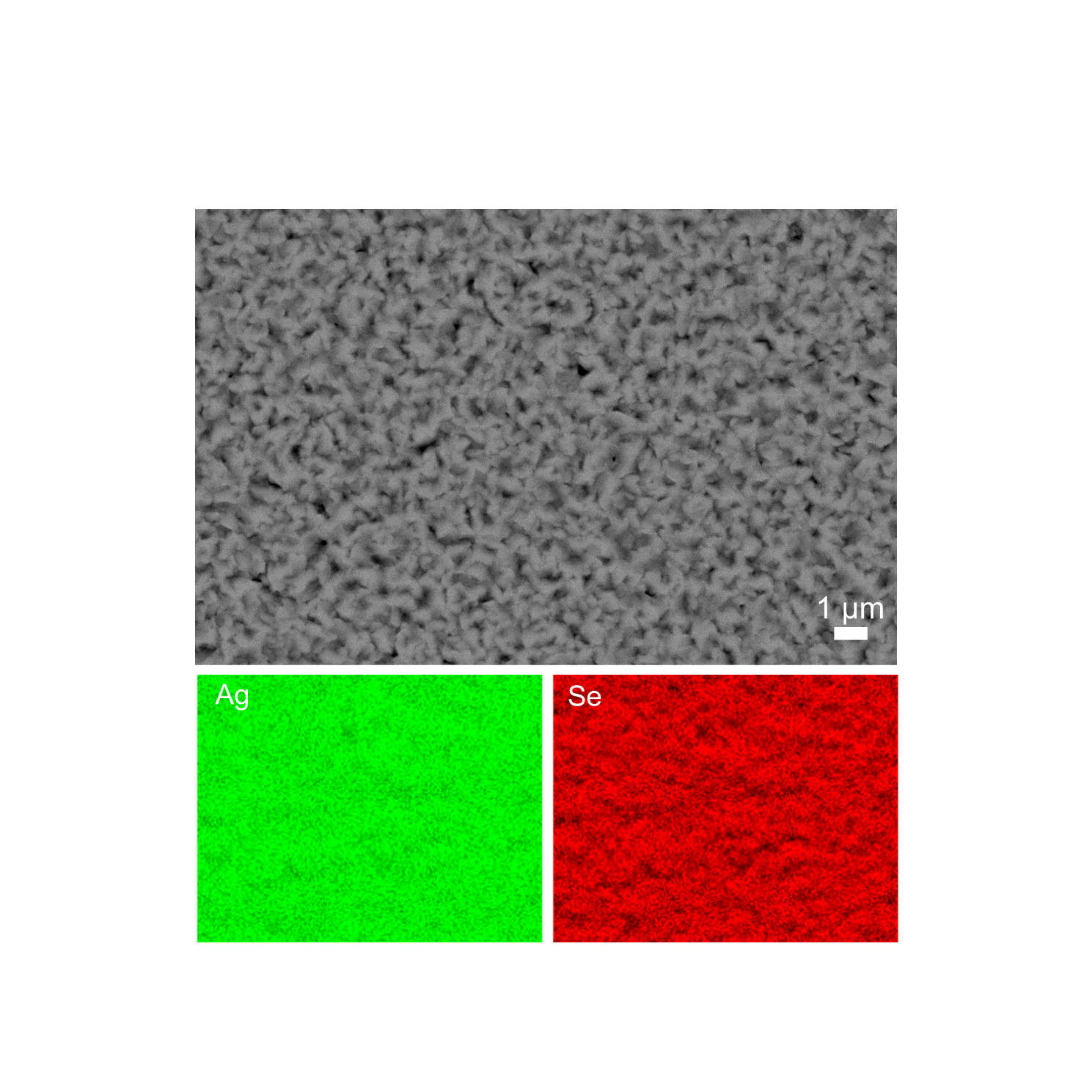


**Figure S14**. SEM-BSE image for In-doped Ag_2_Se thin-film sample (*x* = 3%).


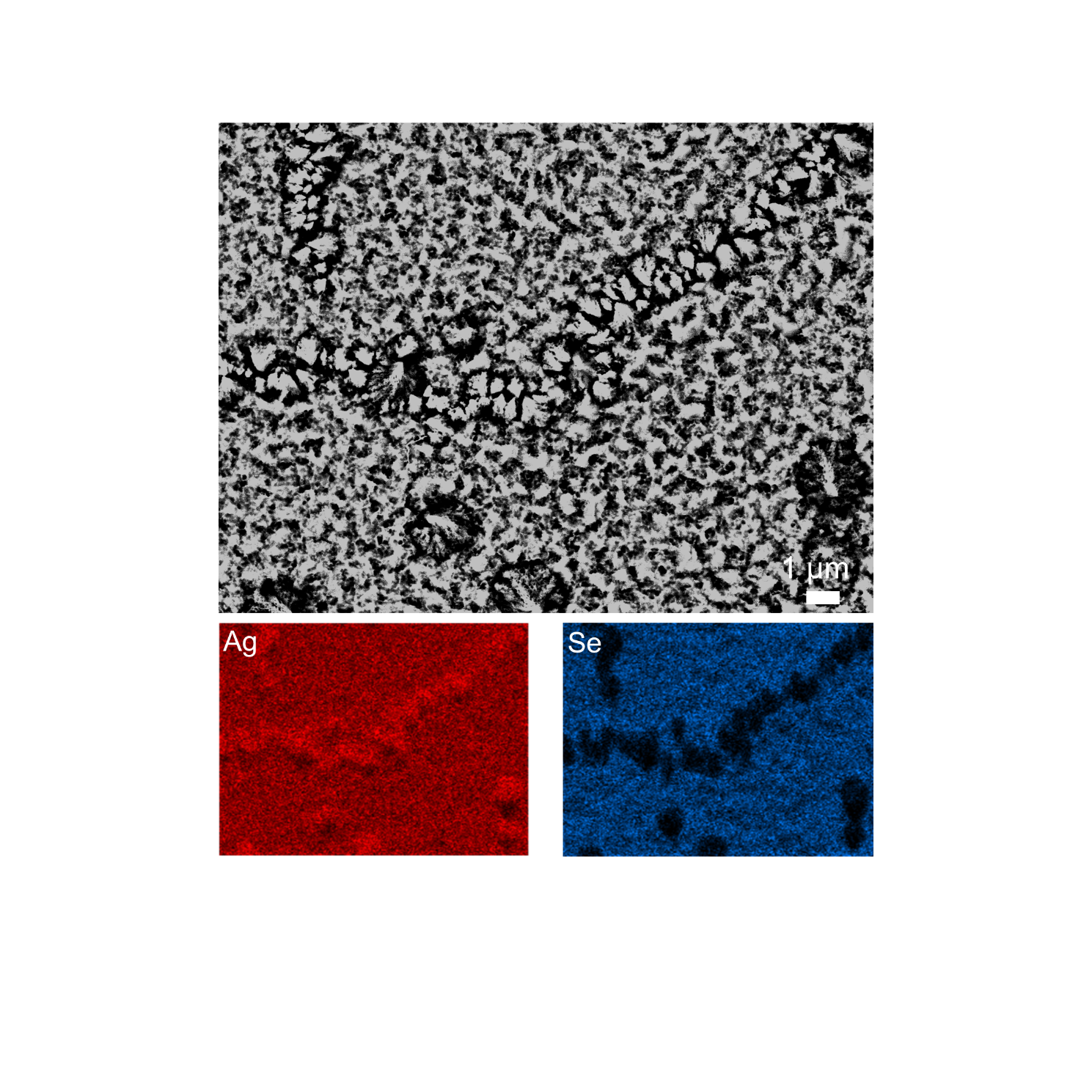


**Figure S15**. SEM-BSE image for In-doped Ag_2_Se thin-film sample (*x* = 4%).


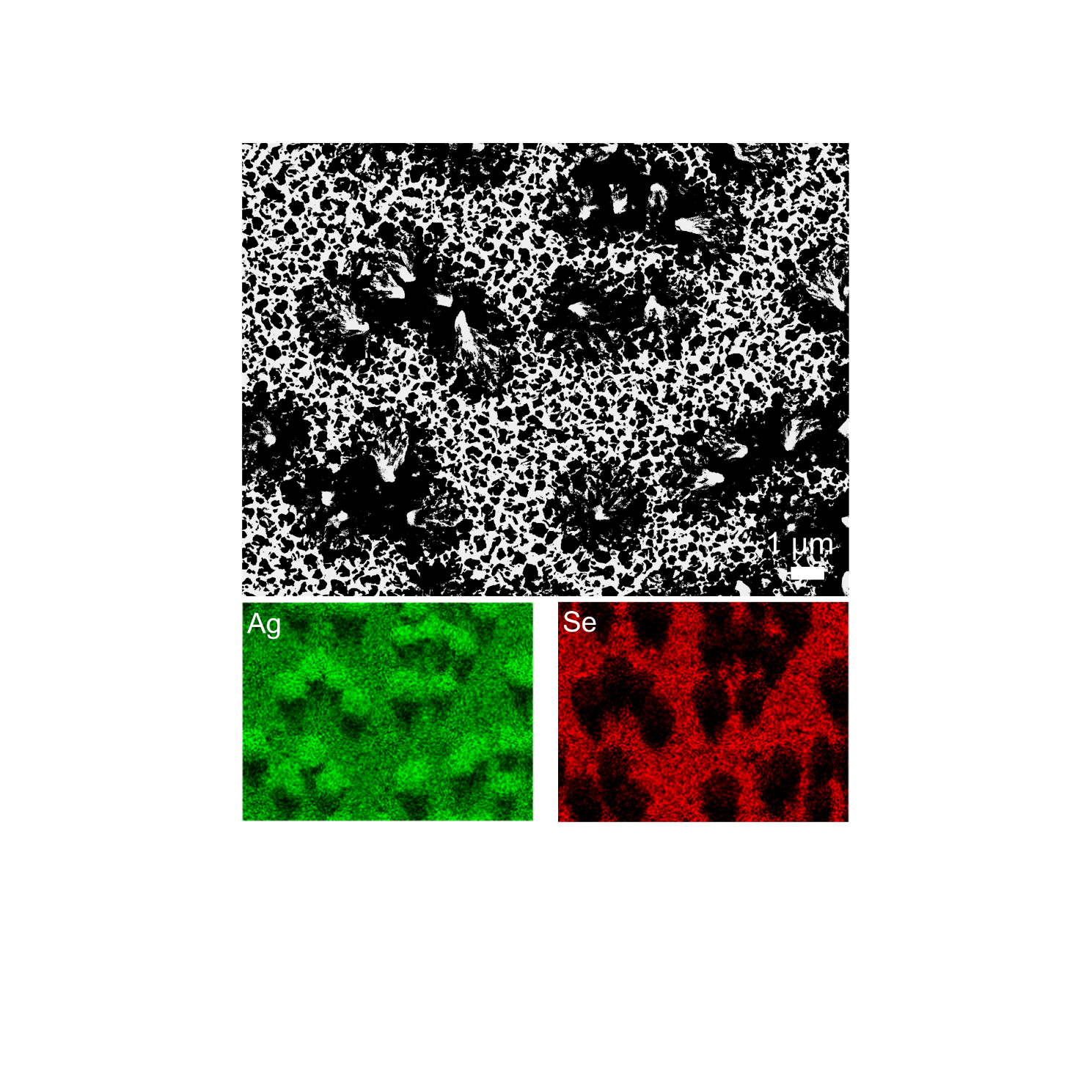


**Figure S16**. SEM BSE image for In-doped Ag_2_Se thin-film sample (*x* = 8%).


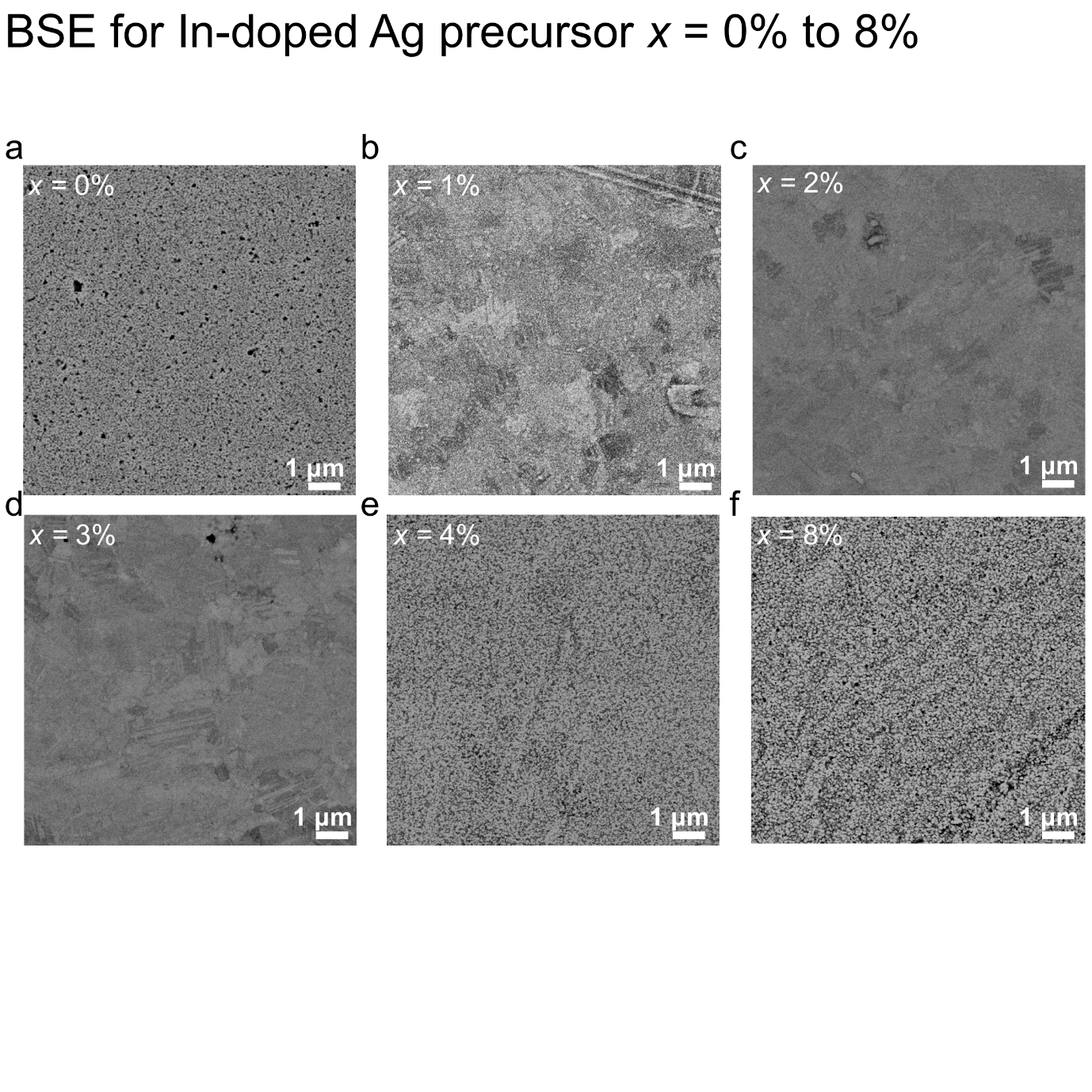


**Figure S17**. BSE image for In-doped Ag thin-film samples with (a) *x* = 0%, (b) *x* = 1%, (c) *x* = 2%, (d) *x* = 3%, (e) *x* = 4%, and (f) *x* = 8%.


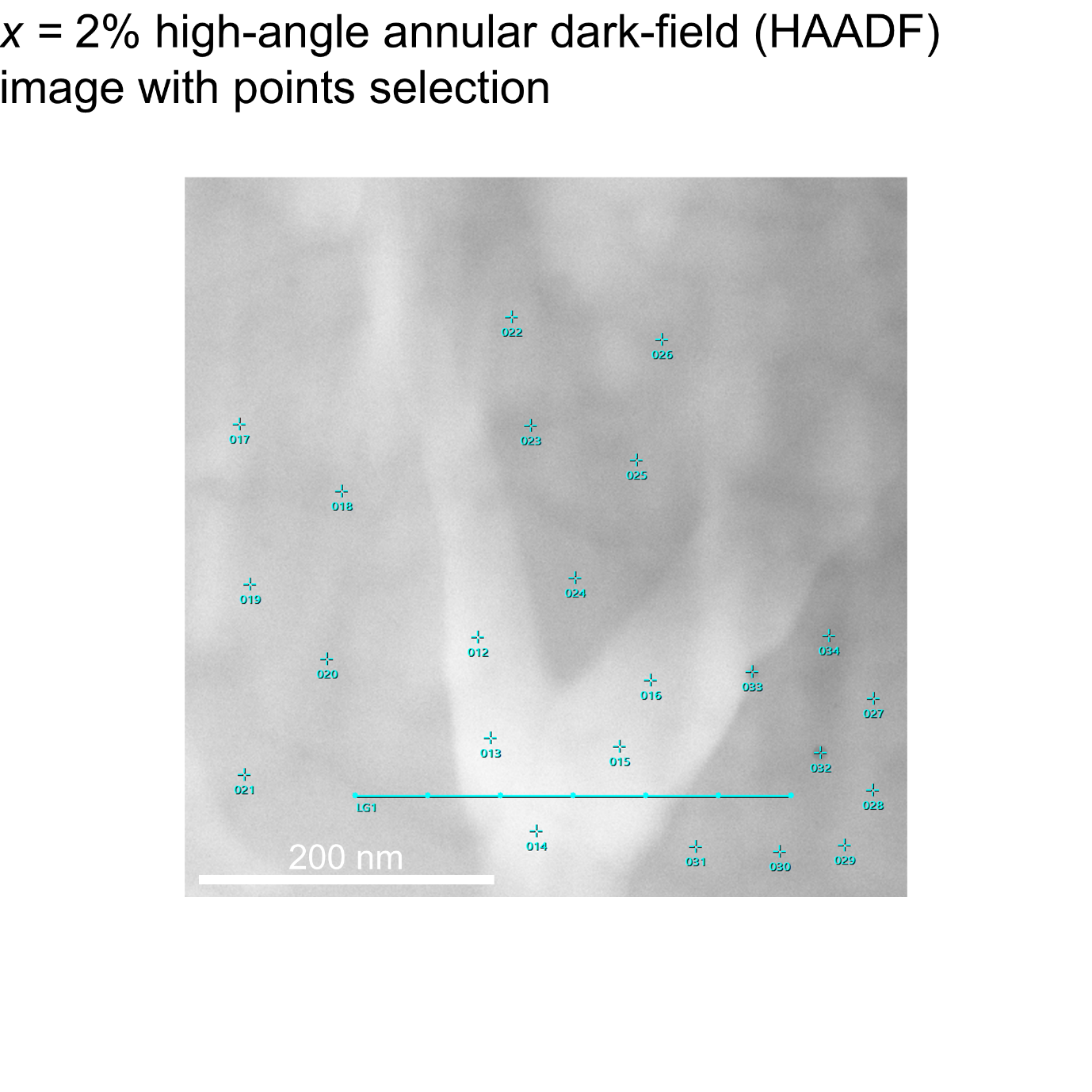


**Figure S18**. High-angle annular dark-field (HAADF) image of In-doped Ag_2_Se thin-film sample with *x* = 2% and the selected line and points for energy dispersive X-ray spectroscopy (EDS). Corresponding EDS results can be seen in **Table S1** and **S2**.


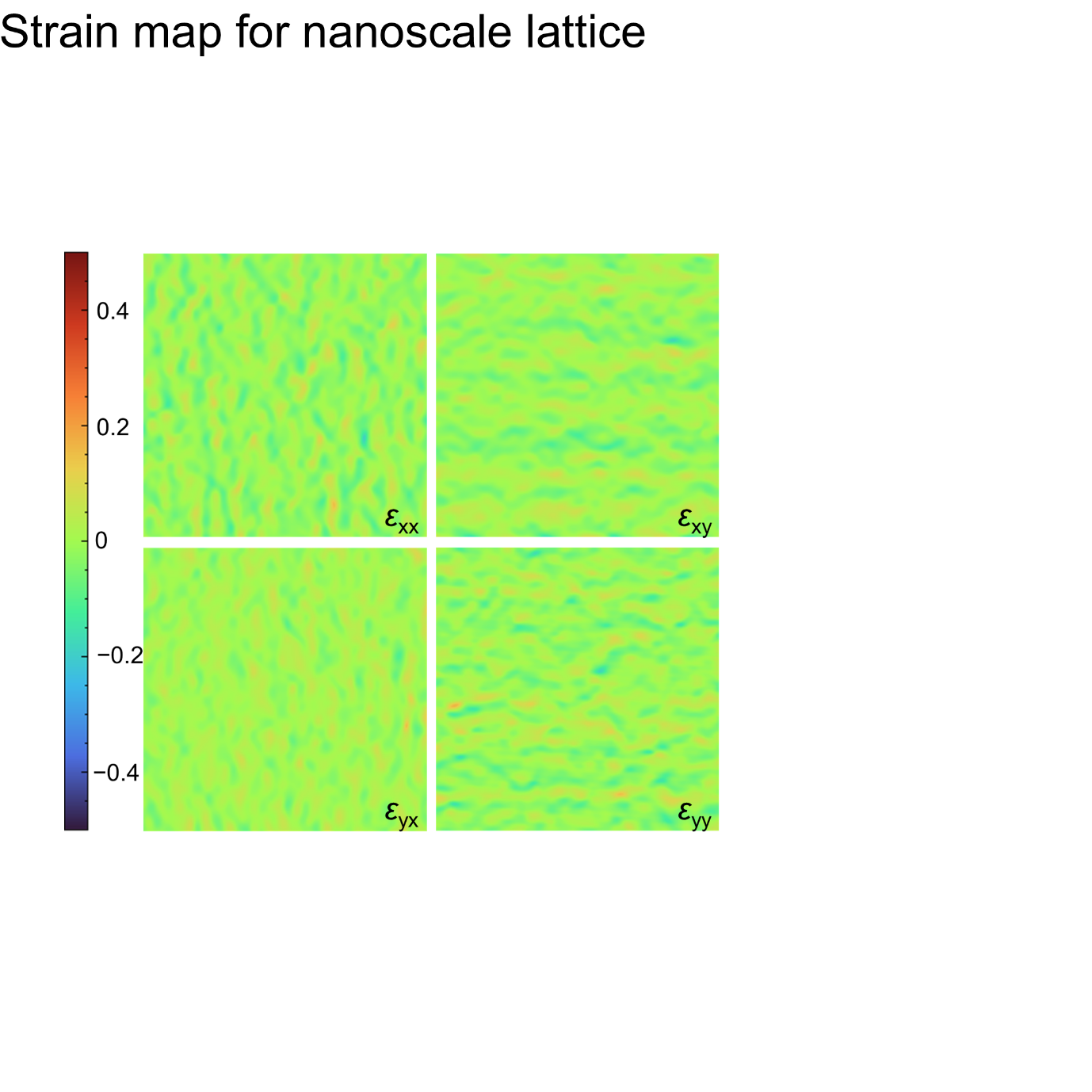


**Figure S19**. Strain maps for **Figure 3c** in the main text.


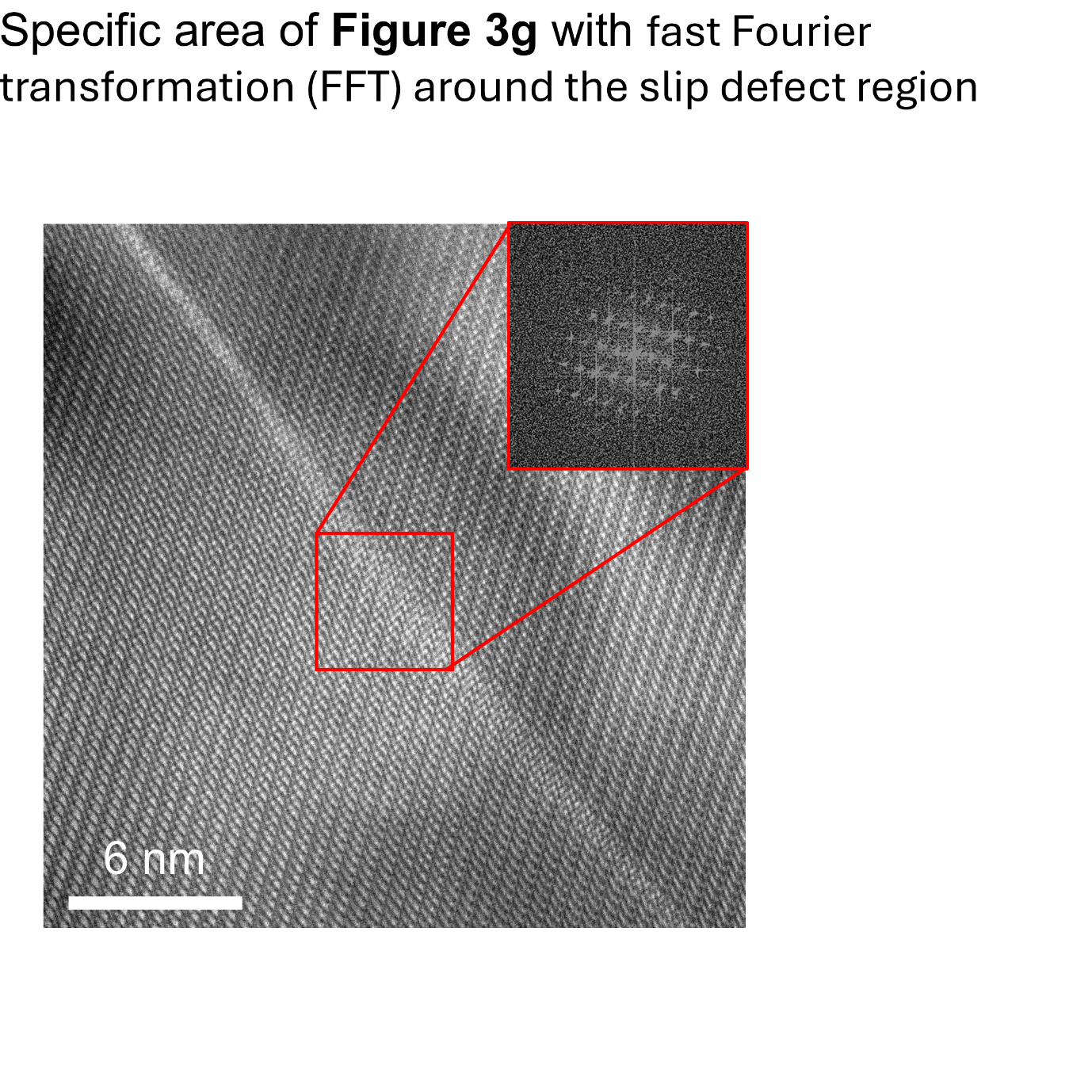


**Figure S20**. Magnified view of the slip defect based on **Figure 3g**. The inset shows the fast Fourier transform (FFT) of the red-selected region.


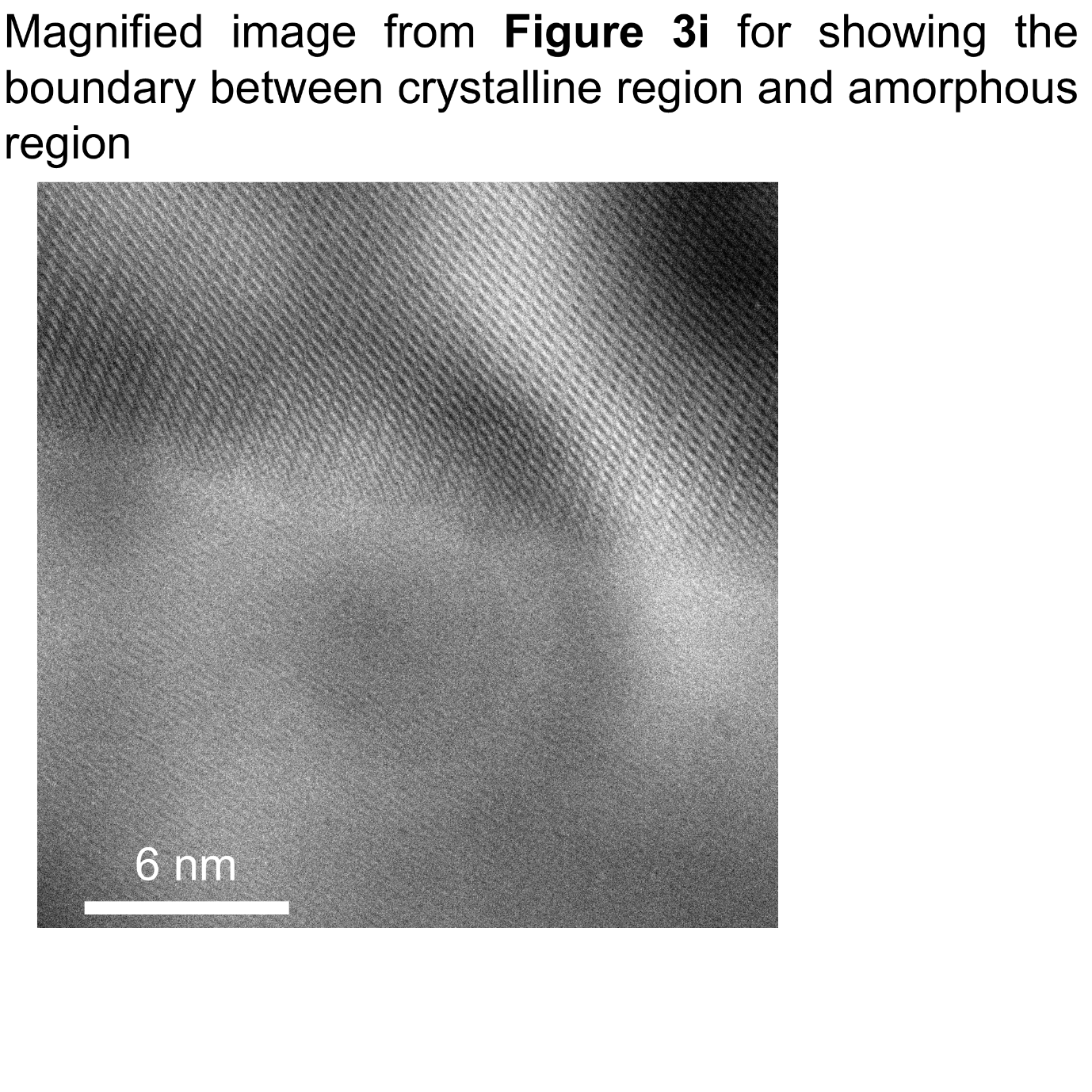


**Figure S21**. Localized magnified view of the crystalline-amorphous interface based on **Figure 3i**.


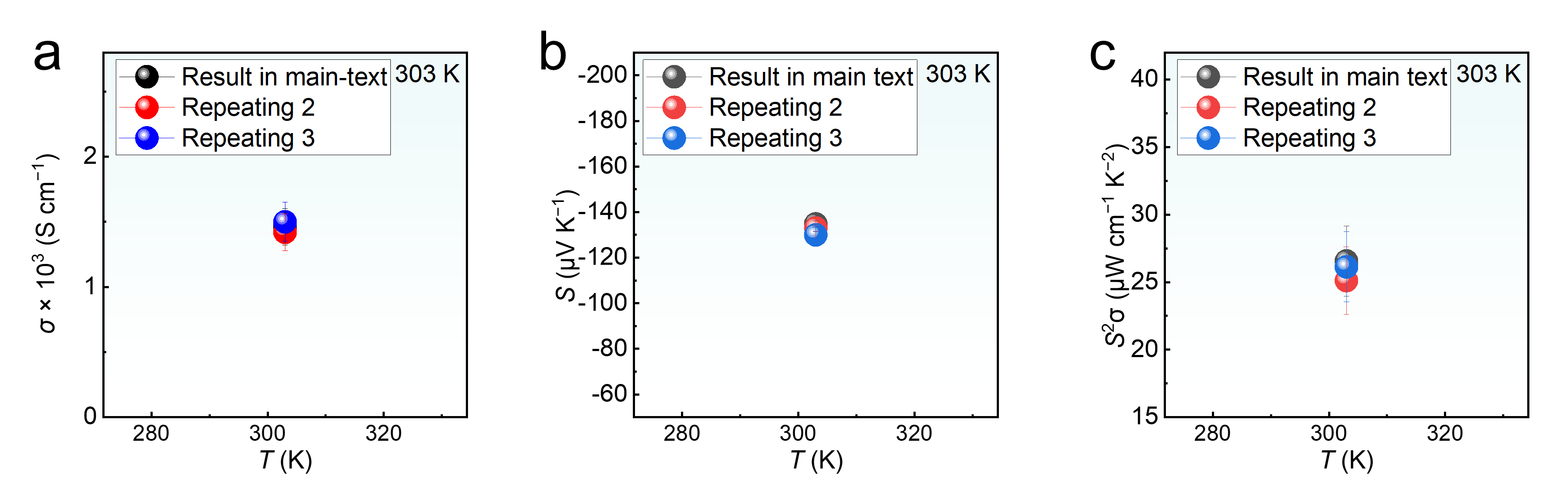


**Figure S22**. Reproducibility test of room-temperature performance for the sample with *x* = 2%, including (a) electrical conductivity (*σ*), (b) Seebeck coefficient (*S*), and (c) power factor (*S*^2^*σ*).


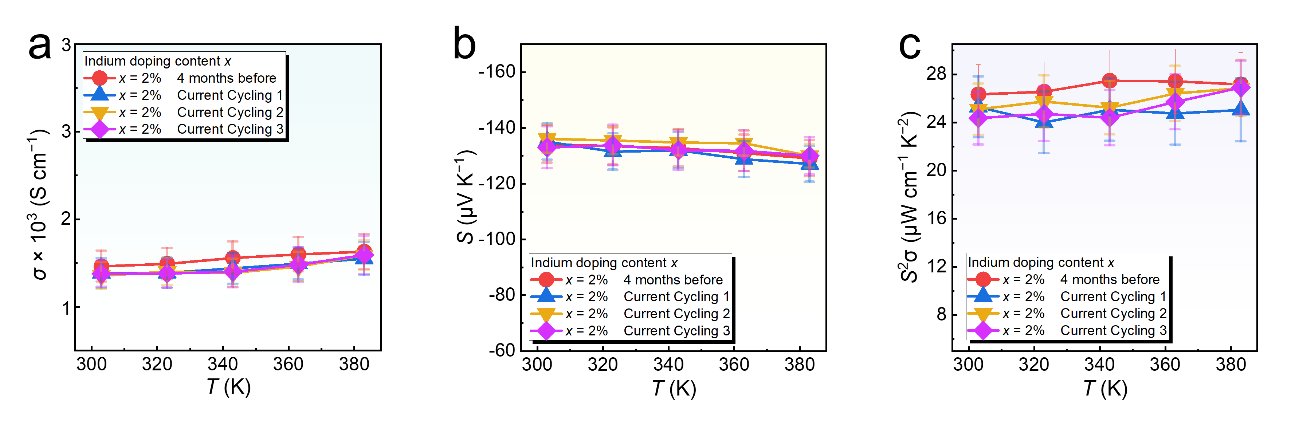


**Figure S23**.Temperature-dependent (a) *σ*, (b) *S*, and (c) *S*^2^*σ* as repeating test for sample with 2% In-doping.


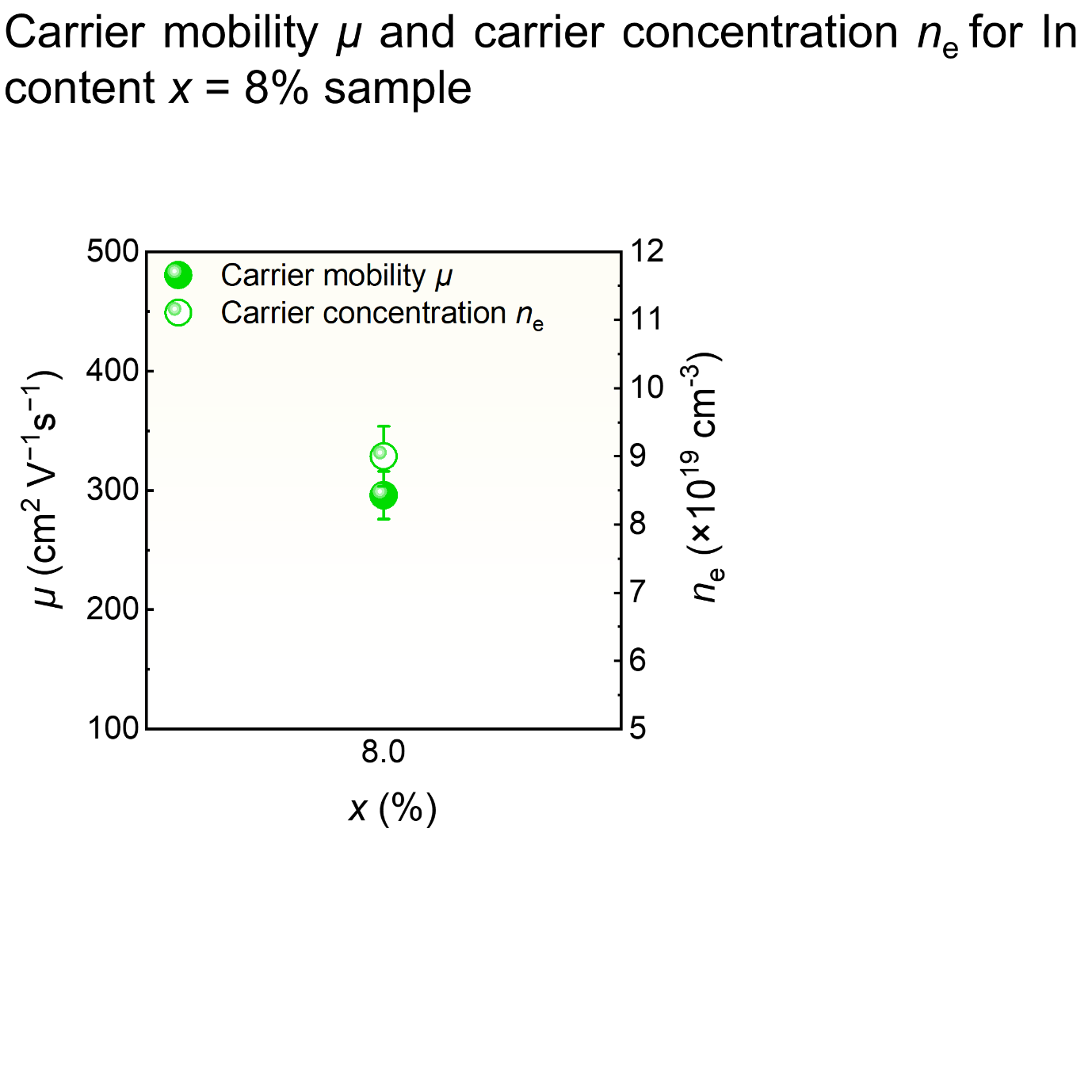


**Figure S24**. Room-temperature carrier concentration (*n*) and mobility (*μ*) of the sample with *x* = 8%.


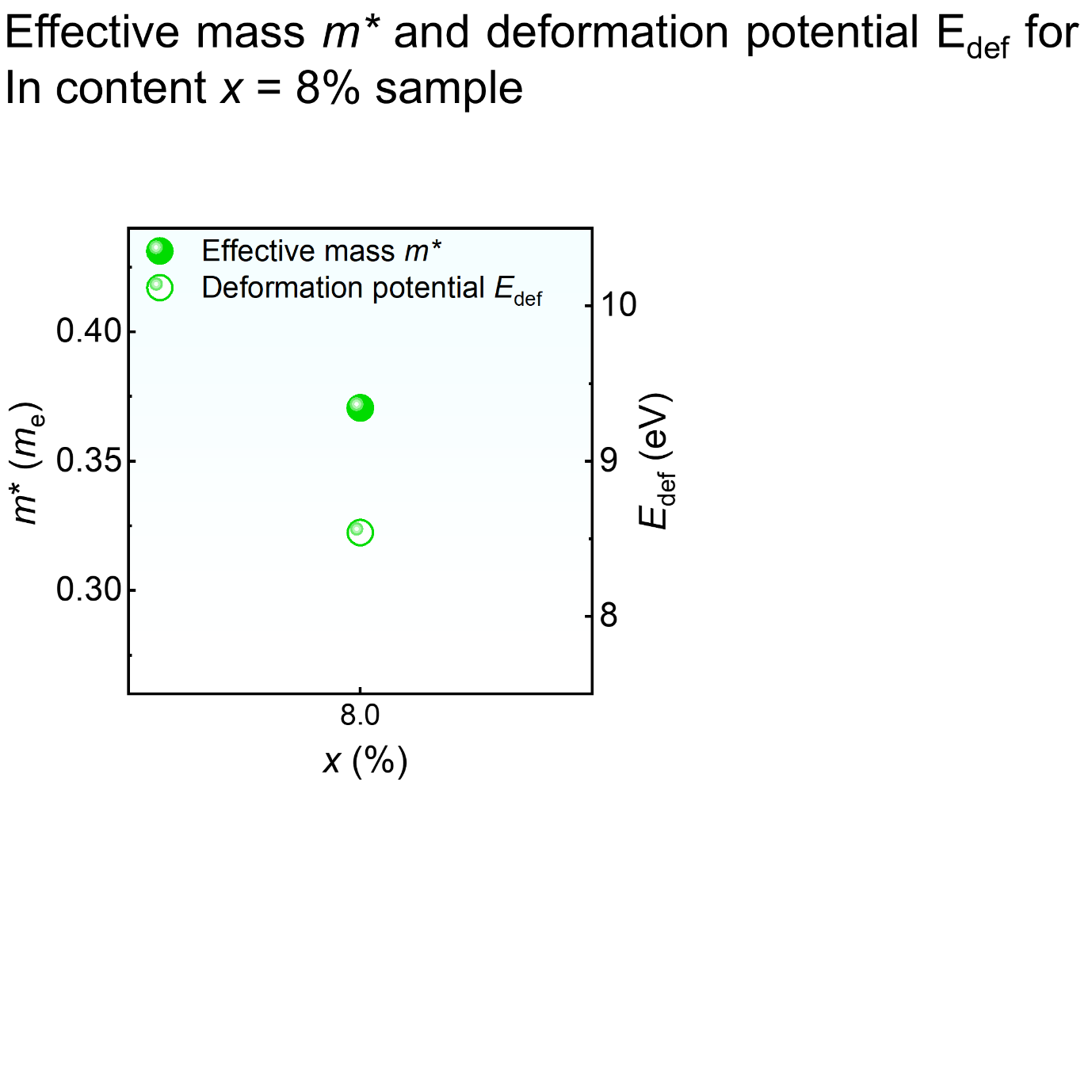


**Figure S25**. Room-temperature effective mass (*m**) and deformation potential (*E*_def_) of the sample with *x* = 8%.


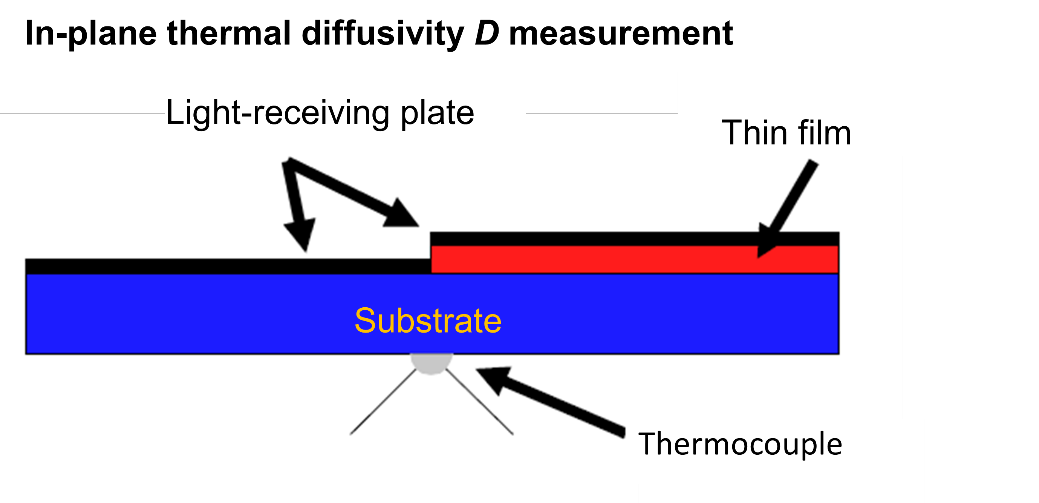


**Figure S26**. Schematic of thermal diffusivity (*D*) measurement of the thin film by employing Laser-PIT.


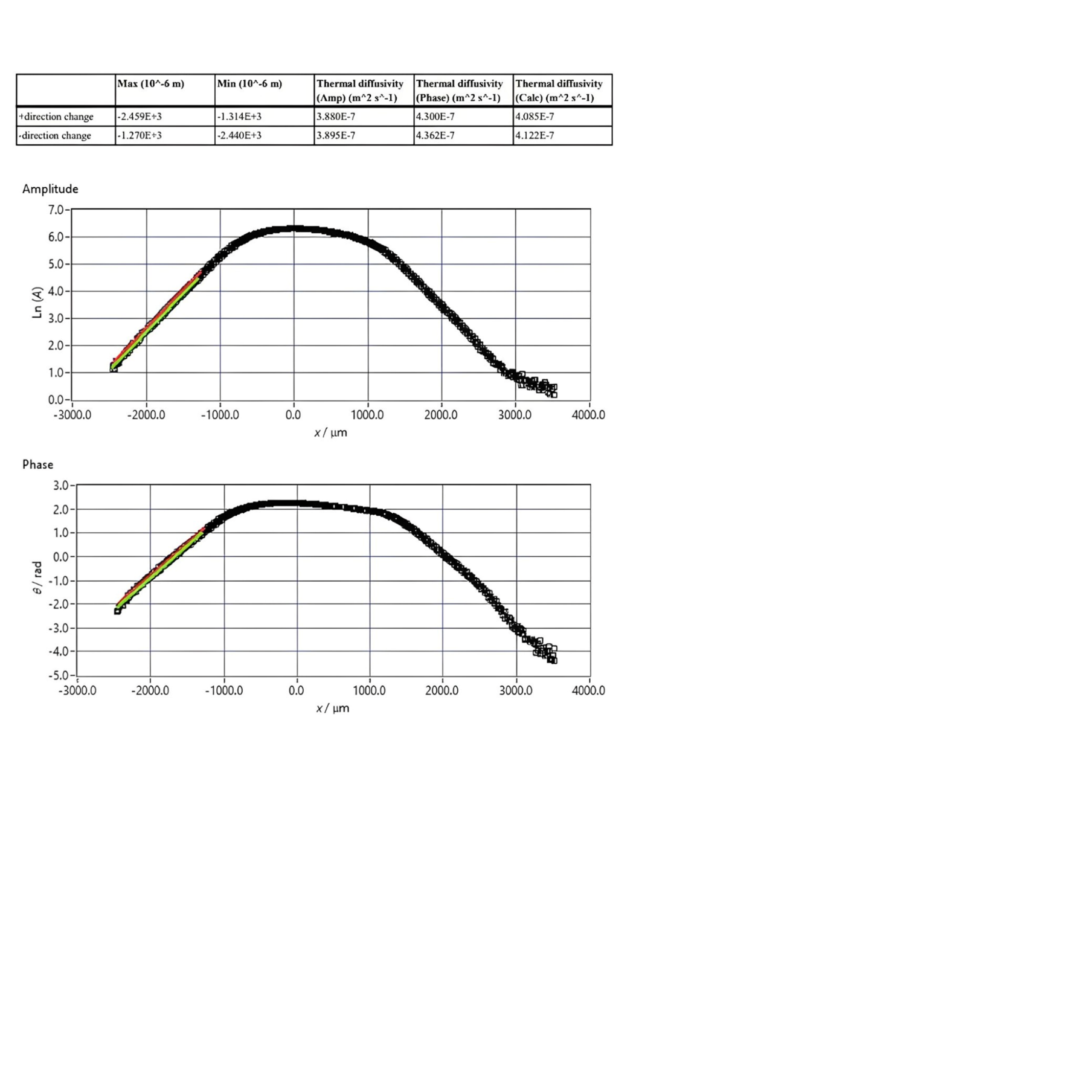


**Figure S27**. Diagram of the distance dependence of logarithmic amplitude and phase for determining the *D* for Ag_2_Se thin-film sample with In-doping level of *x* = 0%.


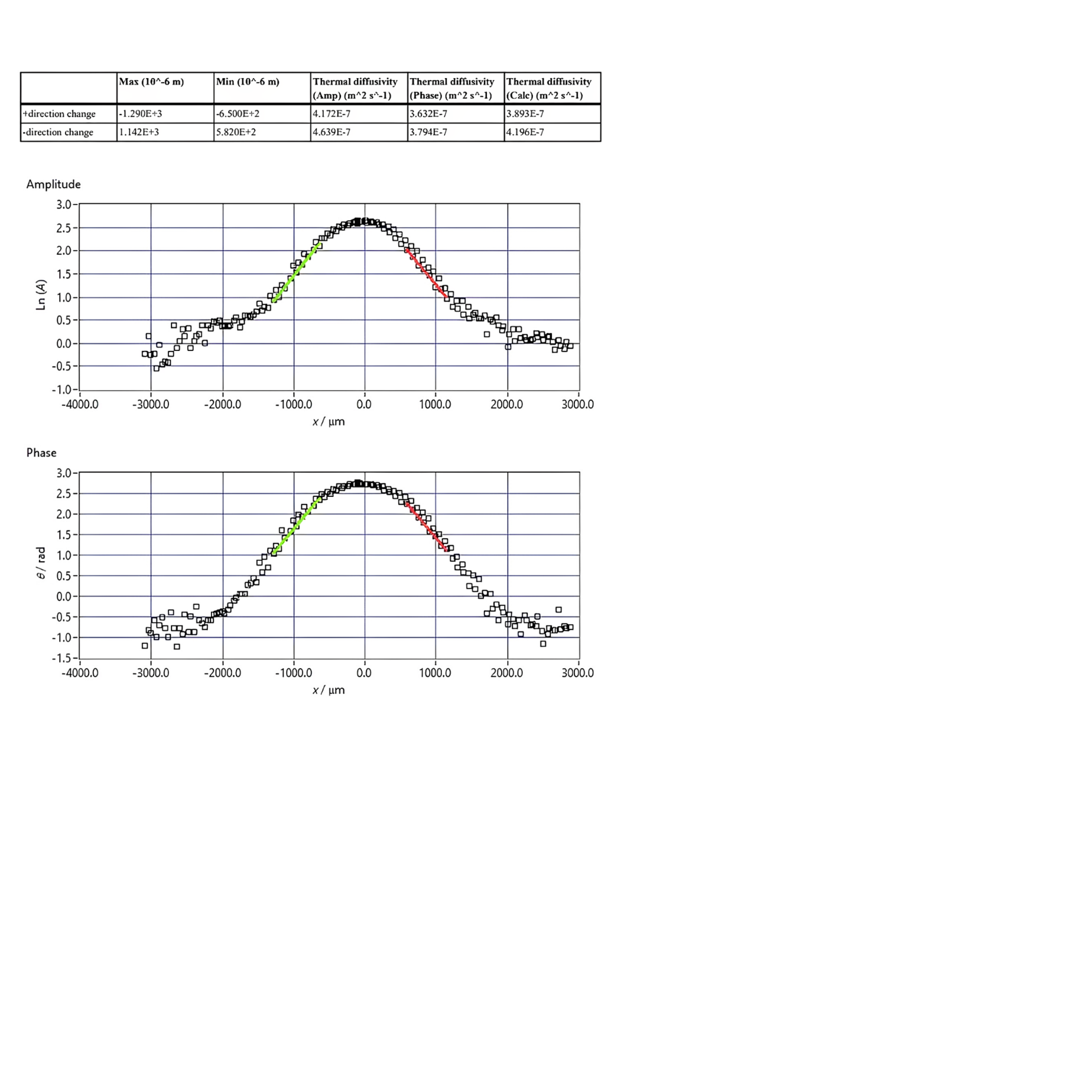


**Figure S28**. Diagram of the distance dependence of logarithmic amplitude and phase for determining the *D* for Ag_2_Se thin-film sample with In-doping level of *x* = 1%.


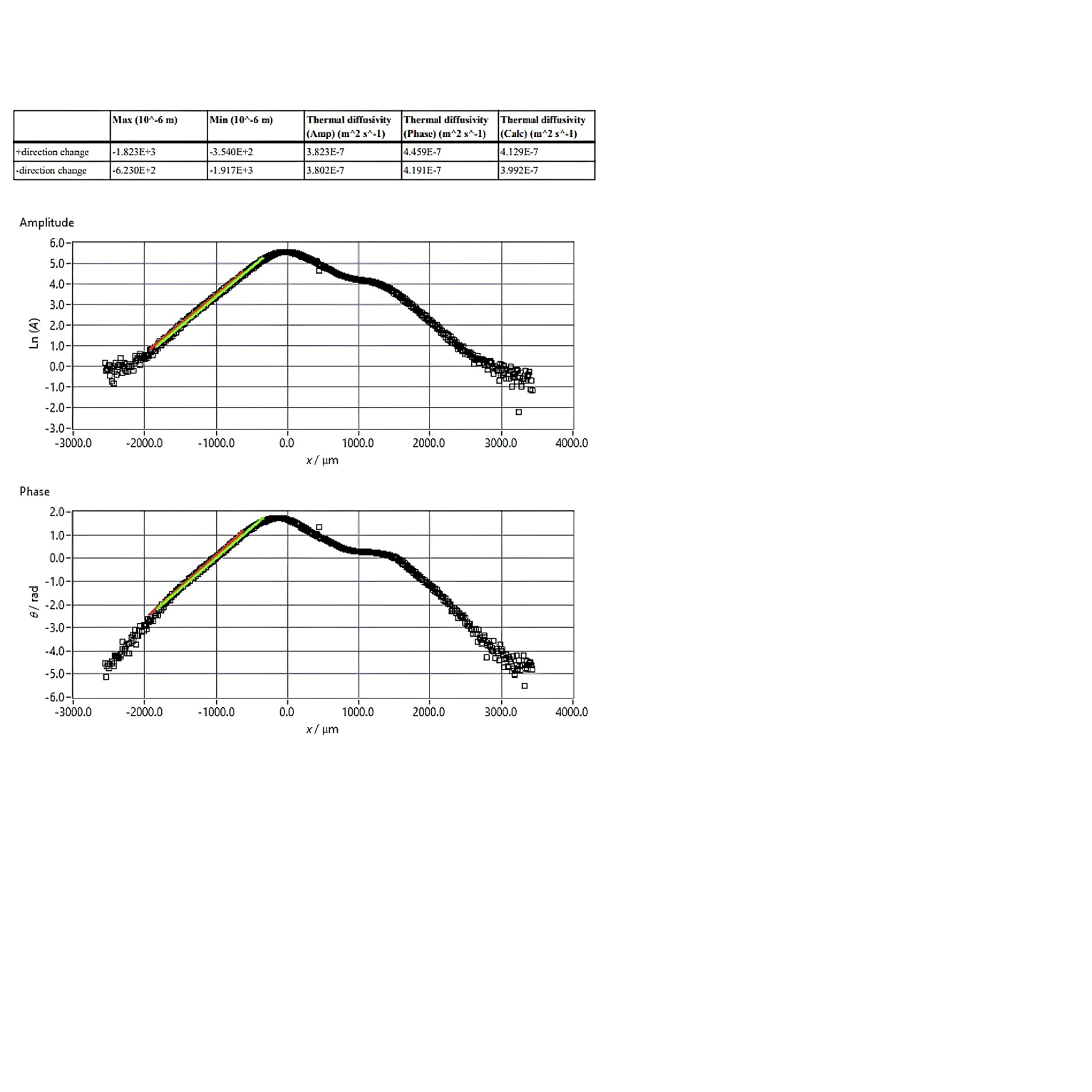


**Figure S29**. Diagram of the distance dependence of logarithmic amplitude and phase for determining the *D* for Ag_2_Se thin-film sample with In-doping level of *x* = 2%.


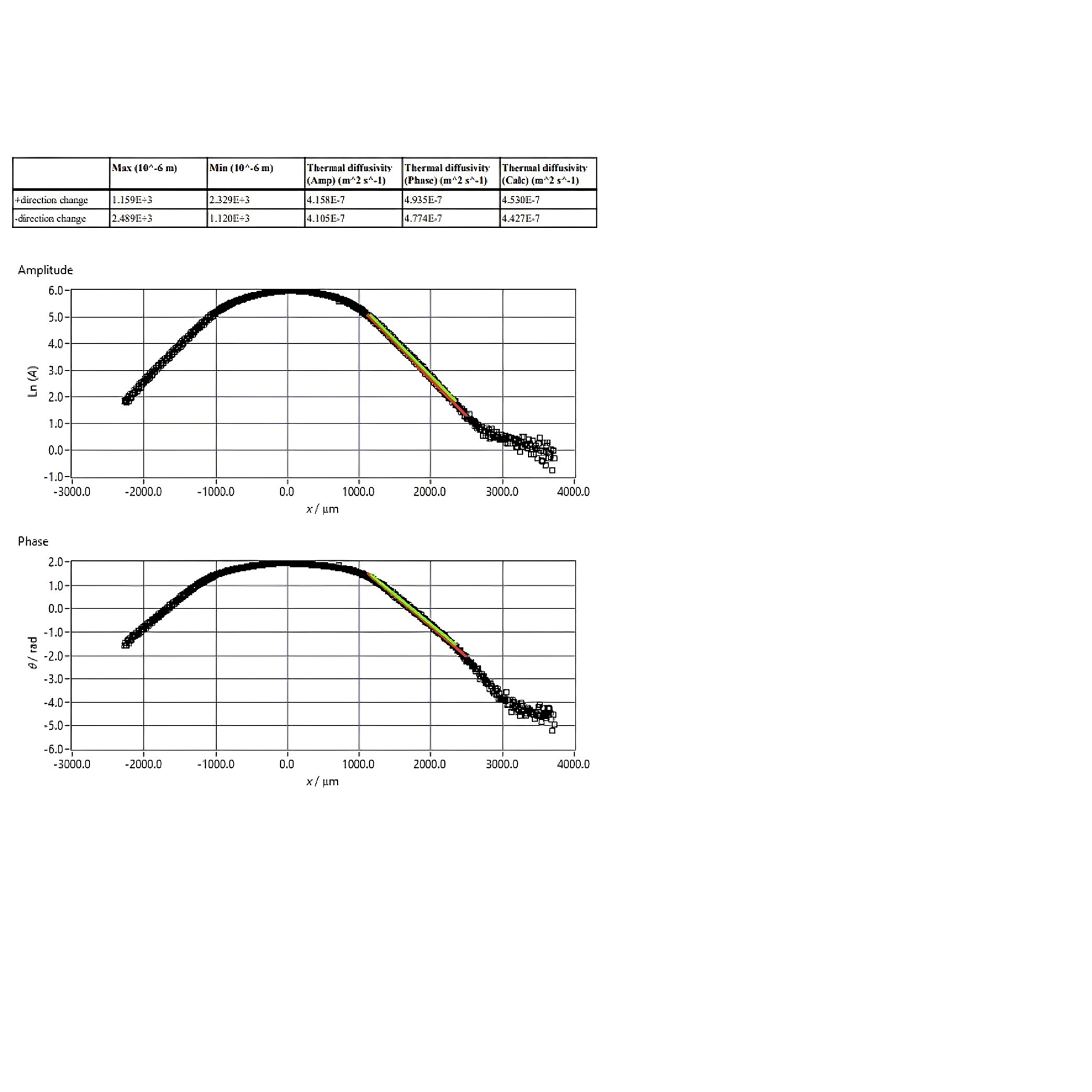


**Figure S30**. Diagram of the distance dependence of logarithmic amplitude and phase for determining the *D* for Ag_2_Se thin-film sample with In-doping level of *x* = 3%.


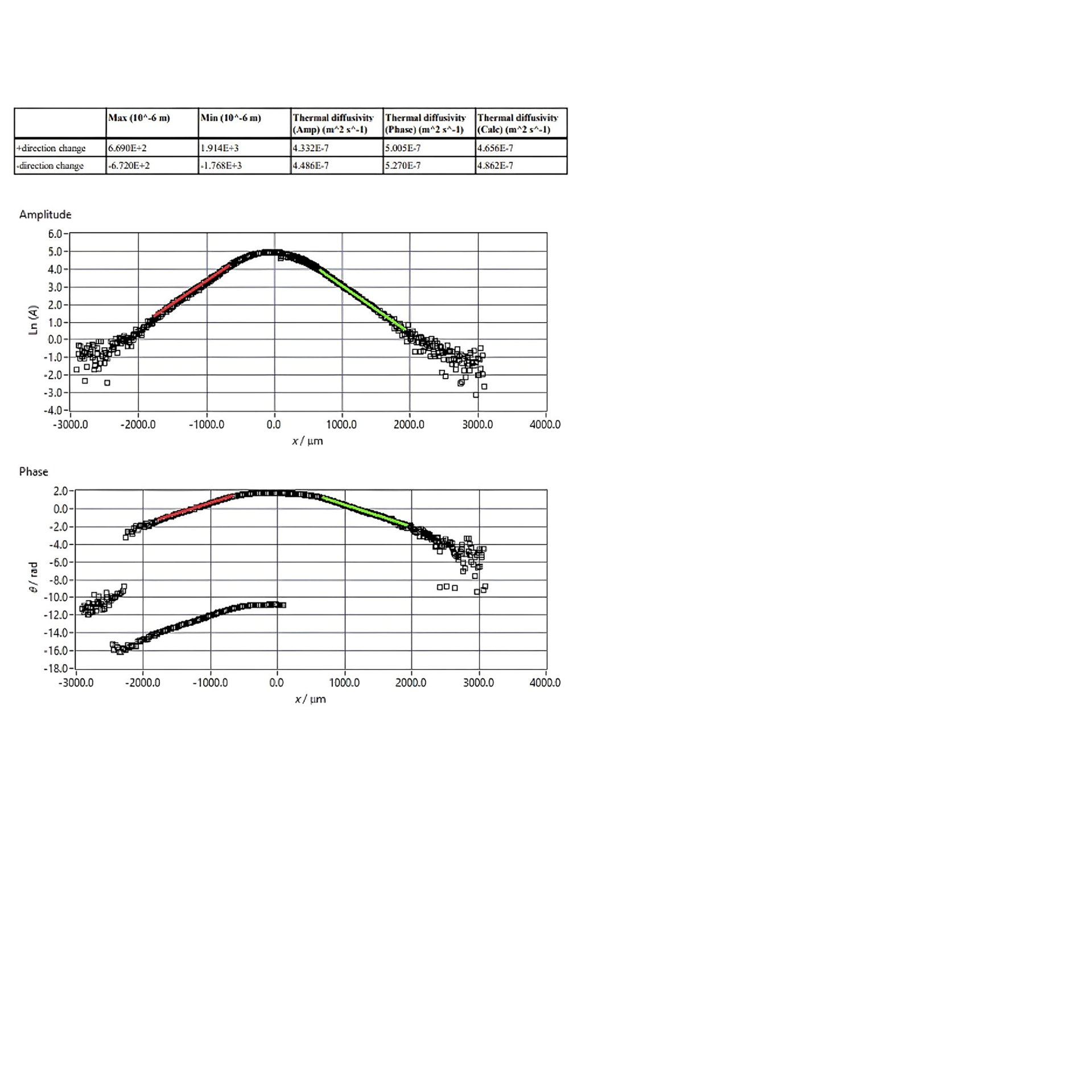


**Figure S31**. Diagram of the distance dependence of logarithmic amplitude and phase for determining the *D* for Ag_2_Se thin-film sample with In-doping level of *x* = 4%.


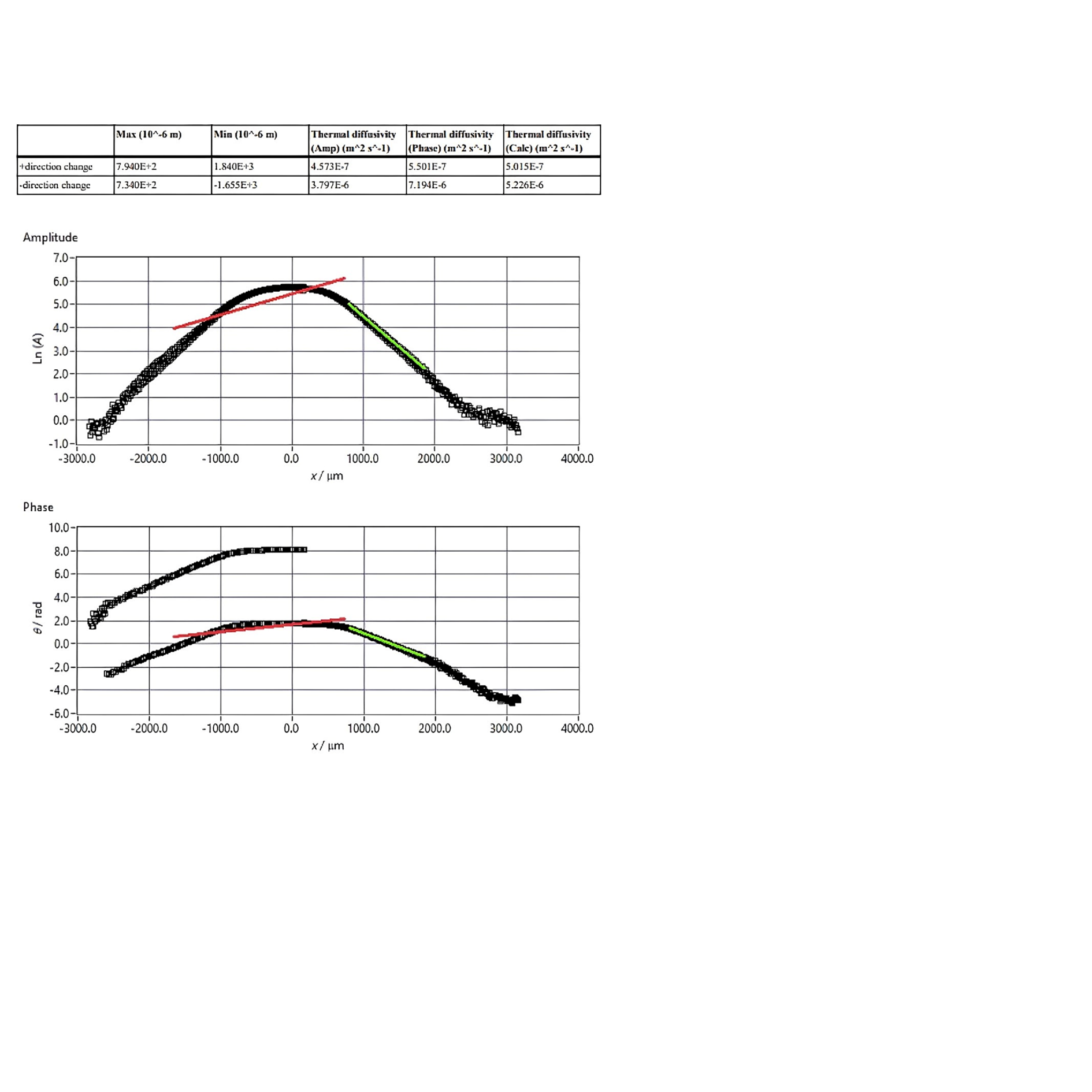


**Figure S32**. Diagram of the distance dependence of logarithmic amplitude and phase for determining the *D* for Ag_2_Se thin-film sample with In-doping level of *x* = 8%.


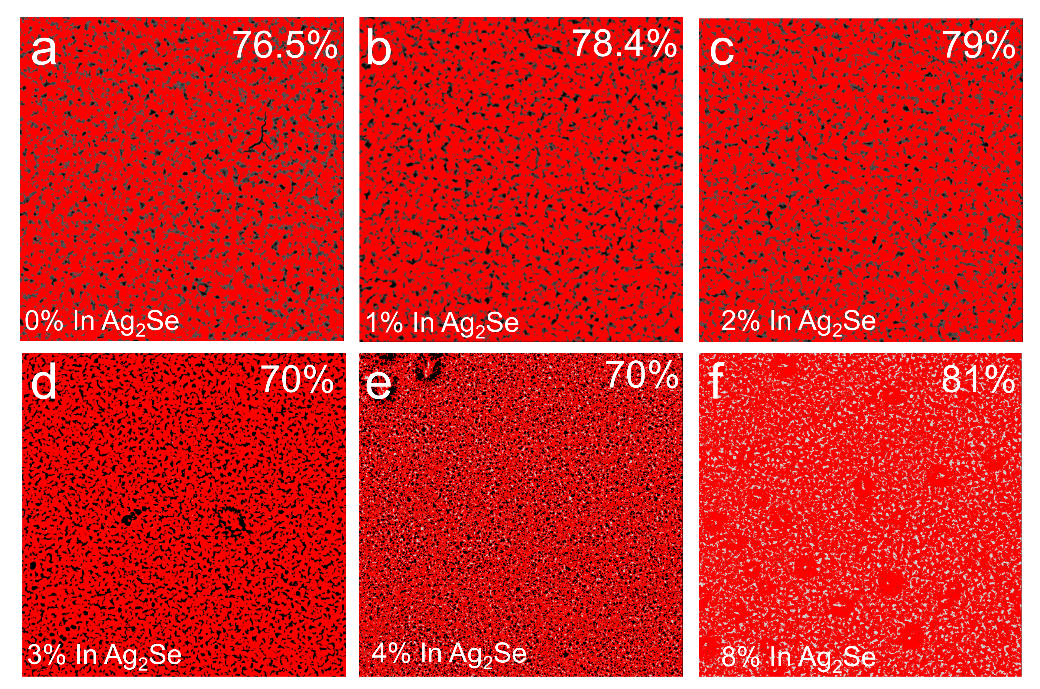


**Figure S33**. Porosity calibration by software ImageJ for Ag_2_Se thin film with different In-doping concentrations (a) *x* = 0%, (b) *x* = 1%, (c) *x* = 2%, (d) *x* = 3%, (e) *x* = 4%, and (f) *x* = 8%. The calibration image derives from SEM image.


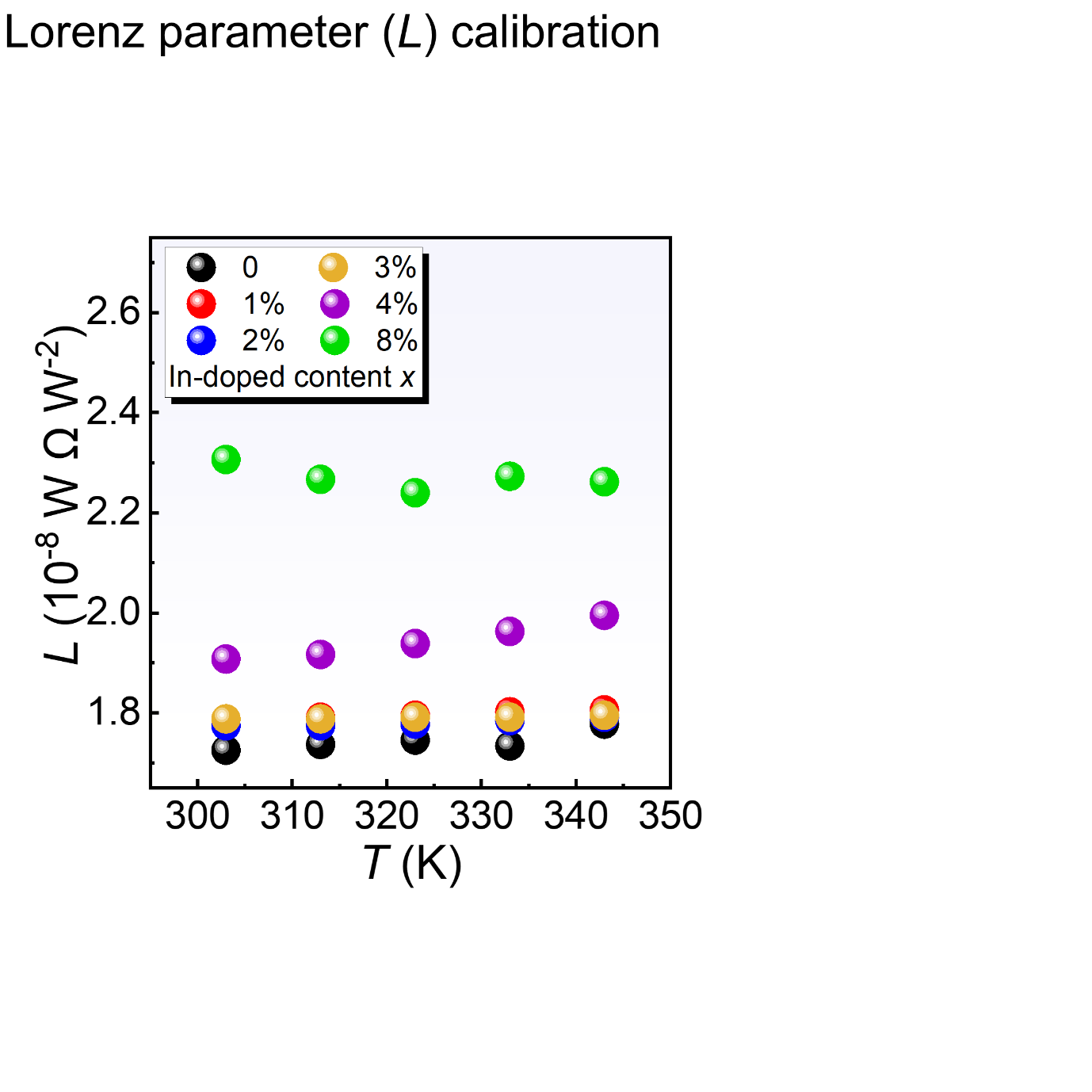


**Figure S34**. Lorenz parameter calibration for Ag_2_Se thin film with different In-doping concentrations.


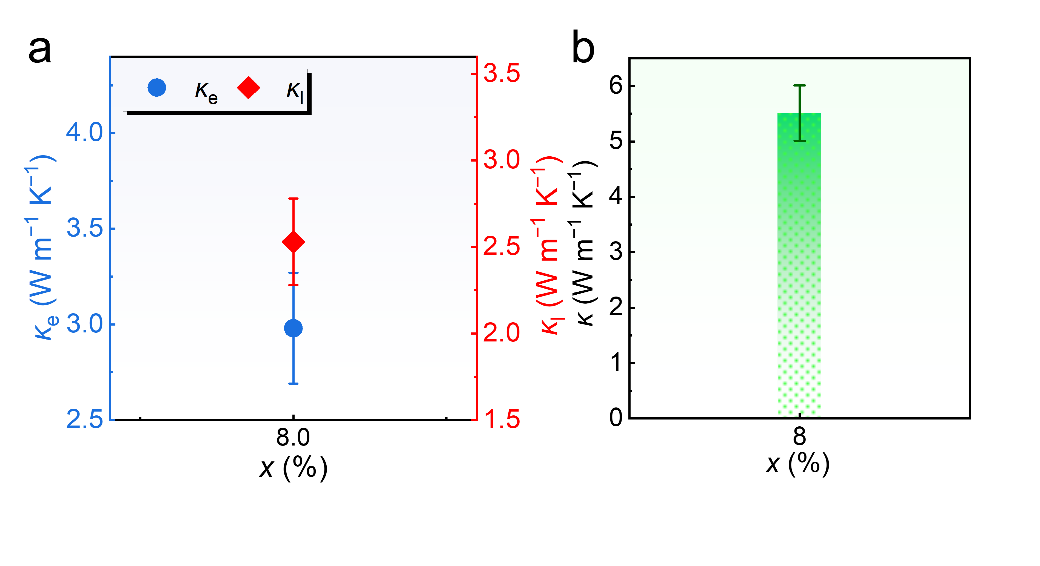


**Figure S35**.Thermal performance for Ag_2_Se thin film sample (*x* = 8%), including (a) electrical thermal conductivity (*κ*_e_) and lattice thermal conductivity (*κ*_l_) and (b) thermal conductivity (*κ*).

**Table S1**. Line scan positions and composition results corresponding to **Figure S18**.

| Position | Distance (nm) | Se (atomic content) | Ag (atomic content) | In (atomic content) |
| --- | --- | --- | --- | --- |
| Linescan-point1 | 0 | 0.392477 | 0.631233 | 0.008013 |
| Linescan-point2 | 49.775 | 0.388551 | 0.632253 | 0.009755 |
| Linescan-point3 | 99.550 | 0.401976 | 0.622055 | 0.010103 |
| Linescan-point4 | 149.325 | 0.396657 | 0.624095 | 0.011932 |
| Linescan-point5 | 199.100 | 0.403116 | 0.622334 | 0.009058 |
| Linescan-point6 | 248.875 | 0.39653 | 0.626227 | 0.009929 |
| Linescan-point7 | 298.650 | 0.404129 | 0.623168 | 0.007577 |

**Table S2**. Point composition results corresponding to **Figure S18**.

| Position | Se (atomic content) | Ag (atomic content) | In (atomic content) |
| --- | --- | --- | --- |
| 12 | 0.404762 | 0.621582 | 0.008624 |
| 13 | 0.402102 | 0.624085 | 0.008188 |
| 14 | 0.399063 | 0.624548 | 0.009843 |
| 15 | 0.399949 | 0.621396 | 0.012108 |
| 16 | 0.397543 | 0.627236 | 0.008188 |
| 17 | 0.399949 | 0.624826 | 0.008885 |
| 18 | 0.39805 | 0.625382 | 0.009669 |
| 19 | 0.398936 | 0.625012 | 0.009408 |
| 20 | 0.39653 | 0.627515 | 0.008711 |
| 21 | 0.393364 | 0.629925 | 0.008624 |
| 22 | 0.409195 | 0.617132 | 0.009756 |
| 23 | 0.411854 | 0.615463 | 0.009408 |
| 24 | 0.403622 | 0.620933 | 0.010017 |
| 25 | 0.397416 | 0.623714 | 0.011672 |
| 26 | 0.410208 | 0.617224 | 0.008972 |
| 27 | 0.403242 | 0.621303 | 0.00993 |
| 28 | 0.404255 | 0.619449 | 0.011063 |
| 29 | 0.402609 | 0.622045 | 0.009669 |
| 30 | 0.399696 | 0.623158 | 0.010714 |
| 31 | 0.39501 | 0.624919 | 0.012108 |
| 32 | 0.407548 | 0.617317 | 0.010627 |
| 33 | 0.402229 | 0.625382 | 0.006794 |
| 34 | 0.409954 | 0.617039 | 0.009408 |

**Table S3**. **A summary of properties of n-type Ag_2_Se-based flexible thermoelectric materials.** Here PI is abbreviated from polyimide, PVP is abbreviated from Polyvinylpyrrolidone, PE is abbreviated from polyethylene, PPy is abbreviated from polypyrrole, PET is abbreviated from polyethylene terephthalate, SWCNTs are abbreviated from single-walled carbon nanotubes, PEDOT is abbreviated from poly(3,4-ethylenedioxythiophene), and PVDF is abbreviated from polyvinylidene fluoride, PDMS is abbreviated from polydimethylsiloxane.

| Materials | Substrate | *S*^2^*σ* (μW cm^–1^ K^–2^) | *T* (K) | *S* (µV K^–1^) | *σ* (S cm^–1^) | *n* (cm^–3^) | *μ* (cm^2^ V^–1^ s^–1^) | *κ* (W m^–1^ K^–1^) | *ZT* | Year | Ref. |
| --- | --- | --- | --- | --- | --- | --- | --- | --- | --- | --- | --- |
| Ag_2-_*_x_*In*_x_*Se | PI | 26.3 | 303 | –134.2 | 1462 | 5.8×10^18^ | 1555 | 0.8 | 0.95 | 2024 | **This work** |
| Ag_2_Se thin film with 3.2 at.% Te | PI | 24.8 | 363 | –132 | 1425 | 1.28×10^19^ | 444 | 0.71 | 1.27 | 2024 | ^[16]^ |
| Ag_2_Se | PI | ~25.9 | 300 | –135 | ~1440 | 6.5×10^18^ | 1250 | ~0.66 | ~1.2 | 2022 | ^[2]^ |
| Ag_2_Se | PI | 22.05 | 300 | –161.7 | 840 | 7.3×10^18^ | 721.3 | 0.61 | 1.1 | 2022 | ^[17]^ |
| Ag_2_Se+PVP | Nylon | 19.1 | 300 | –144 | 925 | 7×10^18^ | 1100 | / | 1.1 | 2020 | ^[18]^ |
| Ag_2_Se/PVP | PI | 25 | 303 | –139 | 1300 | 7×10^18^ | 1180 | 0.71 | 1.05 | 2024 | ^[19]^ |
| Ag_2_Se/PVP | Nylon | 24.78 | 300 | –140 | 1300 | 6.6×10^18^ | / | / | 1.05 | 2024 | ^[19]^ |
| Printed-Ag_2_Se | PET | 17 | 300 | –185 | 460 | / | / | 0.5 | 1.03 | 2020 | ^[20]^ |
| Ag_2_Se | Scaffold | 15.8 | 300 | –183 | 472 | / | / | ~0.47 | ~1 | 2020 | ^[21]^ |
| Ag_2_Se+Se+PPy | Nylon | 22.4 | 300 | –144 | 1064 | 9.5×10^18^ | 762 | / | 0.94 | 2021 | ^[22]^ |
| Ag_2_Se+bacteria cellulose | / | 6.24 | 400 | –167 | 230 | / | / | 0.36 | 0.7 | 2022 | ^[23]^ |
| Ag_2_Se+nylon | Substrate-free | 18.25 | 300 | –137.9 | 958.9 | ~1.2×10^19^ | ~480 | / | 0.68 | 2022 | ^[24]^ |
| Ag_2.02_Se | PI | 21.6 | 348 | ~–110 | ~1785 | / | / | ~1.2 | ~0.6 | 2022 | ^[25]^ |
| Ag_2_Se | Nylon | 9.87 | 300 | –141 | 497 | 3.8×10^18^ | 850 | / | 0.6 | 2019 | ^[26]^ |
| Ag_2_Se/carbon | PI | 16 | 303 | –135 | 1000 | 6.5×10^18^ | 1050 | 0.98 | 0.5 | 2024 | ^[27]^ |
| Ag_2_Se | Substrate-free | ~15.3 | 300 | –120 | 1060 | 4.79×10^18^ | 1390 | ~0.89 | 0.5 | 2023 | ^[28]^ |
| Ag_2_Se | PI | 25.3 | 303 | –135 | 1420 | / | / | / | / | 2024 | ^[29]^ |
| Ag_2.3_Se | Paper | ~24.5 | 303 | –122 | ~1660 | 2×10^19^ | ~415 | / | / | 2020 | ^[30]^ |
| Ag_2_Se | PE | 23 | 300 | –115 | 1700 | 8×10^18^ | 1300 | / | */* | 2024 | ^[31]^ |
| Ag_2_Se+Ag+CuAgSe | Nylon | 22.3 | 300 | –45 | 1050 | 2.7×10^20^ | 250 | / | / | 2019 | ^[32]^ |
| Ag_2_Se | PI | 21.9 | 320 | –142 | 1091 | / | / | / | / | 2023 | ^[33]^ |
| Cu-doped Ag_2_Se | PI | 20.8 | 300 | –125 | ~1350 | 8.6×10^18^ | ~900 | / | / | 2022 | ^[34]^ |
| S-doped Ag_2_Se | PET | 20.58 | 300 | –150 | ~900 | ~1×10^19^ | ~570 | / | / | 2023 | ^[35]^ |
| Ag_2_Se | Nylon | 20.43 | 300 | –150 | ~908 | 5.82×10^18^ | 1277 | / | / | 2022 | ^[36]^ |
| PVP/Ag_2_Se | PI | 20 | 303 | –150 | 300 | / | / | / | / | 2024 | ^[37]^ |
| Ag_2_Se +SWCNTs | Nylon | ~19.4 | 300 | –108.1 | ~1657 | 1.31×10^19^ | / | / | / | 2023 | ^[38]^ |
| Ag_2_Se/S | PI | 19.35 | 303 | –149 | 880 | 4.4×10^18^ | 1150 | / | / | 2024 | ^[39]^ |
| Ag_1.8_Se | PI | 19 | 380 | –138 | 1000 | 7.5×10^18^ | 950 | / | / | 2021 | ^[40]^ |
| Ag_2_Se | Nylon | ~18.8 | 300 | –143 | ~920 | 7.9×10^18^ | 1024 | / | / | 2020 | ^[41]^ |
| Ag_2_Se+Ag | Nylon | ~18.6 | 300 | –67.5 | 3958 | 1.6×10^20^ | ~83 | / | / | 2021 | ^[42]^ |
| Ag_2_Se+CuAgSe+ PEDOT | Nylon | 16 | 300 | –121 | ~1100 | 7.4×10^18^ | 940 | ~0.46 | / | 2020 | ^[43]^ |
| *In-situ* Ag_2.09_Se | / | 15.3 | 423 | –110 | ~1300 | 1.5×10^19^ | 550 | / | / | 2022 | ^[44]^ |
| Ag_2_Se+Ag+PEDOT | Nylon | 14.4 | 300 | –49.5 | ~5957 | 4.4×10^19^ | 78 | / | / | 2021 | ^[45]^ |
| Ag_2_Se+SWCNTs | Nylon | ~12.4 | 397 | –118 | ~880 | 7.8×10^18^ | ~700 | / | / | 2021 | ^[46]^ |
| Ag_1.98_Ga_0.02_Se | Nylon | ~11.6 | 300 | –115 | ~880 | / | / | / | / | 2021 | ^[47]^ |
| S-doped Ag_2_Se | Nylon | ~9.5 | 300 | –106 | 849 | 6×10^18^ | ~900 | / | / | 2022 | ^[48]^ |
| Ag_2_Se/PDMS | PDMS | 5.5 | 303 | –132 | 420 | / | / | / | / | 2024 | ^[49]^ |
| Ag_2_Se+PEDOT | / | ~4.3 | 300 | –91 | 520 | / | / | / | / | 2022 | ^[50]^ |

**References**

[1] E. D. Kolb, R. A. Laudise, *J. Cryst. Growth* **1971**, *8*, 191.

[2] Y. Lei, R. Qi, M. Chen, H. Chen, C. Xing, F. Sui, L. Gu, W. He, Y. Zhang, T. Baba, T. Baba, H. Lin, T. Mori, K. Koumoto, Y. Lin, Z. Zheng, *Adv. Mater.* **2021**, *34*, 2104786.

[3] G. Kresse, J. Hafner, *Phys. Rev. B* **1994**, *49*, 14251.

[4] G. Kresse, J. Hafner, *Phys. Rev. B* **1993**, *47*, 558.

[5] G. Kresse, J. Furthmüller, *Comp. Mater. Sci.* **1996**, *6*, 15.

[6] G. Kresse, J. Hafner, *J. Phys. Condens. Mat.* **1994**, *6*, 8245.

[7] G. Kresse, J. Furthmüller, *Phys. Rev. B* **1996**, *54*, 11169.

[8] G. Kresse, D. Joubert, *Phys. Rev. B* **1999**, *59*, 1758.

[9] J. P. Perdew, K. Burke, M. Ernzerhof, *Phys. Rev. Lett.* **1996**, *77*, 3865.

[10] F. Tran, P. Blaha, *Phys. Rev. Lett.* **2009**, *102*, 226401.

[11] W. Setyawan, S. Curtarolo, *Comp. Mater. Sci.* **2010**, *49*, 299.

[12] X. L. Shi, K. Zheng, M. Hong, W. D. Liu, R. Moshwan, Y. Wang, X.-L. Qu, Z. G. Chen, J. Zou, *Chem. Sci.* **2018**, *9*, 7376.

[13] X. Shi, A. Wu, T. Feng, K. Zheng, W. Liu, Q. Sun, M. Hong, S. T. Pantelides, Z. G. Chen, J. Zou, *Adv. Energy Mater.* **2019**, *9*, 1803242.

[14] X. Shi, A. Wu, W. Liu, R. Moshwan, Y. Wang, Z.-G. Chen, J. Zou, *ACS Nano* **2018**, *12*, 11417.

[15] M. Jin, X.-L. Shi, T. Feng, W. Liu, H. Feng, S. T. Pantelides, J. Jiang, Y. Chen, Y. Du, J. Zou, Z.-G. Chen, *ACS Appl. Mater. Interfaces* **2019**, *11*, 8051.

[16] D. Yang, X.-L. Shi, M. Li, M. Nisar, A. Mansoor, S. Chen, Y. Chen, F. Li, H. Ma, G. X. Liang, X. Zhang, W. Liu, P. Fan, Z. Zheng, Z.-G. Chen, *Nat. Commun.* **2024**, *15*, 923.

[17] M. Saeidi-Javash, K. Wang, M. Zeng, T. Luo, A. W. Dowling, Y. Zhang, *Energy Environ. Sci.* **2022**, *15*, 5093.

[18] C. Jiang, P. Wei, Y. Ding, K. Cai, L. Tong, Q. Gao, Y. Lu, W. Zhao, S. Chen, *Nano Energy* **2021**, *80*, 105488.

[19] Y. Lu, X. Han, P. Wei, Y. Liu, Z. Wang, X. Zuo, W. Zhao, K. Cai, *Chem. Eng. J.* **2024**, *485*, 149793.

[20] M. M. Mallick, A. G. Rösch, L. Franke, A. Gall, S. Ahmad, H. Geßwein, A. Mazilkin, C. Kübel, U. Lemmer, *J. Mater. Chem. A* **2020**, *8*, 16366.

[21] M. M. Mallick, L. Franke, A. G. Rösch, U. Lemmer, *ACS Energy Lett.* **2021**, *6*, 85.

[22] Y. Li, Q. Lou, J. Yang, K. Cai, Y. Liu, Y. Lu, Y. Qiu, Y. Lu, Z. Wang, M. Wu, J. He, S. Shen, *Adv. Funct. Mater.* **2022**, *32*, 2106902.

[23] D. Palaporn, W. Mongkolthanaruk, K. Faungnawakij, K. Kurosaki, S. Pinitsoontorn, *ACS Appl. Energy Mater.* **2022**, *5*, 3489.

[24] Z.-y. Yang, X.-z. Jin, W.-y. Wang, C.-h. Huang, Y.-z. Lei, Y. Wang, *J. Mater. Chem. A* **2022**, *10*, 21080.

[25] Z.-H. Zheng, Y.-L. Li, J.-Y. Niu, M. Wei, D.-L. Zhang, Y.-m. Zhong, M. Nisar, A. Abbas, S. Chen, F. Li, G.-X. Liang, P. Fan, Y.-X. Chen, *J. Mater. Chem. A* **2022**, *10*, 21603.

[26] Y. Ding, Y. Qiu, K. Cai, Q. Yao, S. Chen, L. Chen, J. He, *Nat. Commun.* **2019**, *10*, 841.

[27] M. Zhang, Y. Liu, J. Li, C. Wu, Y. Liu, P. Wei, W. Zhao, K. Cai, *Carbon* **2024**, *229*, 119480.

[28] D. Lee, W. Park, Y. A. Kang, H. T. Lim, S. Park, Y. Mun, J. Kim, K.-S. Jang, *ACS Appl. Mater. Interfaces* **2023**, *15*, 3047.

[29] J. Fu, H. Yu, Y. Wu, Y. Liu, Y. Zheng, X. Zhang, *Thin Solid Films* **2024**, *804*, 140466.

[30] J. Gao, L. Miao, H. Lai, S. Zhu, Y. Peng, X. Wang, K. Koumoto, H. Cai, *iScience* **2020**, *23*, 100753.

[31] H. Ma, S. Pu, H. Wu, S. Jia, J. Zhou, H. Wang, W. Ma, Z. Wang, L. Yang, Q. Sun, *ACS Appl. Mater. Interfaces* **2024**, *16*, 7453.

[32] Y. Lu, Y. Qiu, K. Cai, Y. Ding, M. Wang, C. Jiang, Q. Yao, C. Huang, L. Chen, J. He, *Energy Environ. Sci.* **2020**, *13*, 1240.

[33] P. Sarkar, S. Samanta, D. Das, A. Pathak, V. Putta, P. K. Patro, S. Patra, A. K. Debnath, S. Bhattacharya, K. P. Muthe, A. Singh, *ACS Appl. Electron. Mater.* **2023**, *5*, 1650.

[34] S. Hou, Y. Liu, Y. Luo, X. Wang, L. Yin, X. Sun, Z. Wu, J. Wang, M. Li, Z. Chen, Y. Wang, J. Sui, J. Mao, X. Liu, Q. Zhang, F. Cao, *Cell Rep. Phys. Sci.* **2022**, *3*, 101146.

[35] C. Xin, Z. Fang, S. Jiang, Z. Hu, D. Zhang, F. Cassagne, L. Aigouy, Z. Chen, *Mater. Today Energy* **2023**, *33*, 101266.

[36] Y. Liu, Y. Lu, Z. Wang, J. Li, P. Wei, W. Zhao, L. Chen, K. Cai, *J. Mater. Chem. A* **2022**, *10*, 25644.

[37] S. Kumar, M. Battabyal, K. Sethupathi, D. K. Satapathy, *ACS Appl. Mater. Interfaces* **2024**, *16*, 40848.

[38] Q.-X. Hu, W.-D. Liu, L. Zhang, W. Sun, H. Gao, X.-L. Shi, Y.-L. Yang, Q. Liu, Z.-G. Chen, *Chem. Eng. J.* **2023**, *457*, 141024.

[39] Y. Luo, S. Hou, Y. Liu, X. Sun, Z. Tang, F. Yu, J. Mao, Q. Zhang, F. Cao, *ACS Appl. Mater. Interfaces* **2024**, *16*, 36620.

[40] S. Hou, Y. Liu, L. Yin, C. Chen, Z. Wu, J. Wang, Y. Luo, W. Xue, X. Liu, Q. Zhang, F. Cao, *Nano Energy* **2021**, *87*, 106223.

[41] C. Jiang, Y. Ding, K. Cai, L. Tong, Y. Lu, W. Zhao, P. Wei, *ACS Appl. Mater. Interfaces* **2020**, *12*, 9646.

[42] Q. Gao, W. Wang, Y. Lu, K. Cai, Y. Li, Z. Wang, M. Wu, C. Huang, J. He, *ACS Appl. Mater. Interfaces* **2021**, *13*, 14327.

[43] Y. Lu, Y. Qiu, K. Cai, X. Li, M. Gao, C. Jiang, J. He, *Mater. Today Phys.* **2020**, *14*, 100223.

[44] J. Niu, T. Chen, G. Liang, H. Ma, X. Zhang, P. Fan, Z. Zheng, *Mater. Lett.* **2022**, *312*, 131662.

[45] X. Xia, Q. Zhang, W. Zhou, J. Mei, Z. Xiao, W. Xi, Y. Wang, S. Xie, W. Zhou, *Small* **2021**, *17*, 2102825.

[46] J. Geng, B. Wu, Y. Guo, C. Hou, Y. Li, H. Wang, Q. Zhang, *J. Phys. D Appl. Phys.* **2021**, *54*, 434004.

[47] Y. Lu, Y. Liu, Y. Li, K. Cai, *Compos. Commun.* **2021**, *27*, 100895.

[48] M. Wu, K. Cai, X. Li, Y. Li, Y. Liu, Y. Lu, Z. Wang, W. Zhao, P. Wei, *ACS Appl. Mater. Interfaces* **2022**, *14*, 4307.

[49] H. Wang, X. Lin, G. Han, B. Zhang, Y. Chen, L. Zhang, X. Lu, G. Wang, X. Zhou, *Adv. Funct. Mater.* **2025**, *35*, 2413605.

[50] D. Park, S. Lee, J. Kim, *Compos. Commun.* **2022**, *30*, 101084.
